# Exome chip analyses in adult attention deficit hyperactivity disorder

**DOI:** 10.1038/tp.2016.196

**Published:** 2016-10-18

**Authors:** T Zayats, K K Jacobsen, R Kleppe, C P Jacob, S Kittel-Schneider, M Ribasés, J A Ramos-Quiroga, V Richarte, M Casas, N R Mota, E H Grevet, M Klein, J Corominas, J Bralten, T Galesloot, A A Vasquez, S Herms, A J Forstner, H Larsson, G Breen, P Asherson, S Gross-Lesch, K P Lesch, S Cichon, M B Gabrielsen, O L Holmen, C H D Bau, J Buitelaar, L Kiemeney, S V Faraone, B Cormand, B Franke, A Reif, J Haavik, S Johansson

**Affiliations:** 1 K.G. Jebsen Centre for Neuropsychiatric Disorders, Department of Biomedicine, University of Bergen, Bergen, Norway; 2 Section of Molecular Psychiatry, Clinical Research Unit on Disorders of Neurodevelopment and Cognition Center of Mental Health, University of Wuerburg, Würzburg, Germany; 3 Department of Psychiatry, Psychosomatic Medicine and Psychotherapy, University Hospital Frankfurt, Frankfurt am Main, Germany; 4 Psychiatric Genetics Unit, Group of Psychiatry, Mental Health and Addictions, Vall d'Hebron Research Institute, Universitat Autònoma de Barcelona, Barcelona, Spain; 5 Department of Psychiatry, Hospital Universitari Vall d'Hebron, Barcelona, Spain; 6 Biomedical Network Research Centre on Mental Health (CIBERSAM), Madrid, Spain; 7 Departments of Psychiatry and Legal Medicine, Universitat Autònoma de Barcelona, Barcelona, Spain; 8 Department of Genetics, Universidade Federal do Rio Grande do Sul, Porto Alegre, Brazil; 9 Department of Psychiatry, Universidade Federal do Rio Grande do Sul, Porto Alegre, Brazil; 10 Department of Human Genetics, Donders Institute for Brain, Cognition and Behaviour, Radboud University Medical Center, Nijmegen, The Netherlands; 11 Department of Ophtalmology, Radboud Institute for Molecular Life Sciences, Radboud University Medical Center, Nijmegen, The Netherlands; 12 Department for Health Evidence, Radboud Institute for Health Sciences, Radboud University Medical Center, Nijmegen, The Netherlands; 13 Department of Cognitive Neuroscience, Donders Institute for Brain, Cognition and Behaviour, Radboud University Medical Center, Nijmegen, The Netherlands; 14 Department of Psychiatry, Donders Institute for Brain, Cognition and Behaviour, Radboud University Medical Center, Nijmegen, The Netherlands; 15 Institute of Human Genetics, University of Bonn, Bonn, Germany; 16 Department of Genomics, Life and Brain Center, Bonn, Germany; 17 Department of Biomedicine, University of Basel, Basel, Switzerland; 18 Department of Medical Epidemiology and Biostatistics, Karolinska Institutet, Stockholm, Sweden; 19 NIHR BRC for Mental Health, Institute of Psychiatry, Psychology and Neuroscience and SLaM NHS Trust, King's College London, London, UK; 20 MRC Social Genetic and Developmental Psychiatry Centre, Institute of Psychiatry, Psychology and Neuroscience, King's College London, London, UK; 21 Institute of Neuroscience and Medicine, Structural and Functional Organization of the Brain (INM-1), Research Center Juelich, Juelich, Germany; 22 K.G. Jebsen Center for Genetic Epidemiology, Department of Public Health, NTNU, Norwegian University of Science and Technology, Trondheim, Norway; 23 Department of Laboratory Medicine, Children's and Women's Health, Norwegian University of Science and Technology, Trondheim, Norway; 24 Departments of Psychiatry and of Neuroscience and Physiology, SUNY Upstate Medical University, Syracuse, NY, USA; 25 Departament de Genètica, Microbiologia i Estadística, Facultat de Biologia, Universitat de Barcelona, Barcelona, Spain; 26 Centro de Investigación BiomédicaAnchor en Red de Enfermedades Raras, Barcelona, Spain; 27 Institut de Biomedicina de la Universitat de Barcelona, Barcelona, Spain; 28 Institut de Recerca Pediàtrica HosAnchorpital Sant Joan de Déu, Barcelona, Spain; 29 Division of Psychiatry, Haukeland University Hospital, Bergen, Norway; 30 K.G. Jebsen Centre for Neuropsychiatric Disorders, Department of Clinical Science, University of Bergen, Bergen, Norway; 31 Center for Medical Genetics and Molecular Medicine, Haukeland University Hospital, Bergen, Norway

## Abstract

Attention-deficit/hyperactivity disorder (ADHD) is a highly heritable childhood-onset neuropsychiatric condition, often persisting into adulthood. The genetic architecture of ADHD, particularly in adults, is largely unknown. We performed an exome-wide scan of adult ADHD using the Illumina Human Exome Bead Chip, which interrogates over 250 000 common and rare variants. Participants were recruited by the International Multicenter persistent ADHD CollaboraTion (IMpACT). Statistical analyses were divided into 3 steps: (1) gene-level analysis of rare variants (minor allele frequency (MAF)<1%); (2) single marker association tests of common variants (MAF⩾1%), with replication of the top signals; and (3) pathway analyses. In total, 9365 individuals (1846 cases and 7519 controls) were examined. Replication of the most associated common variants was attempted in 9847 individuals (2077 cases and 7770 controls) using fixed-effects inverse variance meta-analysis. With a Bonferroni-corrected significance level of 1.82E−06, our analyses of rare coding variants revealed four study-wide significant loci: 6q22.1 locus (*P*=4.46E−08), where *NT5DC1* and *COL10A1* reside; the *SEC23IP* locus (*P*=6.47E−07); the *PSD* locus (*P*=7.58E−08) and *ZCCHC4* locus (*P*=1.79E−06). No genome-wide significant association was observed among the common variants. The strongest signal was noted at rs9325032 in *PPP2R2B* (odds ratio=0.81, *P*=1.61E−05). Taken together, our data add to the growing evidence of general signal transduction molecules (*NT5DC1*, *PSD*, *SEC23IP* and *ZCCHC4*) having an important role in the etiology of ADHD. Although the biological implications of these findings need to be further explored, they highlight the possible role of cellular communication as a potential core component in the development of both adult and childhood forms of ADHD.

## Introduction

Attention-deficit/hyperactivity disorder (ADHD) is the most commonly diagnosed childhood-onset neuropsychiatric condition,^1^ persisting into adulthood in up to 60% of the cases.^2^ The adult form of ADHD presents a high risk for developing co-morbid psychiatric conditions, increasing the burden of disease and, consequently, global impairment, resistance to treatment and costs of illness.^3, 4^

ADHD in children and adolescents is highly heritable with an estimated mean heritability of ~76%.^5^ Although genome-wide association studies of ADHD show that ~40% of ADHD's heritability can be accounted for by common variants,^6^ no genome-wide significant (*P*<5.00E−08) single-nucleotide variants (SNVs) have yet been identified. Meta-analyses of some candidate genes and genome-wide association results have yielded interesting hypotheses for this disorder, with pathway analyses pointing towards neurodevelopmental networks.^1^

The mixed results of the previous association studies may be partially due to the polygenic and multifactorial nature of ADHD, with both common and rare variants likely contributing to its etiology.^7, 8^ Phenotypic heterogeneity could be another explanation for the multifarious findings in ADHD genetics. Such heterogeneity could occur when various smaller sets are combined to increase sample size and it may lead to the loss of a real association signal.^9^ Examination of a refined phenotype may reduce phenotypic heterogeneity and increase the probability of detecting a true association signal, although adequately large sample size will still be required to detect associations of small effects.

Given that ADHD is a developmentally heterogeneous condition that covers both a remitting and a persistent adult type of ADHD,^1^ symptom persistence may represent one promising refined phenotype.^10^ As most molecular genetic studies on ADHD so far have been limited to its childhood form only, this might constrain the possibilities to identify genetics of symptom persistence.

In this study, we investigated the role of common and rare exonic polymorphisms in the persistent, adult form of ADHD (aADHD). The focus on low frequency protein-coding variants in addition to the genome-wide common polymorphisms provides an additional source of potentially relevant variation that has received little attention in aADHD genetic studies so far.

## Materials and methods

### Subjects

All adult ADHD cases examined in this study were volunteers enrolled through the International Multicenter persistent ADHD CollaboraTion (IMpACT). All patients were diagnosed with ADHD according to DSM-IV criteria, meaning the presence of ADHD symptoms before 7 years of age. Controls were recruited either at an IMpACT site (Brazil, Germany, The Netherlands, UiB Norway and Spain) or through population studies (Germany, The Netherlands, HUNT Norway and the United Kingdom). All subjects were of European descent who was ensured by means of self-report together with multi-dimensional scaling (Supplementary Appendix 1). In Brazil, only Native Brazilians of European descent were recruited by including only those who reported having grandparents of European descent and by performing a morphological classification based on skin color and morphological traits rather than relying on self-classification only.^11^ The population in Southern Brazil where this study was performed is mainly of European descent and population stratification was not found in the European-derived population of Rio Grande do Sul.^12, 13^ Individuals from Southern Brazil show predominantly European ancestry (94%) according to estimates of interethnic admixture.^13, 14^

All participants provided signed informed consent in accordance with the Declaration of Helsinki. The study was approved by ethics committees in each collaborating country at the corresponding recruitment center: in Germany, by the Ethics Committee at the University of Würzburg (Würzburg, Germany); in Norway, by the regional Ethics Committee for medical and health research ethics, western Norway at the University of Bergen (Bergen, Norway) and the Norwegian Data Inspectorate, the Regional and National Committees for Medical and Health Research Ethics and the Norwegian Directorate of Health at the University of Trondheim (Trondheim, Norway); in Spain, by the Ethics Committee of the Hospital Universitari Vall d'Hebron (HUVH, Barcelona, Spain) at the Department of Psychiatry from the Hospital Universitari Vall d'Hebron (HUVH, Barcelona, Spain); in the Netherlands, by the regional Ethics Committee (Centrale Commissie Mensgebonden Onderzoek: CMO Regio Arnhem—Nijmegen; Protocol number III.04.0403), the Institutional Review Board of the Radboud University Medical Center (Nijmegen, the Netherlands) and by the regional Ethics Committee (Centrale Commissie Mensgebonden Onderzoek: CMO Regio Arnhem Nijmegen; 2008/163; ABR: NL23894.091.08) at the outpatient clinic of GGZ Delfland in Delft, to Parnassia, psycho-medical centre in The Hague or at the department of Psychiatry at the Radboud University Nijmegen Medical Centre in Nijmegen, the Netherlands.; in Brazil, by the Ethics Committee of the Hospital de Clínicas de Porto Alegre (Porto Alegre, Brazil) at the ADHD Outpatient Program at the Hospital de Clínicas de Porto Alegre (HCPA); in UK, by the National Research Ethics Committee at the National Adult ADHD Outpatient Clinic at the South London and Maudsley NHS Trust, (London, UK) and the Wellcome Trust Case Control Consortium (WTCCC).

For the discovery phase of the study, samples were available from four European IMpACT sites: Germany, The Netherlands, Norway and Spain. A replication sample, to examine the most significant SNVs from the common variant analysis identified in the discovery stage, was recruited from the following four IMpACT sites: Brazil, Germany, The Netherlands and the UK. Additional samples were recruited through the WTCCC. A detailed description of all samples is provided in the Supplementary Appendix 1.

### Genotyping, genotype calling and quality control

All subjects included in the discovery stage were genotyped on the InfiniumHumanExome array (Illumina, San Diego, CA, USA). The German cases, the full Dutch sample and the full Spanish sample were genotyped on HumanExome-12v1-1_A, the German controls and the HUNT Norwegian sample of controls were genotyped on HumanExome-12v1_A and the UiB Norwegian sample was genotyped on HumanExome-12v1_B version of the chip. All genotypes were processed using the Illumina GenomeStudio V2011.1 software, with additional genotype assignments implemented in the zCall.^15^ Best practice guidelines were used to perform quality control (QC) of genotype calls in GenomeStudio.^16^ Further QC was carried out on all data sets using PLINK,^17^ with the following steps: (1) genotyping rate threshold was set to 98% both for individuals and for SNVs, (2) Hardy–Weinberg test threshold was set to *P*<1.00E−05, (3) overall heterozygosity of individuals was screened based on common (minor allele frequency (MAF)⩾1%) and rare (MAF<1%) SNVs separately, with outliers defined as those outside the range of mean±3s.d. of the total heterozygosity observed in a sample, (4) relatedness (PI_HAT) threshold was set to 10% and (5) ethnic homogeneity was ensured by means of multi-dimensional scaling with HapMap3 populations. Genotype calling and all QC steps were performed for each data set individually. Those samples that were collected in the same country (namely German cases and German controls, Dutch cases and Dutch controls, Norwegian UiB and HUNT samples) were merged in PLINK and additional QC steps were implemented. Specifically, the screening for heterozygosity, cryptic relatedness and population outliers was performed once more as described above. As the end result, four data sets were produced: a combined German sample, a combined Dutch sample, a combined Norwegian sample and a Spanish sample.

For the replication stage, the most significant three common (MAF>1%) SNVs were genotyped by KASP assay (LGC Genomics, UK) in IMpACT samples (Brazil, Germany, The Netherlands and the UK). The Dutch replication sample was also genotyped on Perlegen (Perlegen Sciences) and HumanCytoSNP-12 array (Illumina).

### Statistical analyses

Statistical analyses were divided into three main stages: (1) examination of rare coding SNVs (MAF<1%), (2) examination of common SNVs (MAF⩾1%) with replication in an independent sample and (3) exploration of biological pathways possibly involved in the development of adult ADHD based on the results from the previous two steps. Prior to the analyses, each subject's genetic substructure characteristics were estimated with principal components analysis implemented in EIGENSTRAT software for each data set individually.^18^ Calculation of principal components was performed based only on individuals and SNVs revealing high genotyping rate (⩾99%) and common variants (MAF⩾1%) after removal of strand ambiguous SNVs and those in high LD (*r*^2^<0.2). Long stretches of LD were also removed prior to calculation of principal components.^19^

### Examination of rare coding variants

Rare variants were defined as those with MAF<1%. The variants were combined per gene and tested for association with adult ADHD in RAREMETAL.^20^ The software first analyzes each data set individually and then combines the generated summary statistics across all the data (meta-analysis). The individual analyses generated single-variant score statistics as well as covariance matrices to reflect linkage disequilibrium (LD) among the variants. We performed two meta-analyses at the gene level: the MAF-weighted burden test and the variable threshold test (VT) across all data sets. Only those genes were tested in which at least two variants were observed across all data sets. All individual tests were adjusted for the number of significant principal components as calculated in EIGENSTRAT. Variants of those genes that reached a *P*-value below 1.00E−05 in the individual association tests were manually evaluated in GenomeStudio in the German, Dutch and Norwegian data sets. GenomeStudio data was not available for the Spanish data set. Single-nucleotide polymorphisms displaying less than optimal clustering were excluded. Bonferroni correction was applied to adjust for multiple testing in the final step of the analyses.

Significant association signals, revealed by these analyses of rare variants, were also examined in the context of association among common variants in meta-analysis of this study as well as in that of Psychiatric Genomics Consortium (PGC).

### Examination of common variants

Common SNVs were defined as those with MAF⩾1%. The variants were tested in PLINK software, assuming an additive model. Each data set was first analyzed separately, including significant EIGENSTRAT principal components as covariates. The results were then combined with the use of fixed-effects inverse variance meta-analysis in METAL,^21^ applying genomic control.^22^ Only those SNVs present in at least two data sets were meta-analyzed. QQ-plots were constructed to ensure proper distribution of observed statistics.

Replication was attempted for the top three SNVs detected in the discovery phase. In the Brazilian, Dutch and German samples, the association of each top variant was assessed in PLINK with logistic regression assuming an additive model. The association in the British sample was assessed by the chi-square test after constructing 2 × 2 contingency tables of allele counts, as we did not have access to raw genotype data of the controls. Each data set was analyzed individually. The results were combined in the fixed-effects inverse variance meta-analysis implemented in PLINK. The three top common variants were also looked up in the largest published ADHD genome-wide association meta-analysis of PGC.

### *In silico* functional analyses of the significant loci

We performed a comprehensive assessment of all rare SNVs contributing to the association signals that survived the study-wide correction for multiple testing to explore their potential functional impact. Such effect was evaluated in two ways: (1) influence of the genetic variation on the encoded protein and (2) regulatory DNA effects (sequences which regulate gene transcription, gene expression and/or DNA replication). The severity of missense SNVs on the function of the encoded proteins was assessed with sequence alignment, using protein blast and protein homology modeling. Applying homology models of human *NT5DC1*, the structural details surrounding the sites of missense mutations were investigated in Discovery studio Visualizer (v 4.1; Illumina). In addition, SIFT and PolyPhen predictions were also carried out in Ensembl Variant Effect Predictor (VEP).

The possible regulatory effects were assessed in RegulomeDB, utilizing genomic annotation databases that employ the data curated by the Encyclopedia of DNA Elements (ENCODE) and provide information on functionally important elements. RegulomeDB identifies DNA features and regulatory elements of the human genome.^23^

### Exploration of biological pathways: GO terms

To evaluate whether any biological processes may be implicated by our results, we performed gene-set analyses using gene ontology (GO) terms. The analyses were performed in MAGMA.^24^ First, a degree of association was calculated for each gene based on both common and rare variants by the use of principal component regression. Rare variants were defined as those with MAF<1% and a burden score was created for each gene, computed as weighted sum of all rare variants in that gene. All data sets were analyzed individually, adjusted for significant EIGENSTRAT components. Fixed effects meta-analysis was then performed, using the square root of the sample sizes as weights. To evaluate each gene's contribution to the examined gene-sets (GO term), the *P*-value of each gene from the meta-analysis was converted to a *Z*-value and used as an outcome variable for a regression model with gene-set membership as a predictor. Gene size, gene-sets' gene density as well as global LD were added as covariates to adjust for possible confounding effects and prevent spurious association. We restricted our pathway analysis only to those sets that contained SNVs in at least 10 genes in our total aADHD data (all four data sets). Bonferroni correction was applied to adjust for multiple testing.

### Gene expression in brain tissue

Gene expression in the human brain across the lifetime was assessed for the loci surviving the Bonferroni correction. Data was accessed from the Human Brain Transcriptome project (http://hbatlas.org), the Expression Atlas (https://www.ebi.ac.uk/gxa/home) and Allen Brain Atlas (http://www.brain-map.org/).

## Results

### Subjects and genotyping

After QC, 9365 individuals (1846 adult ADHD cases and 7519 controls) were available for the analyses. Supplementary Appendix 1 and Table 1 present the details of the four discovery data sets.

### Examination of rare coding variants

As it is still unknown what is the most powerful statistical approach for testing whether a mixture of neutral, risk and protective variants is present within a single gene,^25^ we leaned on the view of rare functional variants being under selective pressure and utilized two methods to explore such scenario: (1) the MAF-weighted burden test that defines rare variants with a fixed threshold and places weights inverse to the MAFs of the variants within a gene and (2) the VT that empirically determines the MAF threshold and makes no prior assumption about the relationship between MAFs and effect sizes of the variants within a gene. Out of 14 431 genes with observed variants, 13 715 genes contained at least two variants. We adjusted our significance level for the use of two methods and the 13 715 genes containing more than two rare SNVs (27 430 tests in total), which yielded a Bonferroni-corrected study-wide significance threshold of 1.82E−06. Four loci survived this correction for multiple testing. The most significant association was observed using the VT method, present at 6q22.1 (*P*=4.46E−08, MAF threshold=0.00027), where the 5′-nucleotidase domain containing 1 *(NT5DC1*) gene resides along with the collagen type X alpha 1 (*COL10A1*) gene. This locus was also the most significant finding using the MAF-weighted burden test (ST1). Supplementary Table 2 presents details of the variants contributing to association signal in 6q22.1 locus.

The three additional study-wide significant associations were observed for the SEC23 interacting protein gene (*SEC23IP*; *P*=6.47E−07, VT method), the pleckstrin and Sec7 domain containing gene (*PSD*; *P*=7.58E−07, MAF-weighted burden test) and zinc finger, CCHC domain containing 4 gene (*ZCCHC4*, *P*=1.79E−06, VT method). ST1 summarizes the most significant genes (*P⩽*1.00E−05) observed in the performed gene-based analyses of rare coding variants.

These four study-wide significant loci revealed no compelling association signals among single point tests of common variants in this study nor in PGC (ST2 and ST3).

### Examination of common variants

The most significant signal in our meta-analysis of common variants was observed at rs9325032 in the *PPP2R2B* gene (odds ratio=0.81, *P*=1.61E−05). No variant reached genome-wide significance (*P*<5.00E−08). ST3 details the top SNVs with association *P*-values <1.00E−03 in the meta-analysis. SF1 depicts the QQ-plots.

Replication was attempted for the top three common SNVs (ST4). No variant reached genome-wide significance (SF2). However, rs9325032 (*PPP2R2B* gene) showed a modest trend of significance (*P*=0.033, ST4A), although significant heterogeneity (for example, differences in estimated MAFs) and discordant direction of effect was observed among samples (ST4A). Comparisons using the data for childhood ADHD collected by the PGC^26^ revealed no significant findings for the top SNVs in the current study (ST4B).

### *In silico* functional analyses of the significant loci

In total, 32 rare coding SNVs contributed to the gene-based association signals passing study-wide Bonferroni correction in the four loci (6q22.1, *SEC23IP*, *PSD* and *ZCCHC4*). No single common variant reached genome-wide significance and, thus, common variants were not assessed further for functionality. First, we evaluated the effect of rare missense variants on the encoded protein in the most significant locus of *NT5DC1*. The NT5DC1 protein contains a known 5′-nucleotidase domain (aa11–337) and a haloacid dehalogenase-like domain (aa198–324). Table 2 summarizes the results of sequence alignment using protein blast and protein homology modeling (template NP_689942.2) to assess the severity of the observed *NT5DC1* missense variants in this locus. Structural inspection of the SNVs in the *NT5DC1* locus revealed their potential to form a possible phosphorylation site (rs150257749) and to induce conformational change (rs150293032) (Table 2 and SF3B), both of which may influence the function of the protein. A homology model of human NT5DC1 and structural inspection of the three missense mutation sites in human *NT5DC1* are presented in SF3. The *NT5DC1* sequence alignment is shown in SF4.

While examining the impact on their respective encoded proteins across all the 32 variants, six were predicted to be deleterious by both PolyPhen and SIFT: one in *NT5DC1* (rs150293032), two in *PSD* (rs142273937 and rs200819772), two in *SEC23IP* (rs142665854 and rs142266445) and one in *ZCCHC4* (rs151252286). Detailed features of these missense variants are presented in ST5.

Apart from surveying the possible direct effects of the variants on their encoded proteins, we also explored their regulatory potential using RegulomeDB. These analyses revealed that the SNVs with the most likely regulatory effect reside within the *PSD* gene (rs147203944, rs148732359 and rs142273937) and probably affect the binding properties of polymerase (RNA) II (DNA directed) polypeptide A (POLR2A) as well as may alter *CTCF* regulatory element binding (rs140739855 and rs200819772) (ST6). In addition, the binding of POLR2A and CTCF may also be affected by the variants in the other two study-wide significant loci: rs73357833 in *SEC23IP*, rs201763036 in *ZCCHC4* (ST6). No significant eQTL effects were observed. Detailed RegulomeBD features of all variants are presented in ST6.

### Exploration of biological pathways: GO terms

To evaluate whether certain biological processes may be implicated by our results, we performed gene-set analyses using GO terms. Among the GO terms, 1.844 terms contained data for 10 or more genes in our data set. This brings the Bonferroni-corrected significance threshold to a *P*-value of 2.17E−05. The strongest association was observed for ‘mRNA 3′-end processing (GO:0031124 term, *P*=1.07E−04). ST7 summarizes the top GO terms (*P⩽*1.00E−03) observed in the meta-analysis of both common and rare variants.

### Gene expression in brain tissue

The expression of the four study-wide significant loci (*NT5DC1*, *SEC23IP*, *PSD* and *ZCCHC4*) was evaluated throughout life in the Human Brain Transcriptome atlas. The transcriptional trajectories of all four loci revealed variability between pre- and post-natal stages, in line with the general patterns of gene expression in the brain across the lifetime.^27^ Supplementary Figure 6 depicts the expression levels of these loci throughout life.

We have also examined the expression patters of the four aforementioned loci with regards to the brain regions. *NT5DC1* revealed the highest expression in substantia nigra as well as other midbrain and hindbrain structures (FANTOM5 and GTEx projects, adult expression levels). The other three loci showed high expression in the cortex and basal ganglia input nuclei (ZCCHC4, FANTOM5 data), and throughout brain (SEC23IP, PSD, mouse data in Allen Brain Atlas).

## Discussion

This study aimed to shed light to the genetic architecture of adult ADHD, the form of this disorder that has received relatively little attention so far. Our main findings are the novel, study-wide significant candidate loci for adult ADHD at 6q22.1, where *NT5DC1* and *COL10A1* reside, as well as the *SEC23IP*, *PSD* and *ZCCHC4* loci.

*NT5DC1* encodes a member of the haloacid dehalogenase superfamily of enzymes, the exact physiological role of which is still largely unknown.^28^ On the basis of its sequence similarity to other haloacid dehalogenases, NT5DC1 may be involved in the de-phosphorylation of intracellular signaling molecules and our *in silico* analyses of the SNVs contributing to its association signal suggest that these variants may alter its function (Table 2). The *NT5DC1* locus, however, also contains a shorter *COL10A1* gene that is embedded in reverse orientation in *NT5DC1* intron 6 (SF5). With our sample size it is not possible to statistically distinguish the effects of these two genes from each other, but the strongest association is seen when testing the larger *NT5DC1* gene (*P*=4.48E−08).

Similarly to 6q22.1 locus, the protein encoded by *SEC23IP* belongs to a family of enzymes as well: intracellular phospholipase A_1_ family that degrades phospholipids and is involved in membrane trafficking.^29^ Altogether with actin cytoskeleton organization, membrane trafficking is also a mechanism through which *PSD*-encoded protein (a guanine nucleotide exchange factor) mediates a number of neuronal functions,^30^ including dendritic spine formation and stabilization.^31^ Furthermore, *ZCCHC4-*encoded zinc-finger DNA-binding protein may also be linked to cell signal transduction as suggested epigenetic silencer of *RAS* gene family.^32, 33^ *RAS* genes reportedly inhibit apoptosis,^33^ a physiological cell death by which unsuccessful neurons are deleted from the central nervous system in the developing brain.^34^

Thus, in this study, we noted association signals in several genes involved in signal transduction, highlighting the possible role of cellular communication in the development of adult ADHD. In line with these observations, previous studies have implicated all four of our study-wide significant loci in disorders of the central nervous system, including ADHD and related neurodevelopmental disorders, ADHD co-morbid conditions as well as syndromes where ADHD symptoms are present.^35, 36, 37, 38, 39, 40, 41, 42, 43, 44, 45, 46, 47, 48, 49, 50, 51^ In addition, paralogs of the *NT5DC1* gene have also been reported to have role in the development of ADHD and other neuropsychiatric disorders.^52, 53, 54^ These findings fit with the higher transcript level of *NT5DC1* reported in substantia nigra, a major localization of dopaminergic neurons.

Despite being the largest genetic study on adult ADHD, it is still likely to be underpowered to detect variants with very small effect sizes. Thus, no genome-wide significant signal was observed for common polymorphisms (MAF⩾1%). However, gene-based statistical methods to analyze rare variants combined could potentially improve power.^55^ These aggregation tests are based on various assumptions about the underlying genetic architecture and their power depends on the true, and, in case of ADHD, unknown, disease model.^55^ In this study, we used two methods to detect gene-based association: (1) the VT method that leans on the view of functional allelic variants being a subject to purifying selection pressure and empirically calculates a threshold below which the variants are more likely to be functional, based on the observed data; and (2) MAF-weighted burden test, which makes the same assumption of functional variants being under selective pressure as the VT method. As all the four study-wide significant loci reveal association signals with both methods (ST1B), it may suggest that the results appear to be robust with regard to different gene-based tests operating under the same hypothesis and that such applications may help us in uncovering underlying genetic architecture of such complex disorders as ADHD.

Taken together, all four study-wide significant loci have been implicated in the function of neural circuitry and communication, cellular mechanisms previously linked to the development of mental disorders,^56^ including childhood ADHD.^1^ Thus, dysregulation of cellular communication could be a core component in the development of both adult and childhood forms of ADHD. Nonetheless, these findings should be subjected to further examination in larger samples before their role in ADHD can be firmly established.

## Figures and Tables

**Table 1:** Properties of the IMpACT discovery samples examined in this study

*Individuals*			
IMpACT site	Cases	Controls	Total
Germany	340	2.286	2.626
Netherlands	294	1.703	1.997
Norway	597	2.598	3.195
Spain	615	932	1.547
Total	1.846	7.519	9.365
			
*Variants*			
IMpACT site	Rare (MAF<1%)	Common (MAF⩾1%)	Total
Germany	71.350	33.053	104.403
Netherlands	59.415	33.336	92.751
Norway	60.649	33.870	94.519
Spain	62.559	31.564	94.123

Abbreviation: MAF, minor allele frequency.

**Table 2:** Summary of effect severity of the rare coding missense variants in the *NT5DC1* gene with observed minor allele frequency below the established critical threshold

*SNV*	*Mutation*	*AA sequence Conservation*	*Predicted structure*	*Comments*
rs201990931	V84I	Conserved	β-sheet (end)	Part of a hydrophobic cluster, but Ile is likely to fit well
rs150257749	N151S	N(59)/S(7)/D(9)/-(3)	Loop	Possible H-bond with K154, conserved with S. Possible formation of phosphorylation site
rs150293032	G302D	G(73)/S(6)/N(2)	Loop	Neighboring basic amino acid—D may cause conf. change. T301 plausible phosphorylation site (PKA/PKG/MSK)

Abbreviation: SNV, single-nucleotide variants.

The severity of missense SNVs on the function of the encoded protein was assessed with sequence alignment, using protein BLAST and protein homology modeling (SF3). The mutations investigated are shown in column two along with the amino acid (AA) conservation based on the sequence alignment (SF4). Single letter amino acid codes are used; valine (V), isoleucine (I), asparagine (N), serine (S), aspartic acid (D), glycine (G) and threonine (T).
