# Supplementary material for: Exome chip analyses in adult attention deficit hyperactivity disorder
Source: Transl Psychiatry. 2016 Oct 18;6(10):e923–. doi: 10.1038/tp.2016.196 (PMC5315553; doi:10.1038/tp.2016.196)
Supplement: Supplementary Table 1 [file tp2016196x1.docx]

**Supplementary Table 1. Top genes (p≤1.00E-05) in the gene-based analyses of rare variants (MAF<1%).**

1. Summary of the top genes (p≤1.00E-05) in the analyses of rare variants (MAF<1%)

Study-wide Bonferroni-corrected significance threshold was set to 1.82E-06. Genes surviving this correction are highlighted in bold.

| **Gene Symbol** | **Number of rare variants observed under the specified MAF threshold** | **Gene-based p-value** | **Method of examination (MAF threshold for variant inclusion)** |
| --- | --- | --- | --- |
| **NT5DC1** | **5** | **4.48E-08** | **Variable threshold (0.00027)** |
| **SEC23IP** | **6** | **6.47E-07** | **Variable threshold (0.000053)** |
| **COL10A1** | **2** | **1.10E-06** | **Variable threshold (0.00027)** |
| **ZCCHC4** | **9** | **1.79E-06** | **Variable threshold (0.00064)** |
| NLGN1 | 3 | 6.92E-06 | Variable threshold (0.00016) |
| PSD | 9 | 7.95E-06 | Variable threshold (0.00091) |
| **NT5DC1** | **16** | **5.91E-07** | **MAF-weighted burden (0.01)** |
| **PSD** | **12** | **7.58E-07** | **MAF-weighted burden (0.01)** |
| BCAT1 | 5 | 4.86E-06 | MAF-weighted burden (0.01) |
| COL10A1 | 10 | 5.70E-06 | MAF-weighted burden (0.01) |

B) P-values of association signals observed in each method of rare variants (MAF<1%) analyses in the four study-wide significant loci.

| Gene | P-values of each method of analyses (MAF threshold, number of SNPs observed under the specified MAF threshold) | |
| --- | --- | --- |
|  | MAF-weighted burden | Variable Threshold |
| NT5DC1 | 5.91E-07  (0.01, 16) | 4.48E-08 (0.00027, 5) |
| SEC23IP | 2.05E-04  (0.01, 12) | 6.47E-07 (5.34E-05, 6) |
| PSD | 7.58E-07  (0.01, 12) | 7.95E-06 (0.00091, 9) |
| COL10A1 | 5.70E-06  (0.01, 10) | 1.10E-06  (0.00027, 2) |
| ZCCHC4 | 6.68E-04  (0.01, 12) | 1.79E-06  (0.00064, 9) |
