# Supplementary material for: Exome chip analyses in adult attention deficit hyperactivity disorder
Source: Transl Psychiatry. 2016 Oct 18;6(10):e923–. doi: 10.1038/tp.2016.196 (PMC5315553; doi:10.1038/tp.2016.196)
Supplement: Supplementary Table 2 [file tp2016196x2.docx]

**Supplementary Table 2. Association signals observed in Psychiatric Genomics Consortium (PGC)^1^of common variants observed in the four study-wide significant loci based on the analysis of rare variants.**

| SNP | Locus | Reference Allele | zscore | P value |
| --- | --- | --- | --- | --- |
| rs476330 | 6q22.1 | T | 2.418 | 0.0156 |
| rs958738 | 6q22.1 | A | 2.212 | 0.02699 |
| rs493467 | 6q22.1 | A | -2.208 | 0.02726 |
| rs564253 | 6q22.1 | T | -2.161 | 0.03067 |
| rs9320557 | 6q22.1 | A | 2.158 | 0.03092 |
| rs534932 | 6q22.1 | T | -2.129 | 0.03329 |
| rs563962 | 6q22.1 | A | 2.127 | 0.03338 |
| rs563176 | 6q22.1 | A | 2.127 | 0.03345 |
| rs2145357 | 6q22.1 | A | 2.082 | 0.03731 |
| rs482012 | 6q22.1 | T | 2.073 | 0.03819 |
| rs12196141 | 6q22.1 | A | 2.068 | 0.03861 |
| rs1931897 | 6q22.1 | A | 2.028 | 0.04252 |
| rs11752488 | 6q22.1 | T | 1.936 | 0.05291 |
| rs9488845 | 6q22.1 | A | -1.912 | 0.05583 |
| rs509002 | 6q22.1 | T | -1.809 | 0.07047 |
| rs9488836 | 6q22.1 | T | -1.785 | 0.07424 |
| rs471766 | 6q22.1 | T | 1.712 | 0.08689 |
| rs1064583 | 6q22.1 | A | 1.687 | 0.09162 |
| rs509859 | 6q22.1 | T | -1.672 | 0.09449 |
| rs12193974 | 6q22.1 | T | 1.443 | 0.149 |
| rs1204804 | 6q22.1 | A | 1.274 | 0.2026 |
| rs485599 | 6q22.1 | A | -1.248 | 0.2122 |
| rs9387383 | 6q22.1 | A | 1.221 | 0.2223 |
| rs1204846 | 6q22.1 | A | -1.206 | 0.2279 |
| rs1204829 | 6q22.1 | T | 1.201 | 0.2296 |
| rs1204796 | 6q22.1 | T | 1.198 | 0.2308 |
| rs1204787 | 6q22.1 | C | 1.186 | 0.2354 |
| rs4945551 | 6q22.1 | T | 1.104 | 0.2696 |
| rs7769748 | 6q22.1 | T | 1.048 | 0.2946 |
| rs9488866 | 6q22.1 | A | 0.983 | 0.3254 |
| rs1999658 | 6q22.1 | A | -0.96 | 0.3371 |
| rs1204826 | 6q22.1 | A | -0.835 | 0.4036 |
| rs549332 | 6q22.1 | A | -0.81 | 0.4178 |
| rs1048920 | 6q22.1 | A | 0.757 | 0.4492 |
| rs7755367 | 6q22.1 | T | -0.746 | 0.4559 |
| rs1204817 | 6q22.1 | A | -0.509 | 0.6104 |
| rs1204836 | 6q22.1 | A | -0.493 | 0.6217 |
| rs1204785 | 6q22.1 | A | -0.462 | 0.6441 |
| rs15679 | 6q22.1 | A | -0.412 | 0.6806 |
| rs17077574 | 6q22.1 | C | -0.29 | 0.7719 |
| rs12526817 | 6q22.1 | T | -0.274 | 0.7838 |
| rs11153598 | 6q22.1 | A | -0.27 | 0.787 |
| rs6914678 | 6q22.1 | A | 0.191 | 0.8486 |
| rs1204788 | 6q22.1 | A | -0.074 | 0.9408 |
| rs1204798 | 6q22.1 | A | 0.032 | 0.9744 |
| rs1204843 | 6q22.1 | A | 0.025 | 0.9797 |
| rs742930 | 6q22.1 | A | -0.013 | 0.9898 |
| rs3781293 | PSD | T | 0.737 | 0.461 |
| rs1056890 | PSD | A | -0.72 | 0.4716 |
| rs2282295 | PSD | A | 0.214 | 0.8308 |
| rs12260430 | PSD | T | 0.064 | 0.9486 |
| rs12771873 | SEC23IP | A | -2.274 | 0.02294 |
| rs2279939 | SEC23IP | A | -2.174 | 0.02968 |
| rs3740570 | SEC23IP | T | 2.064 | 0.03901 |
| rs2271123 | SEC23IP | A | 2.063 | 0.03912 |
| rs2279941 | SEC23IP | A | 2.059 | 0.03945 |
| rs17099368 | SEC23IP | T | 2.058 | 0.03964 |
| rs2279940 | SEC23IP | A | 2.055 | 0.03986 |
| rs2475301 | SEC23IP | T | -1.71 | 0.08732 |
| rs2475298 | SEC23IP | A | -1.676 | 0.09379 |
| rs2901218 | SEC23IP | A | -1.268 | 0.2047 |
| rs7895684 | SEC23IP | A | -1.132 | 0.2575 |
| rs4752352 | SEC23IP | T | -1.111 | 0.2665 |
| rs2271124 | SEC23IP | A | -1.107 | 0.2683 |
| rs4752351 | SEC23IP | T | -1.028 | 0.304 |
| rs1052289 | SEC23IP | T | 1.015 | 0.3099 |
| rs6585564 | SEC23IP | A | -0.252 | 0.801 |
| rs10788000 | SEC23IP | A | -0.218 | 0.8276 |
| rs17099385 | SEC23IP | T | -0.089 | 0.9291 |
| rs12241478 | SEC23IP | T | -0.012 | 0.9907 |
| rs316783 | ZCCHC4 | A | 1.676 | 0.09379 |
| rs13105472 | ZCCHC4 | A | 1.101 | 0.2709 |
| rs13102920 | ZCCHC4 | T | 1.04 | 0.2985 |
| rs10009504 | ZCCHC4 | A | 0.989 | 0.3224 |
| rs13114588 | ZCCHC4 | A | 0.968 | 0.3333 |
| rs12508686 | ZCCHC4 | T | 0.95 | 0.3422 |
| rs4697567 | ZCCHC4 | A | 0.799 | 0.4244 |
| rs12642624 | ZCCHC4 | A | 0.795 | 0.4265 |
| rs2667298 | ZCCHC4 | A | 0.767 | 0.4431 |
| rs316779 | ZCCHC4 | T | 0.758 | 0.4487 |
| rs315674 | ZCCHC4 | T | 0.712 | 0.4766 |
| rs315688 | ZCCHC4 | A | 0.701 | 0.4834 |
| rs13149511 | ZCCHC4 | A | -0.695 | 0.4873 |
| rs316777 | ZCCHC4 | C | -0.639 | 0.5229 |
| rs879008 | ZCCHC4 | T | 0.532 | 0.5949 |
| rs1963656 | ZCCHC4 | T | -0.522 | 0.6017 |
| rs6832360 | ZCCHC4 | T | 0.48 | 0.6313 |
| rs316780 | ZCCHC4 | T | 0.149 | 0.8814 |
| rs16877042 | ZCCHC4 | T | 0.097 | 0.9229 |
| rs16877048 | ZCCHC4 | A | 0.091 | 0.9276 |
| rs316776 | ZCCHC4 | T | -0.06 | 0.9519 |
| rs316775 | ZCCHC4 | A | -0.046 | 0.963 |
| rs316800 | ZCCHC4 | A | 0.039 | 0.9686 |
| rs12642512 | ZCCHC4 | A | 0.039 | 0.9688 |
| rs315690 | ZCCHC4 | A | 0.039 | 0.9688 |
| rs184360 | ZCCHC4 | A | -0.03 | 0.9761 |
| rs188819 | ZCCHC4 | C | 0.023 | 0.9817 |
| rs16877032 | ZCCHC4 | A | -0.009 | 0.9931 |

1. 1. Neale BM, Medland SE, Ripke S, Asherson P, Franke B, Lesch KP *et al.* Meta-analysis of genome-wide association studies of attention-deficit/hyperactivity disorder. *J Am Acad Child Adolesc Psychiatry* 2010; **49**(9)**:** 884-897.
