# Supplementary material for: Exome chip analyses in adult attention deficit hyperactivity disorder
Source: Transl Psychiatry. 2016 Oct 18;6(10):e923–. doi: 10.1038/tp.2016.196 (PMC5315553; doi:10.1038/tp.2016.196)
Supplement: Supplementary Table 3 [file tp2016196x3.docx]

**Supplementary Table 3. The top SNVs with association p-values less than 1.00E-03 in meta-analysis of common variants (MAF≥1%).**

“Effect” reflects the meta-analyzed beta for the each copy of the “Effect Allele” based on the individual tests of logistic regression in each of the four discovery IMpACT datasets. “SE” refers to standard error of the beta. “P-value” reflects the strength of association in of meta-analysis. “Direction” reflects the direction of association in individual discovery IMpACt datasets. “HetP-value” reflects the level of heterogeneity among the examined datasets of IMpACT. “NMD” stands for nonsense-mediated mRNA decay. Bonferroni-corrected study-wide significance threshold was set to 1.48E-06.

| **SNV** | **Gene** | **Function** | **Effect Allele** | **Frequency of the Effect Allele** | **Effect** | **SE** | **P-value** | **Direction** | **HetP-Value** |
| --- | --- | --- | --- | --- | --- | --- | --- | --- | --- |
| rs9325032 | PPP2R2B | intronic, NMD transcript variant | A | 0.7583 | 0.2144 | 0.0497 | 1.61E-05 | ++++ | 0.5063 |
| rs3095150 | intergenic | NA | T | 0.63 | -0.1733 | 0.0453 | 0.0001 | ---- | 0.2755 |
| rs117611723 | SYNRG | missense | T | 0.0178 | 0.5546 | 0.1461 | 0.0001 | ++++ | 0.6785 |
| rs12904234 | IREB2 | intronic, NMD transcript variant | A | 0.6146 | 0.1695 | 0.0436 | 0.0001 | ++++ | 0.6464 |
| rs13180 | IREB2 | synonymous | T | 0.6127 | 0.1688 | 0.0435 | 0.0001 | ++++ | 0.6388 |
| rs2319850 | DDX60L | missense | T | 0.0459 | 0.3599 | 0.0952 | 0.0001 | ++++ | 0.3246 |
| rs61745224 | ZNF274 | missense | C | 0.9807 | -0.5859 | 0.1598 | 0.0002 | ?--- | 0.5208 |
| rs35599968 | DCHS1 | missense | C | 0.2814 | -0.2758 | 0.0776 | 0.0004 | ---- | 0.3868 |
| rs6964421 | KDELR2 | intronic, NMD transcript variant | A | 0.3115 | -0.1576 | 0.0446 | 0.0004 | ---- | 0.6642 |
| rs76824703 | R3HCC1 | missense | T | 0.03 | 0.4022 | 0.1141 | 0.0004 | ++-+ | 0.3885 |
| rs10501293 | intergenic | transcript variant of a non-coding RNA | A | 0.7406 | 0.1666 | 0.0477 | 0.0004 | ++++ | 0.9735 |
| rs6992851 | LOC105377788 | intronic | A | 0.0589 | -0.3358 | 0.0962 | 0.0004 | ---- | 0.3055 |
| rs73888252 | ATP13A5 | missense | T | 0.1028 | -0.2504 | 0.0719 | 0.0005 | ---- | 0.8133 |
| rs1595406 | intergenic | transcript variant of a non-coding RNA | A | 0.6783 | 0.1547 | 0.0449 | 0.0006 | ++++ | 0.8545 |
| rs7252027 | EID2 | missense | A | 0.2313 | 0.1669 | 0.0485 | 0.0006 | ++++ | 0.3126 |
| rs6590942 | CEP126 | missense | A | 0.9699 | -0.3952 | 0.115 | 0.0006 | ---- | 0.8695 |
| rs61733666 | ZNF415 | missense | T | 0.9624 | -0.3527 | 0.1034 | 0.0006 | ---- | 0.4053 |
| rs41283313 | KNDC1 | missense | A | 0.9651 | -0.4252 | 0.1247 | 0.0006 | ?--+ | 0.08606 |
| rs365936 | BLOC1S5-TXNDC5 | intronic, NMD transcript variant | T | 0.5832 | 0.1415 | 0.0421 | 0.0008 | ++++ | 0.5574 |
| rs534892 | VARS | 5UTR | C | 0.0642 | 0.3094 | 0.0921 | 0.0008 | ++++ | 0.5537 |
| rs2303578 | EIF3J | missense | A | 0.024 | 0.4288 | 0.1278 | 0.0008 | ++++ | 0.1089 |
| rs2302677 | RPGRIP1L | missense | A | 0.0309 | 0.3729 | 0.1113 | 0.0008 | ++++ | 0.7513 |
| rs12322164 | TMEM117 | intronic, NMD transcript variant | A | 0.1652 | 0.1824 | 0.0547 | 0.0008 | ++++ | 0.4822 |
| rs2043112 | RICTOR | missense | A | 0.4184 | -0.1391 | 0.0422 | 0.0009 | ---- | 0.4737 |
