# Supplementary material for: Exome chip analyses in adult attention deficit hyperactivity disorder
Source: Transl Psychiatry. 2016 Oct 18;6(10):e923–. doi: 10.1038/tp.2016.196 (PMC5315553; doi:10.1038/tp.2016.196)
Supplement: Supplementary Table 4 [file tp2016196x4.docx]

**Supplementary Table 4. The most significant three variants observed in the common variants (MAF>1%) analysis and their replication.**

A) Discovery and replication performed in this study

“HetP-value” reflects the level of heterogeneity among the examined datasets. “Observed MAF” refers to the minor allele frequency observed in controls. rs117611723 failed quality control in the controls in the UK sample.

| **rs9325032 (*PPP2R2B* gene)** | | | | | | | | |
| --- | --- | --- | --- | --- | --- | --- | --- | --- |
|  | DISCOVERY | | | | | | | |
| IMpACT site | N cases | N controls | Observed MAF | Effect Allele | OR | 95% CI | p-value | HetP-value |
| Germany | 340 | 2,286 | 0.228 | G | 0.87 | 0.71 - 1.06 | 0.167 | NA |
| Netherlands | 294 | 1703 | 0.231 | G | 0.78 | 0.63 - 0.97 | 0.028 | NA |
| Norway | 597 | 2598 | 0.238 | G | 0.73 | 0.61 - 0.87 | 4.41E-04 | NA |
| Spain | 615 | 932 | 0.260 | G | 0.86 | 0.73 - 1.02 | 0.089 | NA |
| Discovery Meta | 1846 | 7519 | NA | G | 0.81 | 0.73 - 0.89 | 1.61E-05 | 0.506 |
|  | REPLICATION | | | | | | | |
| IMpACT site | N cases | N controls | Observed MAF | Effect Allele | OR | 95% CI | p-value | HetP-value |
| Brazil | 533 | 553 | 0.258 | G | 1.05 | 0.87 - 1.28 | 0.592 | NA |
| Germany | 919 | 984 | 0.248 | G | 0.78 | 0.67 - 0.90 | 9.56E-04 | NA |
| Netherlands + IMAGE | 229 | 270 | 0.276 | G | 0.78 | 0.73 - 1.06 | 0.109 | NA |
| United Kingdom | 396 | 5963 | 0.237 | G | 1.03 | 0.87 - 1.22 | 0.778 | NA |
| Replication Meta | 2077 | 7770 | NA | G | 0.90 | 0.82 – 0.99 | 0.033 | 0.025 |
| **rs3095150 (intergenic)** | | | | | | | | |
|  | DISCOVERY | | | | | | | |
| IMpACT site | N cases | N controls | Observed MAF | Effect Allele | OR | 95% CI | p-value | HetP-value |
| Germany | 340 | 2.286 | 0.377 | C | 1.14 | 0.95 - 1.36 | 0.159 | NA |
| Netherlands | 294 | 1703 | 0.362 | C | 1.36 | 1.13 - 1.62 | 8.58E-03 | NA |
| Norway | 597 | 2598 | 0.342 | C | 1.24 | 1.03 - 4.14 | 0.0253 | NA |
| Spain | 615 | 932 | 0.389 | C | 1.08 | 0.93 - 1.26 | 0.308 | NA |
| Discovery Meta | 1846 | 7519 | NA | C | 1.19 | 1.09 - 1.30 | 1.00E-04 | 0.275 |
|  | REPLICATION | | | | | | | |
| IMpACT site | N cases | N controls | Observed MAF | Effect Allele | OR | 95% CI | p-value | HetP-value |
| Brazil | 533 | 553 | 0.398 | C | 1.00 | 0.85 - 1.19 | 0.961 | NA |
| Germany | 919 | 984 | 0.385 | C | 1.05 | 0.92 - 1.19 | 0.451 | NA |
| Netherlands + IMAGE | 229 | 270 | 0.378 | C | 0.93 | 0.73 - 1.20 | 0.560 | NA |
| United Kingdom | 396 | 5963 | 0.361 | C | 1.13 | 0.98 - 1.31 | 0.103 | NA |
| Replication Meta | 2077 | 7770 | NA | C | 1.05 | 0.97 – 1.14 | 0.219 | 0.547 |
| **rs117611723 (*SYNRG* gene)** | | | | | | | | |
|  | DISCOVERY | | | | | | | |
| IMpACT site | N cases | N controls | Observed MAF | Effect Allele | OR | 95% CI | p-value | HetP-value |
| Germany | 340 | 2.286 | 0.017 | T | 1.87 | 1.12 - 3.15 | 0.017 | NA |
| Netherlands | 294 | 1703 | 0.017 | T | 1.33 | 0.74 - 2.39 | 0.334 | NA |
| Norway | 597 | 2598 | 0.012 | T | 1.57 | 0.81 - 3.05 | 0.178 | NA |
| Spain | 615 | 932 | 0.022 | T | 2.12 | 1.29 - 3.47 | 3.00E-03 | NA |
| Discovery Meta | 1846 | 7519 | NA | T | 1.74 | 1.31 - 2.32 | 1.00E-04 | 0.678 |
|  | REPLICATION | | | | | | | |
| IMpACT site | N cases | N controls | Observed MAF | Effect Allele | OR | 95% CI | p-value | HetP-value |
| Brazil | 533 | 553 | 0.014 | T | 1.42 | 0.75 - 2.69 | 0.284 | NA |
| Germany | 919 | 984 | 0.014 | T | 1.21 | 0.72 - 2.02 | 0.463 | NA |
| Netherlands + IMAGE | 229 | 270 | 0.009 | T | 1.19 | 0.34 - 4.18 | 0.781 | NA |
| United Kingdom | NA | NA | NA | NA | NA | NA | NA | NA |
| Replication Meta | 1681 | 1807 | NA | T | 1.28 | 0.87 – 1.87 | 0.204 | 0.925 |

B) Look up of the top three common variants in ADHD GWA data of Psychiatric Genomics Consortium (PGC) (1)

| MarkerName | Allele1 | Allele2 | Z score | P-value |
| --- | --- | --- | --- | --- |
| rs9325032 | T | C | -0.375 | 0.708 |
| rs117611723 | Not reported | Not reported | Not reported | Not reported |
| rs3095150 | Not reported | Not reported | Not reported | Not reported |

1. Neale BM, Medland SE, Ripke S, Asherson P, Franke B, Lesch KP, et al. Meta-analysis of genome-wide association studies of attention-deficit/hyperactivity disorder. J Am Acad Child Adolesc Psychiatry. 2010;49(9):884-97.
