# Supplementary material for: Exome chip analyses in adult attention deficit hyperactivity disorder
Source: Transl Psychiatry. 2016 Oct 18;6(10):e923–. doi: 10.1038/tp.2016.196 (PMC5315553; doi:10.1038/tp.2016.196)
Supplement: Supplementary Table 5 [file tp2016196x5.docx]

**Supplementary Table 5. Details of the 32 rare coding variants contributing to the Bonferroni-corrected significant gene-based association signals in this study with respect to the encoded proteins.**

| SNV | Allele | Consequence | Gene_Symbol | Gene | Transcript | BIOTYPE | cDNA position | CDS position | Protein position | Amino_acids | Codons | SIFT | PolyPhen |
| --- | --- | --- | --- | --- | --- | --- | --- | --- | --- | --- | --- | --- | --- |
| rs146980198 | A | missense | COL10A1 | ENSG00000123500 | ENST00000327673 | protein_coding | 1669 | 1261 | 421 | P/S | Cca/Tca | tolerated(0.07) | unknown(0) |
|  | A | missense | COL10A1 | ENSG00000123500 | ENST00000243222 | protein_coding | 1357 | 1261 | 421 | P/S | Cca/Tca | tolerated(0.07) | unknown(0) |
| rs151327195 | T | missense | COL10A1 | ENSG00000123500 | ENST00000327673 | protein_coding | 821 | 413 | 138 | R/Q | cGg/cAg | tolerated(0.09) | unknown(0) |
|  | T | missense | COL10A1 | ENSG00000123500 | ENST00000452729 | protein_coding | 496 | 413 | 138 | R/Q | cGg/cAg | tolerated(0.23) | unknown(0) |
|  | T | missense | COL10A1 | ENSG00000123500 | ENST00000243222 | protein_coding | 509 | 413 | 138 | R/Q | cGg/cAg | tolerated(0.09) | unknown(0) |
| rs150257749 | G | missense | NT5DC1 | ENSG00000178425 | ENST00000319550 | protein_coding | 534 | 452 | 151 | N/S | aAt/aGt | tolerated(0.33) | benign(0.001) |
|  | G | missense | NT5DC1 | ENSG00000178425 | ENST00000419791 | protein_coding | 532 | 452 | 151 | N/S | aAt/aGt | tolerated(0.25) | benign(0.01) |
| rs150293032 | A | missense | NT5DC1 | ENSG00000178425 | ENST00000319550 | protein_coding | 987 | 905 | 302 | G/D | gGc/gAc | deleterious(0.03) | possibly_damaging(0.734) |
| rs201990931 | A | missense | NT5DC1 | ENSG00000178425 | ENST00000319550 | protein_coding | 332 | 250 | 84 | V/I | Gtt/Att | tolerated(0.69) | benign(0.051) |
|  | A | missense | NT5DC1 | ENSG00000178425 | ENST00000419791 | protein_coding | 330 | 250 | 84 | V/I | Gtt/Att | tolerated(0.76) | benign(0.122) |
| rs138072390 | T | missense | PSD | ENSG00000059915 | ENST00000020673 | protein_coding | 1039 | 512 | 171 | R/Q | cGg/cAg | tolerated_low_confidence(0.4) | benign(0) |
|  | T | missense | PSD | ENSG00000059915 | ENST00000406432 | protein_coding | 715 | 512 | 171 | R/Q | cGg/cAg | tolerated_low_confidence(0.4) | benign(0) |
| rs140739855 | T | missense | PSD | ENSG00000059915 | ENST00000611678 | protein_coding | 2540 | 1679 | 560 | R/Q | cGg/cAg | deleterious(0.01) | benign(0.033) |
|  | T | missense | PSD | ENSG00000059915 | ENST00000020673 | protein_coding | 3343 | 2816 | 939 | R/Q | cGg/cAg | deleterious(0.01) | benign(0.033) |
|  | T | missense | PSD | ENSG00000059915 | ENST00000406432 | protein_coding | 3019 | 2816 | 939 | R/Q | cGg/cAg | deleterious(0.01) | benign(0.033) |
| rs142273937 | A | missense | PSD | ENSG00000059915 | ENST00000611678 | protein_coding | 2602 | 1741 | 581 | R/W | Cgg/Tgg | deleterious(0.01) | probably_damaging(0.999) |
|  | A | missense | PSD | ENSG00000059915 | ENST00000020673 | protein_coding | 3405 | 2878 | 960 | R/W | Cgg/Tgg | deleterious(0) | probably_damaging(0.999) |
|  | A | missense | PSD | ENSG00000059915 | ENST00000406432 | protein_coding | 3081 | 2878 | 960 | R/W | Cgg/Tgg | deleterious(0) | probably_damaging(0.999) |
| rs144187898 | A | missense | PSD | ENSG00000059915 | ENST00000611678 | protein_coding | 1270 | 409 | 137 | L/F | Ctt/Ttt | tolerated(0.71) | benign(0.277) |
|  | A | missense | PSD | ENSG00000059915 | ENST00000020673 | protein_coding | 2073 | 1546 | 516 | L/F | Ctt/Ttt | tolerated(0.71) | benign(0.277) |
|  | A | missense | PSD | ENSG00000059915 | ENST00000406432 | protein_coding | 1749 | 1546 | 516 | L/F | Ctt/Ttt | tolerated(0.71) | benign(0.277) |
| rs145677127 | A | missense | PSD | ENSG00000059915 | ENST00000611678 | protein_coding | 1130 | 269 | 90 | G/V | gGt/gTt | tolerated(0.1) | probably_damaging(0.992) |
|  | A | missense | PSD | ENSG00000059915 | ENST00000020673 | protein_coding | 1933 | 1406 | 469 | G/V | gGt/gTt | tolerated(0.05) | probably_damaging(0.992) |
|  | A | missense | PSD | ENSG00000059915 | ENST00000406432 | protein_coding | 1609 | 1406 | 469 | G/V | gGt/gTt | tolerated(0.05) | probably_damaging(0.992) |
| rs146953868 | T | missense | PSD | ENSG00000059915 | ENST00000611678 | protein_coding | 1079 | 218 | 73 | R/Q | cGg/cAg | tolerated(1) | benign(0.001) |
|  | T | missense | PSD | ENSG00000059915 | ENST00000020673 | protein_coding | 1882 | 1355 | 452 | R/Q | cGg/cAg | tolerated(1) | benign(0.001) |
|  | T | missense | PSD | ENSG00000059915 | ENST00000406432 | protein_coding | 1558 | 1355 | 452 | R/Q | cGg/cAg | tolerated(1) | benign(0.001) |
| rs147203944 | T | missense | PSD | ENSG00000059915 | ENST00000611678 | protein_coding | 2786 | 1925 | 642 | R/Q | cGg/cAg | tolerated_low_confidence(0.09) | benign(0.001) |
|  | T | missense | PSD | ENSG00000059915 | ENST00000020673 | protein_coding | 3589 | 3062 | 1021 | R/Q | cGg/cAg | deleterious_low_confidence(0.01) | benign(0.001) |
|  | T | missense | PSD | ENSG00000059915 | ENST00000406432 | protein_coding | 3265 | 3062 | 1021 | R/Q | cGg/cAg | deleterious_low_confidence(0.01) | benign(0.001) |
| rs148732359 | T | missense | PSD | ENSG00000059915 | ENST00000611678 | protein_coding | 2603 | 1742 | 581 | R/Q | cGg/cAg | tolerated(0.49) | probably_damaging(0.994) |
|  | T | missense | PSD | ENSG00000059915 | ENST00000020673 | protein_coding | 3406 | 2879 | 960 | R/Q | cGg/cAg | tolerated(0.17) | probably_damaging(0.994) |
|  | T | missense | PSD | ENSG00000059915 | ENST00000406432 | protein_coding | 3082 | 2879 | 960 | R/Q | cGg/cAg | tolerated(0.17) | probably_damaging(0.994) |
| rs200141401 | G | missense | PSD | ENSG00000059915 | ENST00000611678 | protein_coding | 1181 | 320 | 107 | E/A | gAg/gCg | tolerated(0.68) | benign(0.017) |
|  | G | missense | PSD | ENSG00000059915 | ENST00000020673 | protein_coding | 1984 | 1457 | 486 | E/A | gAg/gCg | tolerated(0.57) | benign(0.017) |
|  | G | missense | PSD | ENSG00000059915 | ENST00000406432 | protein_coding | 1660 | 1457 | 486 | E/A | gAg/gCg | tolerated(0.57) | benign(0.017) |
| rs200819772 | A | missense | PSD | ENSG00000059915 | ENST00000611678 | protein_coding | 2488 | 1627 | 543 | R/W | Cgg/Tgg | deleterious(0) | possibly_damaging(0.887) |
|  | A | missense | PSD | ENSG00000059915 | ENST00000020673 | protein_coding | 3291 | 2764 | 922 | R/W | Cgg/Tgg | deleterious(0) | possibly_damaging(0.887) |
|  | A | missense | PSD | ENSG00000059915 | ENST00000406432 | protein_coding | 2967 | 2764 | 922 | R/W | Cgg/Tgg | deleterious(0) | possibly_damaging(0.887) |
| rs201641202 | G | missense | PSD | ENSG00000059915 | ENST00000020673 | protein_coding | 856 | 329 | 110 | S/T | aGt/aCt | deleterious_low_confidence(0.01) | benign(0) |
|  | G | missense | PSD | ENSG00000059915 | ENST00000406432 | protein_coding | 532 | 329 | 110 | S/T | aGt/aCt | deleterious_low_confidence(0.01) | benign(0) |
| rs145791657 | A | synonymous | PSD | ENSG00000059915 | ENST00000611678 | protein_coding | 1473 | 612 | 204 | N | aaC/aaT | NA | NA |
|  | A | synonymous | PSD | ENSG00000059915 | ENST00000020673 | protein_coding | 2276 | 1749 | 583 | N | aaC/aaT | NA | NA |
|  | A | synonymous | PSD | ENSG00000059915 | ENST00000406432 | protein_coding | 1952 | 1749 | 583 | N | aaC/aaT | NA | NA |
| rs114045527 | C | missense | SEC23IP | ENSG00000107651 | ENST00000369075 | protein_coding | 1984 | 1912 | 638 | D/H | Gac/Cac | tolerated(0.08) | benign(0.438) |
|  | A | missense | SEC23IP | ENSG00000107651 | ENST00000369075 | protein_coding | 2335 | 2263 | 755 | V/M | Gtg/Atg | tolerated(0.1) | benign(0.042) |
| rs142266445 | T | missense | SEC23IP | ENSG00000107651 | ENST00000543134 | protein_coding | 760 | 760 | 254 | V/F | Gtt/Ttt | deleterious(0.05) | possibly_damaging(0.681) |
|  | T | missense | SEC23IP | ENSG00000107651 | ENST00000442952 | protein_coding | 56 | 58 | 20 | V/F | Gtt/Ttt | tolerated(0.11) | possibly_damaging(0.578) |
|  | T | missense | SEC23IP | ENSG00000107651 | ENST00000369075 | protein_coding | 832 | 760 | 254 | V/F | Gtt/Ttt | tolerated(0.09) | benign(0.439) |
| rs142665854 | T | missense | SEC23IP | ENSG00000107651 | ENST00000543134 | protein_coding | 1241 | 1241 | 414 | T/M | aCg/aTg | deleterious(0) | probably_damaging(0.969) |
|  | T | missense | SEC23IP | ENSG00000107651 | ENST00000446561 | protein_coding | 353 | 353 | 118 | T/M | aCg/aTg | deleterious(0) | probably_damaging(0.927) |
|  | T | missense | SEC23IP | ENSG00000107651 | ENST00000369075 | protein_coding | 1313 | 1241 | 414 | T/M | aCg/aTg | deleterious(0) | probably_damaging(0.97) |
| rs145338788 | G | missense | SEC23IP | ENSG00000107651 | ENST00000369075 | protein_coding | 2449 | 2377 | 793 | I/V | Att/Gtt | tolerated(0.07) | benign(0.074) |
| rs73357833 | A | missense | SEC23IP | ENSG00000107651 | ENST00000543134 | protein_coding | 133 | 133 | 45 | A/T | Gct/Act | deleterious(0.01) | benign(0.231) |
|  | A | missense | SEC23IP | ENSG00000107651 | ENST00000369075 | protein_coding | 205 | 133 | 45 | A/T | Gct/Act | deleterious(0.02) | benign(0.079) |
| rs118110471 | A | missense | SEC23IP | ENSG00000107651 | ENST00000369075 | protein_coding | 2335 | 2263 | 755 | V/M | Gtg/Atg | tolerated(0.1) | benign(0.042) |
| rs151252286 | C | missense | ZCCHC4 | ENSG00000168228 | ENST00000505412 | protein_coding | 249 | 251 | 84 | I/T | aTt/aCt | deleterious(0.04) | benign(0.011) |
|  | C | missense | ZCCHC4 | ENSG00000168228 | ENST00000302874 | protein_coding | 680 | 656 | 219 | I/T | aTt/aCt | deleterious(0.05) | possibly_damaging(0.803) |
|  | C | missense | ZCCHC4 | ENSG00000168228 | ENST00000507760 | nonsense_mediated_decay | 671 | 656 | 219 | I/T | aTt/aCt | tolerated(0.06) | possibly_damaging(0.803) |
| rs186288207 | G | missense | ZCCHC4 | ENSG00000168228 | ENST00000612982 | protein_coding | 212 | 148 | 50 | N/D | Aat/Gat | tolerated(0.1) | possibly_damaging(0.708) |
|  | G | missense | ZCCHC4 | ENSG00000168228 | ENST00000302874 | protein_coding | 244 | 220 | 74 | N/D | Aat/Gat | tolerated(0.07) | benign(0.018) |
|  | G | missense | ZCCHC4 | ENSG00000168228 | ENST00000507760 | nonsense_mediated_decay | 235 | 220 | 74 | N/D | Aat/Gat | tolerated(0.06) | benign(0.018) |
| rs201763036 | C | missense | ZCCHC4 | ENSG00000168228 | ENST00000302874 | protein_coding | 71 | 47 | 16 | S/T | aGc/aCc | tolerated (0.27) | benign(0.005) |
|  | C | missense | ZCCHC4 | ENSG00000168228 | ENST00000507760 | nonsense_mediated_decay | 62 | 47 | 16 | S/T | aGc/aCc | tolerated (0.31) | benign(0.005) |
| rs3752873 | T | missense | ZCCHC4 | ENSG00000168228 | ENST00000505412 | protein_coding | 738 | 740 | 247 | P/L | cCg/cTg | tolerated(0.88) | benign(0) |
|  | T | missense | ZCCHC4 | ENSG00000168228 | ENST00000302874 | protein_coding | 1169 | 1145 | 382 | P/L | cCg/cTg | tolerated(1) | benign(0) |
| rs61746661 | G | missense | ZCCHC4 | ENSG00000168228 | ENST00000505412 | protein_coding | 315 | 317 | 106 | H/R | cAt/cGt | tolerated(0.99) | benign(0.011) |
|  | G | missense | ZCCHC4 | ENSG00000168228 | ENST00000302874 | protein_coding | 746 | 722 | 241 | H/R | cAt/cGt | tolerated(0.65) | benign(0.011) |
| rs79643299 | A | missense | ZCCHC4 | ENSG00000168228 | ENST00000302874 | protein_coding | 1450 | 1426 | 476 | Q/K | Caa/Aaa | tolerated(0.43) | benign(0.001) |
| rs192992705 | T | stop_gained | ZCCHC4 | ENSG00000168228 | ENST00000612982 | protein_coding | 278 | 214 | 72 | R/* | Cga/Tga | - | - |
|  | T | stop_gained | ZCCHC4 | ENSG00000168228 | ENST00000302874 | protein_coding | 310 | 286 | 96 | R/* | Cga/Tga | - | - |
|  | T | stop_gained | ZCCHC4 | ENSG00000168228 | ENST00000507760 | nonsense_mediated_decay | 301 | 286 | 96 | R/* | Cga/Tga | - | - |
| rs201478693 | T | stop_gained | ZCCHC4 | ENSG00000168228 | ENST00000612982 | protein_coding | 302 | 238 | 80 | R/* | Cga/Tga | - | - |
|  | T | stop_gained | ZCCHC4 | ENSG00000168228 | ENST00000302874 | protein_coding | 334 | 310 | 104 | R/* | Cga/Tga | - | - |
|  | T | stop_gained | ZCCHC4 | ENSG00000168228 | ENST00000507760 | nonsense_mediated_decay | 325 | 310 | 104 | R/* | Cga/Tga | - | - |
|  | A | synonymous | ZCCHC4 | ENSG00000168228 | ENST00000612982 | protein_coding | 302 | 238 | 80 | R | Cga/Aga | - | - |
|  | A | synonymous | ZCCHC4 | ENSG00000168228 | ENST00000302874 | protein_coding | 334 | 310 | 104 | R | Cga/Aga | - | - |
|  | A | synonymous | ZCCHC4 | ENSG00000168228 | ENST00000507760 | nonsense_mediated_decay | 325 | 310 | 104 | R | Cga/Aga | - | - |
| rs182505131 | T | stop_gained | ZCCHC4 | ENSG00000168228 | ENST00000505412 | protein_coding | 560 | 562 | 188 | R/* | Cga/Tga | - | - |
|  | T | stop_gained | ZCCHC4 | ENSG00000168228 | ENST00000302874 | protein_coding | 991 | 967 | 323 | R/* | Cga/Tga | - | - |
