# Supplementary material for: Exome chip analyses in adult attention deficit hyperactivity disorder
Source: Transl Psychiatry. 2016 Oct 18;6(10):e923–. doi: 10.1038/tp.2016.196 (PMC5315553; doi:10.1038/tp.2016.196)
Supplement: Supplementary Table 6 [file tp2016196x6.docx]

**Supplementary Table 6. Summary of functional analyses performed in RegulomeDB.**

“RegulomeDB binding score” reflects how likely is a SNV to affect the binding of a regulatory element at the locus where it resides. “Bound Protein” reflects proteins bound by ChIP (ENCODE data from both RegulomeDB).“Regulatory Motif” reflects ENCODE motif data from RegulomeDB. “Chromatine state” reflects the chromatine state annotation in brain tissue at the locus where the SNV resides as reported by RegulomeDB. “NA” stands for “non applicable”.

| **SNV** | **Gene^*^** | **RegulomeDB score** | **Bound Protein** | **Motif** | **Chromatin state** |
| --- | --- | --- | --- | --- | --- |
| rs147203944 | PSD | likely to affect binding (2b) | POLR2A | NERF1a | Transcription |
| rs148732359 | PSD | likely to affect binding (3a) | POLR2A | Nr2f2 and Rara | Transcription |
| rs142273937 | PSD | likely to affect binding (3a) | POLR2A | Nr2f2 and Rara | Transcription |
| rs140739855 | PSD | minimal biniding evidence(4) | over 20, including POLR2A and CTCF | NA | Transcription |
| rs200819772 | PSD | minimal biniding evidence(4) | over 20, including POLR2A and CTCF | NA | Transcription |
| rs145791657 | PSD | minimal biniding evidence(4) | POLR2A | NA | PolyComb and Transcription |
| rs144187898 | PSD | minimal biniding evidence(5) | NA | ZFBRK1 | Enhancer and transcription |
| rs200141401 | PSD | minimal biniding evidence(5) | NA | COUPTF | Enhancer and transcription |
| rs145677127 | PSD | minimal biniding evidence(5) | NA | GCM1 | Enhancer and transcription |
| rs146953868 | PSD | minimal biniding evidence(5) | NA | CP2/LBP-1c/LSF and HIC1 | Enhancer and transcription |
| rs138072390 | PSD | minimal biniding evidence(5) | NA | NA | Transcription |
| rs201641202 | PSD | minimal biniding evidence(5) | NA | NA | Transcription |
| rs73357833 | SEC23IP | minimal biniding evidence(4) | over 20, including POLR2A | NA | Active transcription start site |
| rs142266445 | SEC23IP | minimal biniding evidence(6) | NA | GR | Trancription |
| rs142665854 | SEC23IP | No Data | NA | NA | NA |
| rs114045527 | SEC23IP | minimal biniding evidence (5) | NA | 9 various motifs | Transcription |
| rs118110471 | SEC23IP | minimal biniding evidence (5) | NA | NA | Strong transcription |
| rs145338788 | SEC23IP | No Data | NA | NA | NA |
| rs201990931 | NT5DC1 | No Data | NA | NA | NA |
| rs150257749 | NT5DC1 | minimal biniding evidence(6) | NA | Sox2 and SOX9 | Weak transcription |
| rs146980198 | COL10A1 | minimal biniding evidence(5) | NA | NA | Weak transcription |
| rs151327195 | COL10A1 | minimal biniding evidence(5) | NA | AP-2alpha and gamma, TFAP2A | Weak transcription |
| rs150293032 | NT5DC1 | No Data | NA | NA | NA |
| rs201763036 | ZCCHC4 | minimal binding evidence (4) | over 20, including POLR2A and CTCF | NA | Active transcription start site |
| rs186288207 | ZCCHC4 | minimal biniding evidence(6) | NA | Tcfap2e | Weak transcription |
| rs192992705 | ZCCHC4 | minimal biniding evidence(6) | NA | ATF3 | Transcription |
| rs201478693 | ZCCHC4 | minimal biniding evidence(6) | NA | ESR1 | Transcription |
| rs151252286 | ZCCHC4 | No Data | NA | NA | NA |
| rs61746661 | ZCCHC4 | minimal biniding evidence(6) | NA | Sox30, Sry | Weak transcription |
| rs182505131 | ZCCHC4 | No Data | NA | NA | NA |
| rs3752873 | ZCCHC4 | No Data | NA | NA | NA |
| rs79643299 | ZCCHC4 | minimal biniding evidence(5) | NA | Cdx | Weak transcription |

**^*^** only those genes are reported where the variants are protein coding
