# Supplementary material for: Exome chip analyses in adult attention deficit hyperactivity disorder
Source: Transl Psychiatry. 2016 Oct 18;6(10):e923–. doi: 10.1038/tp.2016.196 (PMC5315553; doi:10.1038/tp.2016.196)
Supplement: Supplementary Table 7 [file tp2016196x7.docx]

**Supplementary Table 7. The top GO terms (p≤1.00E-03) observed in meta-analysis of both common and rare variants**.

“NGENES” refers to the average number of genes per go term present in our dataset. Bonferroni corrected significance threshold for pathway analysis of GO terms was set to p = 2.17E-05.

| **GO term** | **Annotation** | **NGENES** | **P-value** |
| --- | --- | --- | --- |
| GO:0031124 | mRNA 3'-end processing | 22 | 0.000107 |
| GO:0000118 | histone deacetylase complex | 21 | 0.000187 |
| GO:0006369 | termination of RNA polymerase II transcription | 28 | 0.000259 |
| GO:0030521 | androgen receptor signaling pathway | 32 | 0.000642 |
| GO:0043087 | regulation of GTPase activity | 12 | 0.000764 |
