# Supplementary material for: Exome chip analyses in adult attention deficit hyperactivity disorder
Source: Transl Psychiatry. 2016 Oct 18;6(10):e923–. doi: 10.1038/tp.2016.196 (PMC5315553; doi:10.1038/tp.2016.196)

Supplementary Figure 1. QQ plots of single point association tests of each dataset individually as well as meta-analysis of common variants (MAF≥1%).

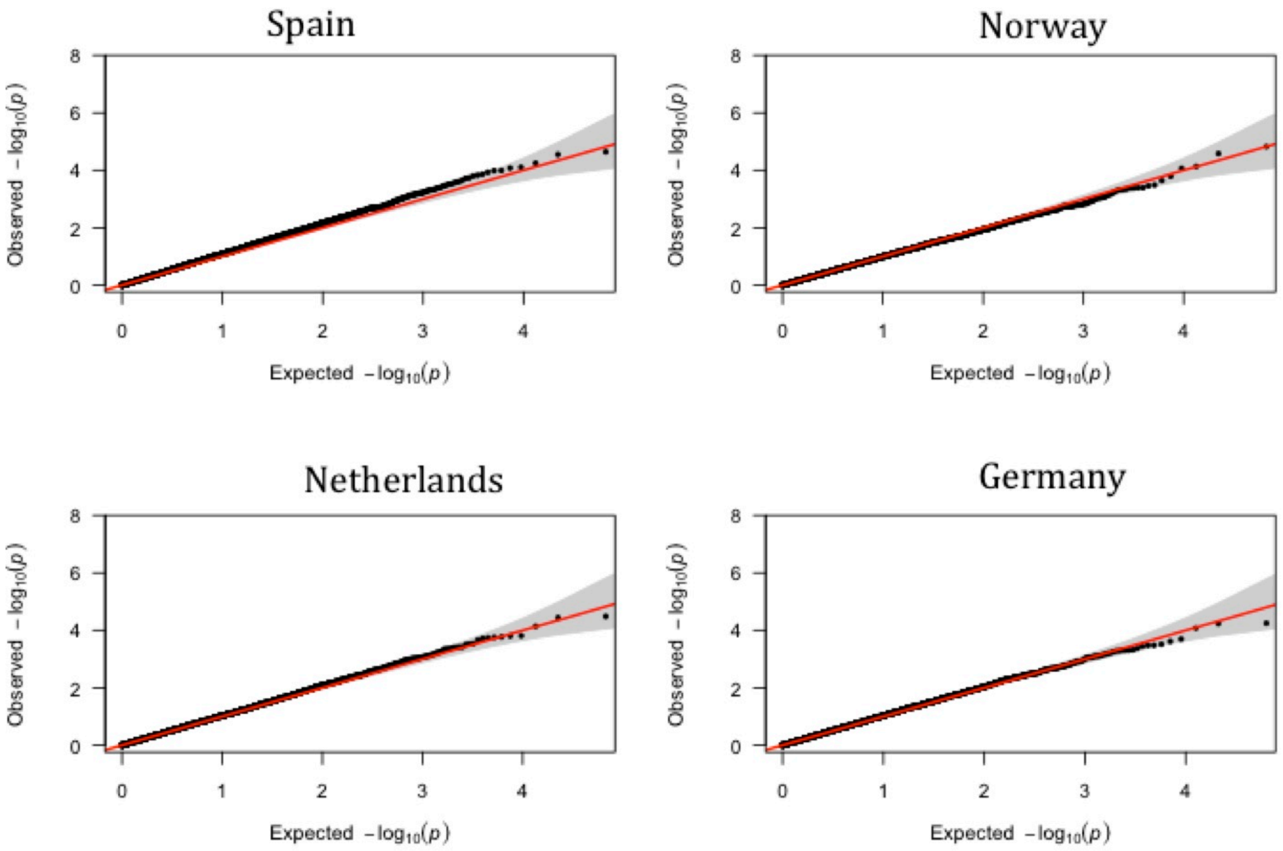

Inverse variance fixed effects meta-analysis in METAL

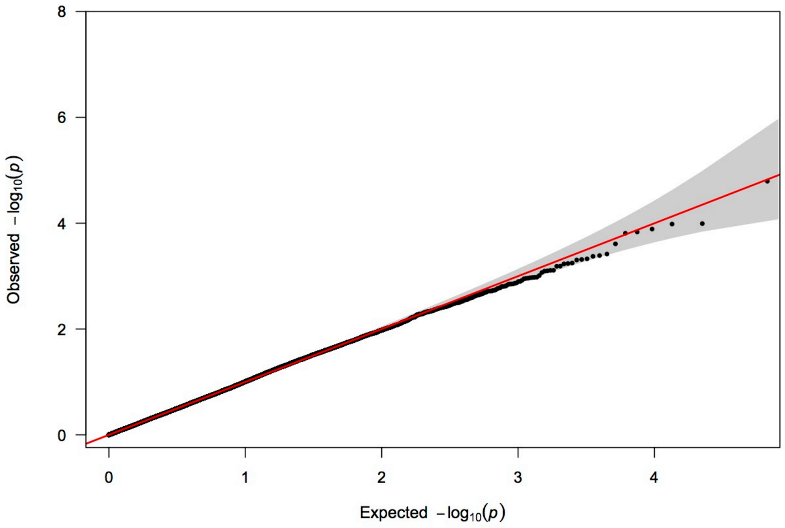

Supplement: Supplementary Figure 1 [file tp2016196x8.pdf]
