# Supplementary material for: Exome chip analyses in adult attention deficit hyperactivity disorder
Source: Transl Psychiatry. 2016 Oct 18;6(10):e923–. doi: 10.1038/tp.2016.196 (PMC5315553; doi:10.1038/tp.2016.196)

Supplementary Figure 2. Forest plots reflecting the overall meta-analysis (discovery and replication datasets together) for the most significant top three common SNVs observed in this study.

A) rs9325032 (*PPP2R2B* gene)

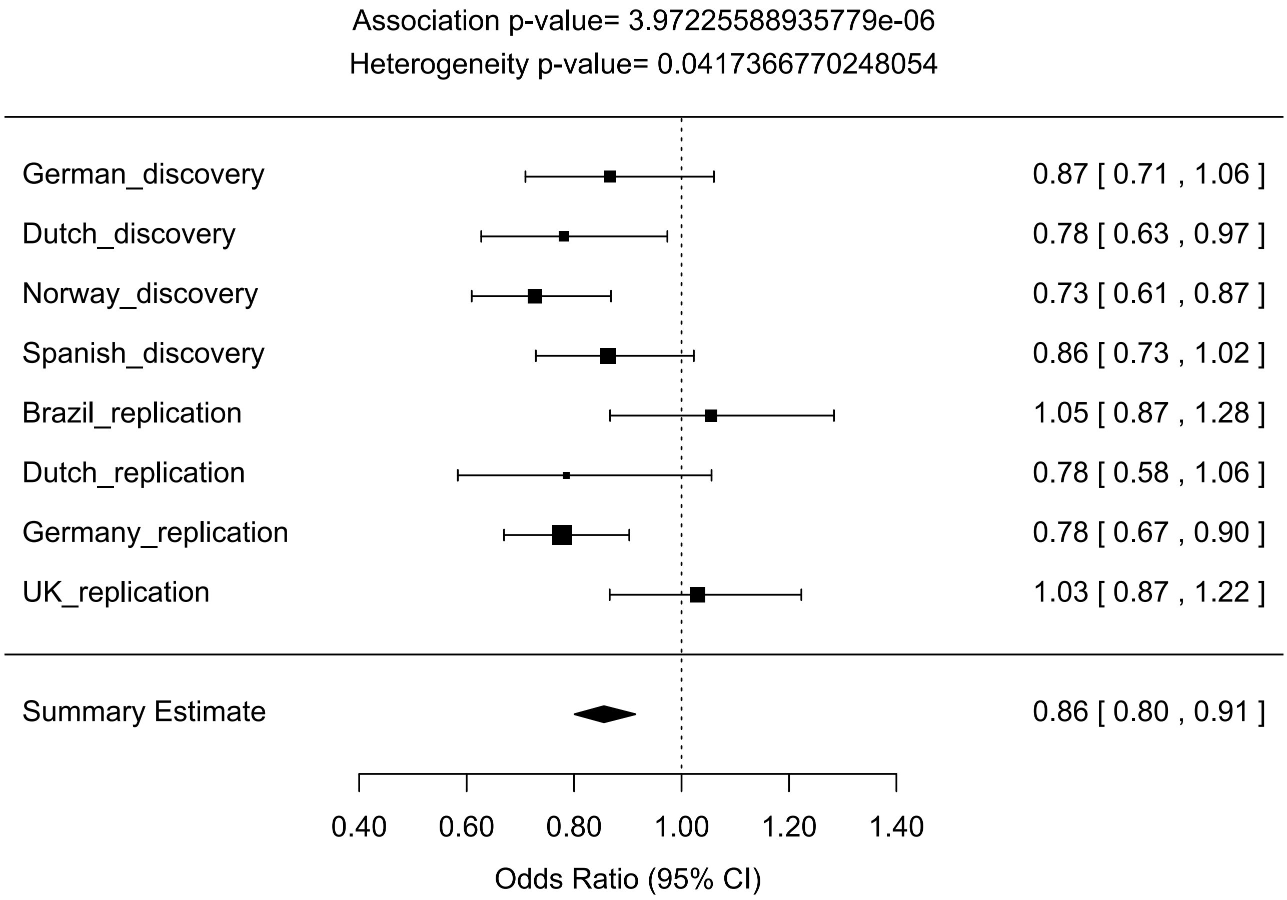

B) rs3095150 (intergenic)

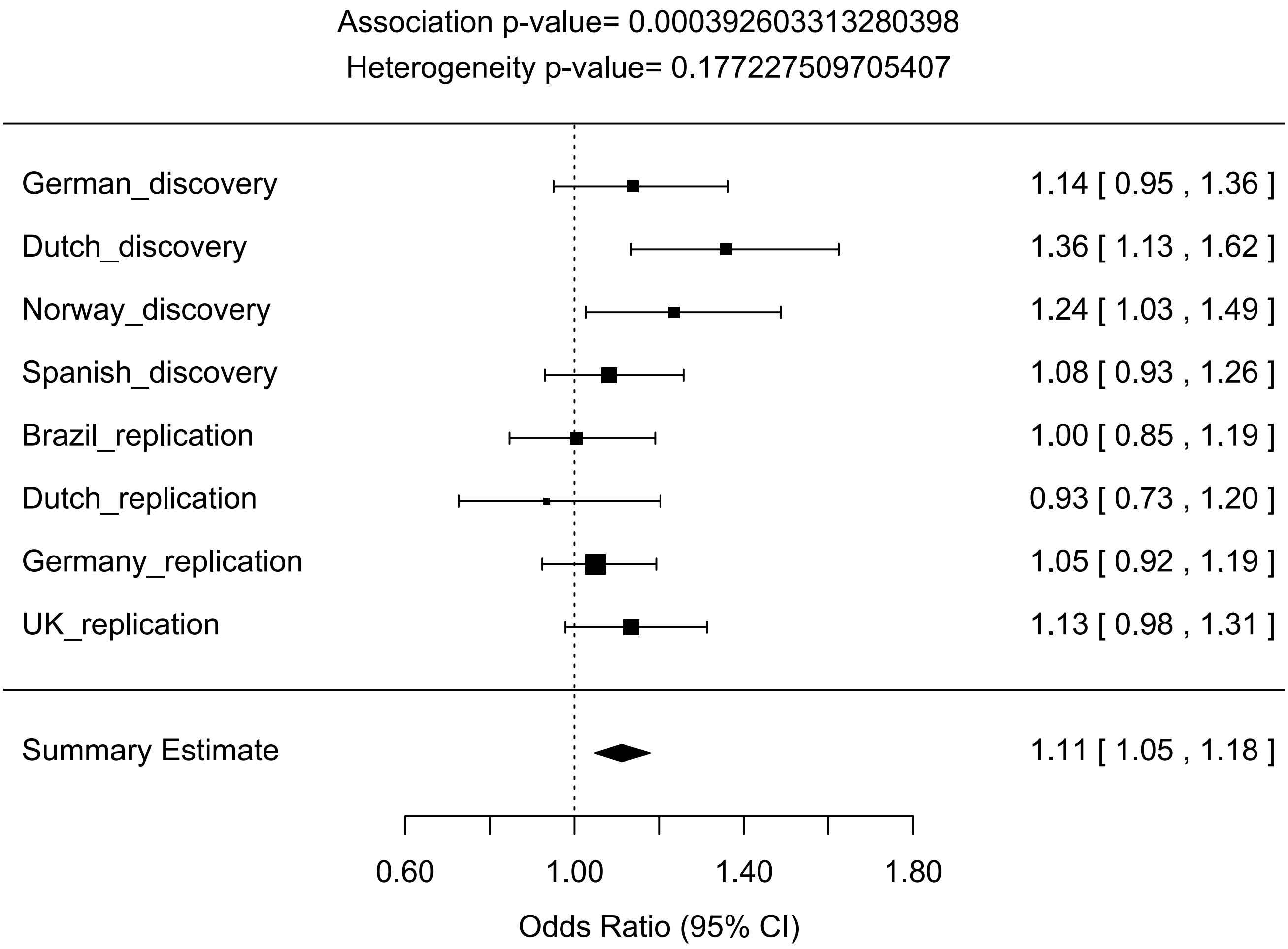

C) rs117611723 (*SYNRG* gene)

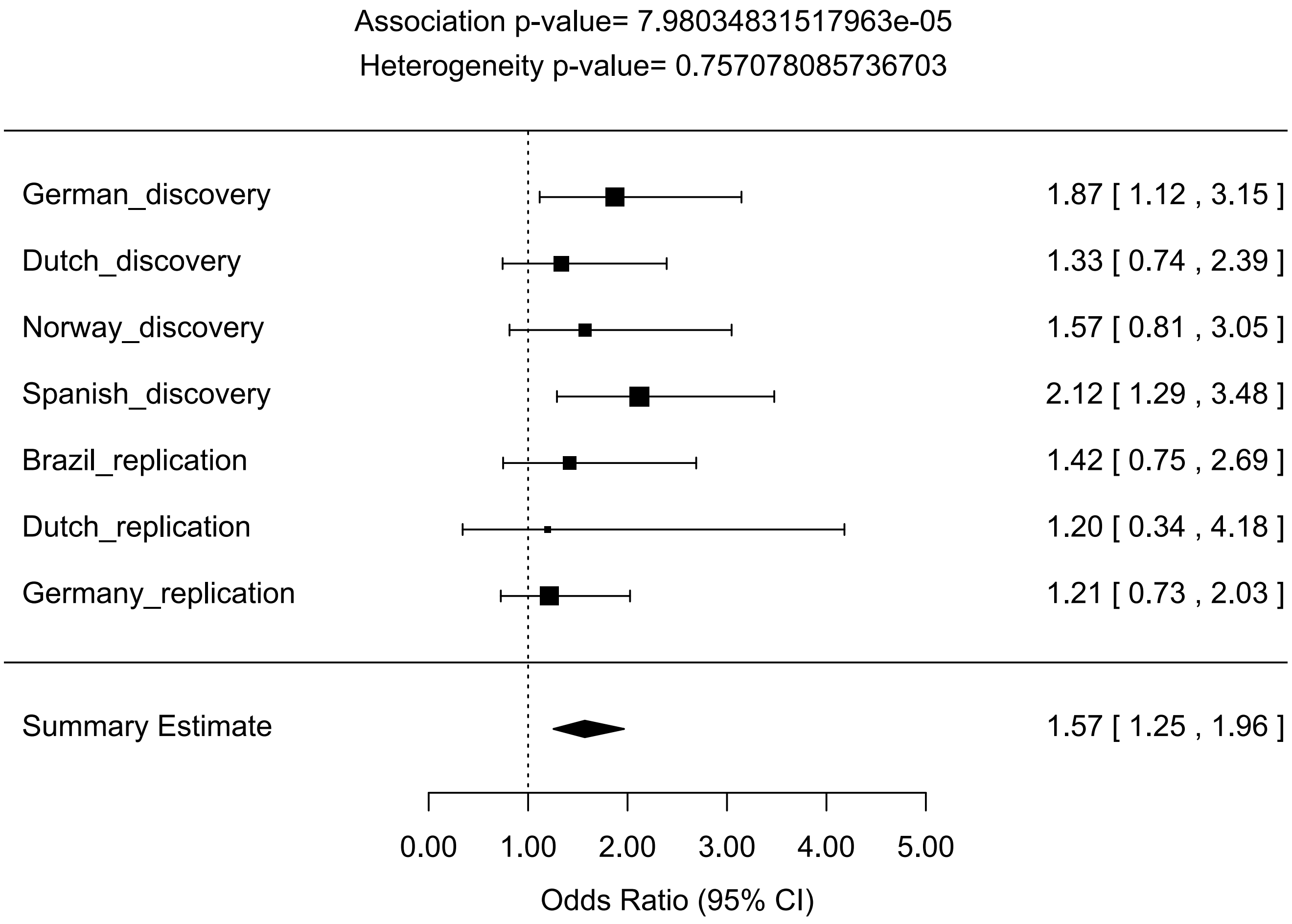

Supplement: Supplementary Figure 2 [file tp2016196x9.pdf]
