# Supplementary material for: Exome chip analyses in adult attention deficit hyperactivity disorder
Source: Transl Psychiatry. 2016 Oct 18;6(10):e923–. doi: 10.1038/tp.2016.196 (PMC5315553; doi:10.1038/tp.2016.196)
Supplement: Supplementary Figure 3 [file tp2016196x10.pdf]

Supplementary Figure 3. Homology models and Structural inspection of missense mutation sites in human NT5DC1.

A. *Homology models of human NT5DC1.*
Models were generated automatically from the human protein sequence (NP_689942.2) using SWISS-MODEL [1, 2](red), Raptor [3](light blue), Phyre2 [4](green), M4T [5](yellow) and IntFOLD2 [6](dark blue). The homology models were based on pdb structures of; 4ohf, 2bde, 4g63, 2jc9, 2j2c, 4h4b, 2xjc, 2xje, 2xcv, 2xjb, 2xjd, 2xcw, 2xjf, 2jcm, 2xcx, 4ygq, 4ygr, 4ygs (SWISS-MODEL); 2bde and 2xje (Raptor); 2jm, 3i28, 2pke, 3ib6, 2pr7, 2om6, 1cr6, 3iru, 3ddh, 3um9, 3s6j, and 3d6j (Phyre2); 4ohf and 2xcw (M4T); 2j2c, 2bde, 4xaq, 4gpa (IntFOLD2, global quality score 0.7813).

B. *Structural inspection of missense mutation sites in hNT5DC1.*
Using homology models of hNT5DC1 the structural details surrounding the sites of missense mutations were investigated in Discovery studio Visualizer (v 4.1). The protein backbone is shown with secondary structure (colored according to the pKa value), the mutated amino acids are show as ball and stick, whereas other relevant amino acids are shown with color codes; grey (hydrophobic/ aromatic), red (basic), blue (acidic) and green (threonine). The Swiss homology model was used for illustration. Panel (B1) shows amino acids asparagine 81 (N81) (turn between two beta-sheet strands) and valine 84 (V84) (end of a beta-sheet strand, hydrophobic cluster). Panel (B2) shows asparagine 151 (N151) with plausible hydrogen-boding to lysine 154 (K154). Lysine 148 (K148), shown as red, may attract phosphorylation at serine 151 in the N151S mutant. Panel (B3) shows G302 located in a loop, with neighboring basic amino acids (K298, K299) and a potential phosphorylation site (T301) that may induce conformational change of the loop similar to that of G302D.

References

1. Biasini M, Bienert S, Waterhouse A, Arnold K, Studer G, Schmidt T *et al.* SWISS-MODEL: modelling protein tertiary and quaternary structure using evolutionary information. *Nucleic Acids Res* 2014; **42**(Web Server issue)**:** W252-258.
2. Arnold K, Bordoli L, Kopp J, Schwede T. The SWISS-MODEL workspace: a web-based environment for protein structure homology modelling. *Bioinformatics* 2006; **22**(2)**:** 195-201.
3. Xu J, Li M, Kim D, Xu Y. RAPTOR: optimal protein threading by linear programming. *J Bioinform Comput Biol* 2003; **1**(1)**:** 95-117.
4. Kelley LA, Mezulis S, Yates CM, Wass MN, Sternberg MJ. The Phyre2 web portal for protein modeling, prediction and analysis. *Nat Protoc* 2015; **10**(6)**:** 845-858.
5. Fernandez-Fuentes N, Madrid-Aliste CJ, Rai BK, Fajardo JE, Fiser A. M4T: a comparative protein structure modeling server. *Nucleic Acids Res* 2007; **35**(Web Server issue)**:** W363-368.
6. McGuffin LJ, Atkins JD, Salehe BR, Shuid AN, Roche DB. IntFOLD: an integrated server for modelling protein structures and functions from amino acid sequences. *Nucleic Acids Res* 2015; **43**(W1)**:** W169-173.

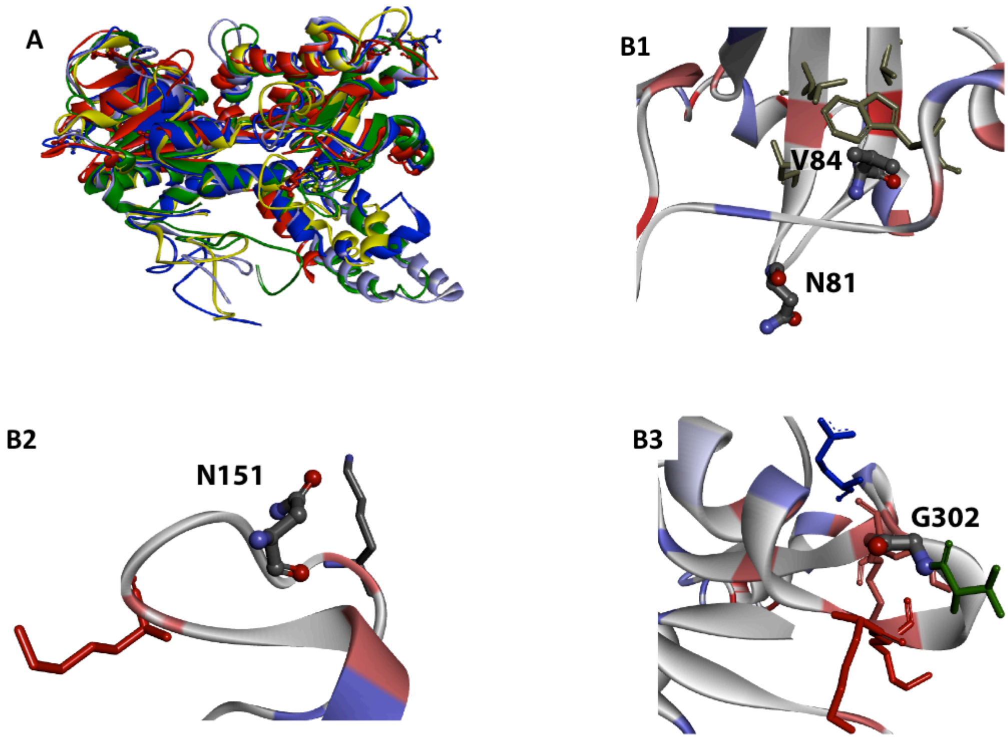
A
B1
V84
N81
B2
N151
B3
G302
