# Supplementary material for: Exome chip analyses in adult attention deficit hyperactivity disorder
Source: Transl Psychiatry. 2016 Oct 18;6(10):e923–. doi: 10.1038/tp.2016.196 (PMC5315553; doi:10.1038/tp.2016.196)

```
                                                         :: * .:                         * : : .
lcl|Query_37229  MAQHFSLAACDVVGFDLDHTLCRYNLPESAPLIYNSFAQFLVKEKGYDKELLNVTPEDWDFCCKGLALDLEDGNFLKLAN   80
gi|114608985     MAQHFSLAACDVVGFDLDHTLCRYNLPESAPLIYNSFAQFLVKEKGYNKELLNVTPEDWDFCCKGLALDLEDGNFLKLAN   80
gi|426354317     MAQHFSLAACDVVGFDLDHTLCRYNLPESAPLIYNSFAQFLVKEKGYDKELLNVTPEDWDFCCKGLALDLEDGNFLKLAN   80
gi|795513538     MAQHFSLAACDVVGFDLDHTLCRYNLPESAPLIYNSFAQFLVKEKGYDKELLNVTPEDWDFCCKGLALDLEDGNFLKLAD   80
gi|387763464     MAQHFSLAACDVVGFDLDHTLCRYNLPESAPLIYNSFAQFLVKEKGYDKELLSVTPEDWDFCCKGLALDLEDGNFLKLAD   80
gi|795549508     MAQHFSLAACDVVGFDLDHTLCRYNLPESAPLIYNSFAQFLVKEKGYDKELLSVTPEDWDFCCKGLALDLEDGNFLKLAD   80
gi|795513542     MAQHFSLAACDVVGFDLDHTLCRYNLPESAPLIYNSFAQFLVKEKGYDKELLNVTPEDWDFCCKGLALDLEDGNFLKLAD   80
gi|544424790     MAQHFSLAACDVVGFDLDHTLCRYNLPESAPLIYNSFAQFLVKEKGYDKELLSVTPEDWDFCCKGLALDLEDGNFLKLAD   80
gi|724807254     MAQQFSLAACDVVGFDLDHTLCRYNLPESAPLIYNSFAQFLVKEKGYDKELLSVTPEDWDFCCKGLALDLEDGNFLKLAD   80
gi|795130302     MAQQFSLAACDVVGFDLDHTLCRYNLPESAPLIYNSFAQFLVKEKGYDKELLSVTPEDWDFCCKGLALDLEDGNFLKLAD   80
gi|817291021     MAQHFSLAACDVVGFDLDHTLCRYNLPESAPLIYNSFAQFLVKEKGYDKELLNVTPEDWDFCCKGLALDLEDGNFLKLAD   80
gi|296199057     MAQHFSLAACDVVGFDLDHTLCRYNLPESAPLIYNSFAQFLVKEKGYDKELLNVTPEDWDFCCKGLALDLEDGNFLKLAD   80
gi|724807257     MAQQFSLAACDVVGFDLDHTLCRYNLPESAPLIYNSFAQFLVKEKGYDKELLSVTPEDWDFCCKGLALDLEDGNFLKLAD   80
gi|403295527     MAQHFSLAACDVVGFDLDHTLCRYNLPESAPLIYNSFAQFLVKEKGYDKELLSVTPEDWDFCCKGLALDLEDGNFLKLAD   80
gi|817291023     MAQHFSLAACDVVGFDLDHTLCRYNLPESAPLIYNSFAQFLVKEKGYDKELLNVTPEDWDFCCKGLALDLEDGNFLKLAD   80
gi|675654011     MAQHFSLAACDVVGFDLDHTLCRYNLPESAPLIYNSFAQFLVKEKGYDKELLNVTPEDWDFCCKGLALDLEDGNFLKLAD   80
gi|725594041     MAQHFSLAACDVVGFDLDHTLCRYNLPESAPLIYNSFAQFLVKEKGYDKELLSVTPEDWDFCCKGLALDLEDGNFLKLAD   80
gi|829988711     MAQHFSLAACDVVGFDLDHTLCRYNLPESAPLIYNSFAQFLVKERGYDKELLSVTPEDWDFCCKGLALDLEDGNFIKLAD   80
gi|395816322     MAQHFSLAACDVVGFDLDHTLCRYNLPESAPLIYNSFAQFLVKERGYDKELLNVTPEDWDFCCKGLALDLEDGNFIKLAD   80
gi|826310252     MAQHFSLAACDVVGFDLDHTLCRYNLPESAPLIYNSFAQFLVKERGYDKELLSVTPEDWDFCCKGLALDLEDGNFIKLAD   80
gi|410959884     MAQHFSLAACDVVGFDLDHTLCRYNLPESAVLIYNSFAQFLVKEKGYDKELLTVTPEDWDFCCKGLALDLEDGNFIKLAD   80
gi|585164297     MAQHFSLAACDVVGFDLDHTLCRYNLPESAVLIYNSFAQFLVKEKGYDKELLTVTPEDWDFCCKGLALDLEDGNFIKLAD   80
gi|586987268     MAQHFSLAACDVVGFDLDHTLCRYNLPESAVLIYNSFAQFLVKEKGYDKELLTVTPEDWDFCCKGLALDLEDGNFIKLAD   80
gi|472356764     MAQHFSLAACDVVGFDLDHTLCRYNLPESAVLIYNSFAQFLVKEKGYDKELLTVTPEDWDFCCKGLALDLEDGNFIKLAD   80
gi|602685616     MAQHFSLAACDVVGFDLDHTLCRYNLPESARLIYNSFAQFLVKEKGYDKELLTVTPEDWDFCCKGLALDLEDGNFIKLAD   80
gi|667279819     MAQHFSLAACDVVGFDLDHTLCRYNLPESARLIYNSFAQFLVKEKGYNKELLTVTSEDWDFCCKGLALDMEDGNFIKLAD   80
gi|667279822     MAQHFSLAACDVVGFDLDHTLCRYNLPESARLIYNSFAQFLVKEKGYNKELLTVTSEDWDFCCKGLALDMEDGNFIKLAD   80
gi|465979262     MAQHFSLAACDVVGFDLDHTLCRYNLPESARLIYNSFAQFLVKEKGYDKELLTVTPEDWDFCCKGLALDLEDGNFIKLAD   80
gi|194035204     MAQHFSLAACDVVGFDLDHTLCRYNLPESARLIYNSFAQFLVREKGYDKELLTVTPEDWDFCCKGLALDLEDGNFIKLAD   80
gi|759133407     MAQHFSLASCDVVGFDLDHTLCRYNLPESAPLIYNSFAQFLVKEKGYGKELLTVTPEDWDFCCKGLALDLEDGNFIKLAD   80
gi|641734335     MAQHFSLAACDVVGFDLDHTLCRYNLPESAPLIYHSFAQFLVKEKGYDKELLTVTPEDWDFCCKGLALDLEDGNFIKLAD   80
gi|511930642     MAQHFSLAACDVVGFDLDHTLCRYNLPESALLIYNSFAQFLVKEKGYDKELLTVTPEDWDFCCKGLALDLEDGNFIKLAD   80
gi|744561719     MAQHFSLAACDVVGFDLDHTLCRYNLPESARLIYNSFAEFLVKEKGYDKELLTVTPEDWDFCCKGLALDLEDGNFIKLAD   80
gi|558121862     MAQHFSLAACDVVGFDLDHTLCRYNLPESAPLIYDSFAQFLVKEKGYDKELLTVTPEDWDFCCKGLALDLEDGNFIKLAD   80
gi|478495911     MAQHFSLVNCDVVGFDLDHTLCRYNLPESAALIYNSFAQFLVKERGYDKELLTVAPEDWDFCCKGLALDLEGGNFIKLAD   80
gi|556739024     MAQHFSLAACDVVGFDLDHTLCCYNLPESARLIYNSFAQFLVKEKGYDKGLLTVTPEDWDFCCKGLALDLEDGNFIKLAD   80
gi|655843044     MAQHFSLAACDVVGFDLDHTLCRYNLPESAPLIYNCFAQFLVKERGYHKELLTVTPEDWDFCCKGLALDLEDGTFIKLAD   80
gi|507925770     MAQHFSLAACDVVGFDLDHTLCRYNLPESARLIYNSFAQFLVKEKGYDKELLTVTPEDWDFCCKGLALDLEDGSFIKLAD   80
gi|829980860     MAQHFSLAACDVVGFDLDHTLCRYNLPESARLIYNSFAQFLVKEKGYDKELLTVTPEDWDFCCKGLALDLEDGSFIKLAD   80
gi|426234499     MAQHFSLAACDVVGFDLDHTLCCYNLPESARLIYNSFAQFLVKEKGYDKGLLTVTPEDWDFCCKGLALDLEDGNFIKLAD   80
gi|157428094     MAQHFSLAACDVVGFDLDHTLCCYNLPESARLIYNSFAQFLVKEKGYDKGLLTVTPEDWDFCCKGLALDLEDGNFIKLAD   80
gi|594063404     MAQHFSLAACDVVGFDLDHTLCCYNLPESARLIYNSFAQFLVKEKGYDKGLLTVTPEDWDFCCKGLALDLEDGNFIKLAD   80
gi|803097535     MAQHFSLAACDVVGFDLDHTLCCYNLPESARLIYNSFAQFLVKEKGYDKGLLTVTPEDWDFCCKGLALDLEDGNFIKLAD   80
gi|670997190     MGQ-------------------------VAQLIYNSFAQFLVKEKGYDKELLTVTPEDWDFCCKGLALDLEDGNFIKLAD   55
gi|548479506     MAQHFSLAACDVVGFDLDHTLCCYNLPESARLIYNSFAQFLVKEKGYDKGLLTVTPEDWDFCCKGLALDLEDGNFIKLAD   80
gi|555991352     MAQHFSLAACDVVGFDLDHTLCCYNLPESARLIYNSFAQFLVKEKGYDKGLLTVTPEDWDFCCKGLALDLEDGNFIKLAD   80
gi|528960483     MAQHFSLAACDVVGFDLDHTLCCYNLPESARLIYNSFAQFLVKEKGYDKGLLTVTPEDWDFCCKGLALDLEDGNFIKLAD   80
gi|594063406     MAQHFSLAACDVVGFDLDHTLCCYNLPESARLIYNSFAQFLVKEKGYDKGLLTVTPEDWDFCCKGLALDLEDGNFIKLAD   80
gi|548479508     MAQHFSLAACDVVGFDLDHTLCCYNLPESARLIYNSFAQFLVKEKGYDKGLLTVTPEDWDFCCKGLALDLEDGNFIKLAD   80
gi|664701045     M------------------------------LIYNSFAQFLVKEKGYDKELLTVTPEDWDFCSKGLALDLEDGHFIKLAD   50
gi|820999445     MAQHFSLAACDVVGFDLDHTLCRYNLPESAPLIYNSFAQFLVKEKGYDKELLTVTPEDWDFCCKGLALDLEDGTFIKLAD   80
gi|554586740     -----------MTGQEVRDSLIRL----TGKLIYDSFAQFLVKEKGYDKELLTVTPEDWDFCCKGLALDLEDGNFIKLAD   65
gi|584073388     -----------MTGQEVRDSLIRL----TGKLIYDSFAQFLVKEKGYDKELLTVTPEDWDFCCKGLALDLEDGNFIKLAD   65
gi|558127235     -----------MTGQEVRDSLIRL----TGKLIYDSFAQFLVKEKGYDKELLTVTPEDWDFCCKGLALDLEDGNFIKLAD   65
gi|512920735     MAQHFSLAACDVVGFDLDHTLCRYNLPESAPLIYNSFARFLVKEKGYDKELLTVTPEDWDFCCKGLALDLEDGTFIKLAE   80
gi|617593341     MAQHFSLASCDVVGFDLDHTLCRYNLPESAPLIYNSFAEFLVKEKGYDKELLTVTPEDWDFCCKGLALDLEDGSFIKLAD   80
gi|674085287     MAQYFSLATCDVVGFDLDHTLCRYNLPESARLIYNSFAQFLVKERGYDKELLTLTSEDWDFCCKGLALDLEDGTFIKLAD   80
gi|820979643     ----------------------------------------MIEEGAFK------------------------GTINEQCA   16
gi|625280199     MAQHFSLAACDVVGFDLDHTLCRYNLPESARLIYNSFAQFLVKEKGYDKELLTLTPEDWDFCCKGLALDLEDGTFIKLAA   80
gi|532041477     MAQHFSLAACDVVGFDLDHTLCRYNLPESARLIYTSFAQFLVQEKGYDKELLTLTPEDWDFCCKGLALDLEDGTFIKLAA   80
gi|625280201     MAQHFSLAACDVVGFDLDHTLCRYNLPESARLIYNSFAQFLVKEKGYDKELLTLTPEDWDFCCKGLALDLEDGTFIKLAA   80
gi|672086605     MAQHFSLAACDVVGFDLDHTLCRYNLSESAALIYNSFAQFLVKEKGYDEELLTLTLEDWDFCCKGLALDLEDGTFIKLAA   80
gi|110626010     MAQHFSLGACDVVGFDLDHTLCRYNLPESARLIYNSFAQFLVKEKGYDEGLLTLTPEDWDFCCKGLALDLEDGTFIKLAA   80
gi|524945211     MAQHFSLAACDVVGFDLDHTLCRYNLPESARLIYTSFAQFLVKEKGYDKELLTLTPEDWDFCCKGLALDLEDGTFIKLAA   80
gi|568965457     MAQHFSLGACDVVGFDLDHTLCRYNLPESARLIYNSFAQFLVKEKGYDEGLLTLTPEDWDFCCKGLALDLEDGTFIKLAA   80
gi|672086607     MAQHFSLAACDVVGFDLDHTLCRYNLSESAALIYNSFAQFLVKEKGYDEELLTLTLEDWDFCCKGLALDLEDGTFIKLAA   80
gi|675654013     MAQHFSLAACDVVGFDLDHTLCRYNLPESAPLIYNSFAQFLVKEKGYDKELLNVTPEDWDFCCKGLALDLEDGNFLKLAD   80
gi|530639256     MTRPFSLAECDALGFDLDHTLCRYNRPETARLIYDSFARYLVTEKGYDKELLTVTPESWDFCCKGLALDLEDGNFLKLAG   80
gi|543344792     MQQHFSLAECDVVGFDLDHTLCRYHLPQSARLIYDSFAQYLVTEKGYDEDLLTLAPDSLDFCCKGLVLDIEEGNFLKLAE   80
gi|542164235     MQQHFSLAECDVVGFDLDHTLCRYHLPQSARLIYDSFAQYLVTEKGYDEDLLTLAPDSLDFCCKGLVLDIEEGNFLKLAE   80
gi|363732051     MQQYFSLADCEVMGFDLDHTLCRYHLPQSAALIYDSFAQYLVTEKGYDKDLLTLNPESLDFCCKGLALDLEDGNFLKLAE   80
gi|683891145     MQQHFSLAECDVVGFDLDHTLCRYHLPQSTRLIYDSFAQYLVTEKGYDEDLLTLAPDCLDFCCKGLVLDIEEGNFLKLAE   80
gi|543344789     MQQHFSLAECDVVGFDLDHTLCRYHLPQSARLIYDSFAQYLVTEKGYDEDLLTLAPDSLDFCCKGLVLDIEEGNFLKLAE   80
gi|449497791     MQQHFSLAECDVVGFDLDHTLCRYHLPQSARLIYDSFAQYLVTEKGYDEDLLTLAPDSLDFCCKGLVLDIEEGNFLKLAE   80
gi|542164233     MQQHFSLAECDVVGFDLDHTLCRYHLPQSARLIYDSFAQYLVTEKGYDEDLLTLAPDSLDFCCKGLVLDIEEGNFLKLAE   80
gi|50744564      MQQYFSLADCEVMGFDLDHTLCRYHLPQSAALIYDSFAQYLVTEKGYDKDLLTLNPESLDFCCKGLALDLEDGNFLKLAE   80
gi|729750329     MQQHFSLADCDVMGFDLDHTLCRYHLPQSARLIYDSFAQYLVTEKGYDKDLLTLAPDSLDFCCKGLVLDIEEGDFLKLAE   80
gi|768365056     MQQYFSLADCDVMGFDLDHTLCRYHLPQSARLIYDSFAQYLVTEKGYDKDLLTLAPDSLDFCCKGLVLDIEEGDFLKLAE   80
                 1.......10........20........30........40........50........60........70........80
```

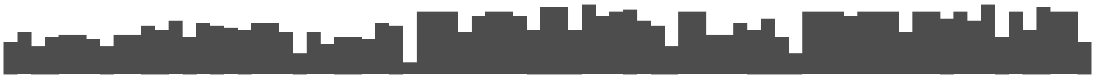

```
                  ..  :  ::  .  .   :                                .* * **************::.* *
lcl|Query_37229   NGTVLRASHGTKMMTPEVLAEAYGKKEWKHFLSD----------TGMACRSGKYYFYDNYFDLPGALLCARVVDYL-TKL   149
gi|114608985      NGTVLRASHGTKMMTPEVLAEAYGKKEWKHFLSD----------TGMACRSGKYYFYDNYFDLPGALLCARVVDYL-TKL   149
gi|426354317      NGTVLRASHGTKMMTPEVLAEAYGKKEWKHFLSD----------TGMACRSGKYYFYDNYFDLPGALLCARVVDYL-TKL   149
gi|795513538      NGTVLRASHGTKMMAPEVLAEAYGKKEWKHFLSD----------TGMACRSGKYYFYDNYFDLPGALLCARVVDYL-TKQ   149
gi|387763464      NGTVLRASHGTKMMAPEVLAEAYGKKEWKHFLSD----------TGMACRSGKYYFYDNYFDLPGALLCARVVDYL-TKQ   149
gi|795549508      NGTVLRASHGTKMMAPEVLAEAYGKKEWKHFLSD----------TGMACRSGKYYFYDNYFDLPGALLCARVVDYL-TQQ   149
gi|795513542      NGTVLRASHGTKMMAPEVLAEAYGKKEWKHFLSD----------TGMACRSGKYYFYDNYFDLPGALLCARVVDYL-TK-   148
gi|544424790      NGTVLRASHGTKMMAPEVLAEAYGKKEWKHFLSD----------TGMACRSGKYYFYDNYFDLPGALLCARVVDYL-TK-   148
gi|724807254      NGTVLRASHGTKMMAPEVLAEAYGKKEWKHFLSD----------TGMACRSGKYYFYDNYFDLPGALLCARVVDYL-TKQ   149
gi|795130302      NGTVLRASHGTKMMAPEVLAEAYGKKEWKHFLSD----------TGMACRSGKYYFYDNYFDLPGALLCARVVDYL-TKQ   149
gi|817291021      NGTVLRASHGTKMMTPAVVAEAYGKKEWKHFLSD----------TGMACRSGKYYFYDNYFDLPGALLCARVVDYL-TKQ   149
gi|296199057      NGTVLRASHGTKMMTPAVLAEAYGKKEWKHFLSD----------TGMACRSGKYYFYDNYFDLPGALLCARVVDYL-TKQ   149
gi|724807257      NGTVLRASHGTKMMAPEVLAEAYGKKEWKHFLSD----------TGMACRSGKYYFYDNYFDLPGALLCARVVDYL-TK-   148
gi|403295527      NGTVLRASHGTKMMTPAVLAEAYGKKEWKHFLSD----------TGMACRSGKYYFYDNYFDLPGALLCARVVDYL-TKQ   149
gi|817291023      NGTVLRASHGTKMMTPAVVAEAYGKKEWKHFLSD----------TGMACRSGKYYFYDNYFDLPGALLCARVVDYL-TK-   148
gi|675654011      NGTVLRASHGTKMMTPAVLAEAYGKKEWKHFLSD----------TGMACRSGKYYFYDNYFDLPGALLCARVVDYL-TK-   148
gi|725594041      NGTVLRASHGTKMMTPAVLAEAYGKKEWKHFLSD----------TGMACRSGKYYFYDNYFDLPGALLCARVVDYL-TK-   148
gi|829988711      NGTVLRASHGTKMMTPEALAEAYGKEEWKHFVSD----------TGMTCRSGKYYFYDNYFDLPGALLCARVVDLL-TKH   149
gi|395816322      NGTVLRASHGTKMMTAEALSAAYGKEEWKHFVSD----------TGMVCRSGKYYFYDNYFDLPGALLCARVVDLL-TKQ   149
gi|826310252      NGTVLRASHGTKMMTPEALAEAYGKEEWKHFVSD----------TGMTCRSGKYYFYDNYFDLPGALLCARVVDLL-TKH   149
gi|410959884      NGTVLRASHGTKMMAPEALAEEYGGKEWKYFMSD----------TGMACRSGKYYFYDNYFDLPGALLCARVVDSL-TKQ   149
gi|585164297      NGTVLRASHGTNMMAPEALVEEYGRKEWKHFMSD----------AGMACRSGKYYFYDNYFDLPGALLCARVVDSL-TKQ   149
gi|586987268      NGTVLRASHGTKMMAPEALAEEYGGKEWKYFMSD----------TGMACRSGKYYFYDNYFDLPGALLCARVVDSL-TK-   148
gi|472356764      NGTVLRASHGTKMMAPEALVEEYGRKEWKHFMSD----------AGMACRSGKYYFYDNYFDLPGALLCARVVDSL-TKQ   149
gi|602685616      NGTVLRASHGTKMLAPEVLAEEYGRKEWKHFTPD----------SGMACRSGKYYFYDNYFDLPGALLCARVVDSL-TKQ   149
gi|667279819      NGTVLRASHGTKMMTPEALAEVYGKKEWKHFMSD----------TGMPCHSGKYYFYDNYFDLPGALLCARVVDSL-TKQ   149
gi|667279822      NGTVLRASHGTKMMTPEALAEVYGKKEWKHFMSD----------TGMPCHSGKYYFYDNYFDLPGALLCARVVDSL-TK-   148
gi|465979262      NGTVLRASHGTKMLAPEVLAEEYGRKEWKHFTPD----------SGMACRSGKYYFYDNYFDLPGALLCARVVDSL-TKQ   149
gi|194035204      NGTVLRASHGTKMLTPEALEEEYGQKEWKHFLSD----------SGTACRSGKYYFYDNYFDLPGALLCARVVDSL-TKQ   149
gi|759133407      NGTVLRASHGTKMMAPEELAEEYGRKKWKYFMSD----------TGMACRSGKYYFYDNYFDLPGALLCARVVDSL-TKA   149
gi|641734335      NGTVLRASHGTRMLAPEELAKEYGRREWKYFMSD----------AGMALRSGKYYFYDNYFDLPGALLCARVVDSL-TKQ   149
gi|511930642      NGTVLRASHGTKMMAPEALAEEYGGKEWKHFMSD----------AGMACRSGKYYFYDNYFDLPGALLCARVVDSL-SKR   149
gi|744561719      NGSVLRASHGTRMMAPEALAEEYGRREWRHFPAE----------AAVACRSGKYYFYDNYFDLPGALLCARVVDSL-TK-   148
gi|558121862      NGTVLRASHGTRMLAPEELAKEYGGKEWKYFMSD----------TGMAFRSGKYYFYDNYFDLPGALLCARVVDSL-TKQ   149
gi|478495911      NGTVLRASHGTKMMAPEALAEEYGGKEWKHFTPD----------TGWSCRSGKCYFYDNYFDLPGALLCARVVDSL-TKQ   149
gi|556739024      NGTVLRASHGTKMLSPEALAEEFGRKEWKHFMPD----------TGMAFRSGKYYFYDNYFDLPGALLCARVVDSL-TKQ   149
gi|655843044      DGTVLRASHGTKMMTPEALAEAYGKKEWKHFVSD----------TGMPCRSGKYYFYDNYFDLPGALLCARVVDSL-TK-   148
gi|507925770      DGTVLRASHGTKMVAAQALAEEYGSKAWKHFMMD----------TETASRSGKYYFYDNYFDLPGALLCARVVDFL-TKK   149
gi|829980860      DGTVLRASHGTKMVAAQALAEEYGSKAWKHFMMD----------TETASRSGKYYFYDNYFDLPGALLCARVVDFL-TK-   148
gi|426234499      NGTVLRASHGTKMLSPEALAEEFGRKEWKHFMPD----------TGMAFRSGKYYFYDNYFDLPGALLCARVVDSL-TKQ   149
gi|157428094      NGTVLRASHGTKMLSAEALAEEFGRKEWKHFMPD----------TGMAFRSGKYYFYDNYFDLPGALLCARVVDSL-TKQ   149
gi|594063404      NGTVLRASHGTKMLSAEALAEEFGRKQWKHFMPD----------TGMAFRSGKYYFYDNYFDLPGALLCARVVDSL-TKQ   149
gi|803097535      NGTVLRASHGTKMLSPEALAEEFGRKEWKHFMPD----------TGMAFRSGKYYFYDNYFDLPGALLCARVVDSL-TK-   148
gi|670997190      NGTVLRASHGTKMMPPEALAAAYGRKEWKHFMSD----------TGMACRSGKYYFYDNYFDLPGALLCARVVDSL-TKQ   124
gi|548479506      NGTVLRASHGTKMLSPEALAAEFGRKEWKHFMPD----------TGMAFRSGKYYFYDNYFDLPGALLCARVVDSL-TKQ   149
gi|555991352      NGTVLRASHGTKMLSAEALAEEFGRKEWKHFMPD----------TGMAFRSGKYYFYDNYFDLPGALLCARVVDSL-TKQ   149
gi|528960483      NGTVLRASHGTKMLSAEALAEEFGRKEWKHFMPD----------TGMAFRSGKYYFYDNYFDLPGALLCARVVDSL-TK-   148
gi|594063406      NGTVLRASHGTKMLSAEALAEEFGRKQWKHFMPD----------TGMAFRSGKYYFYDNYFDLPGALLCARVVDSL-TK-   148
gi|548479508      NGTVLRASHGTKMLSPEALAAEFGRKEWKHFMPD----------TGMAFRSGKYYFYDNYFDLPGALLCARVVDSL-TK-   148
gi|664701045      NGTVLRASHGTKMMAPEVLAEQYGRKEWKHFISD----------TGMASRSGKYYFYDNYFDLPGALLCARVVDSL-TKQ   119
gi|820999445      NGTVLRASRGTKMMAPDVLAKEYGAKEWKYFVSD----------TGMPSHPGKYYFYDNYFDLPGALLCARVVDSL-TKQ   149
gi|554586740      NGTVLRASHGTRMLAPEELAKEYGGREWKYFMSD----------TGMAFRSGKYYFYDNYFDLPGALLCARVVDSL-TKQ   134
gi|584073388      NGTVLRASHGTRMLAPEELAKEYGGREWKYFMSD----------SGMAFRSGKYYFYDNYFDLPGALLCARVVDSL-TKQ   134
gi|558127235      NGTVLRASHGTRMLAPEELAKEYGGREWKYFMSD----------TGMAFRSGKYYFYDNYFDLPGALLCARVVDSL-TKQ   134
gi|512920735      NGTVLRASHGTKMMTPDMLAEAYGKKEWKYFVSD----------TGMPSHPGKYYFYDNYFDLPGALLCARVADSL-TKQ   149
gi|617593341      NGTVLSRSRWRRCSAQMTL--------------------------------GKYYFYDNYFDLPGALLCARVVDSL-TKK   127
gi|674085287      DGTVLRASHGTKMMTPEALAKAYGKKEWKHFVSD----------TDMPCCSGKCYFYDNYFDLPGALLCARVVDSL-TK-   148
gi|820979643      SSLKIRASHGTKMMTPEVLAEAYGKKEWKHFLSD----------TGMACRSGKYYFYDNYFDLPGALLCARVVDYL-TKQ    85
gi|625280199      DGTVLRASHGTRMMTPEELVETYGKKDWRHCTTDRHCAANV----DIPCCSGKCYFYDNYFDLPGALLCARVVDSL-TK-   154
gi|532041477      DGTVLRASHGTRMMTPEALAETYGKKDWRHCVTDKHCASSV----DIPCCSGKCYFYDNYFDLPGALLCARVVDSL-TK-   154
gi|625280201      DGTVLRASHGTRMMTPEELVETYGKKDWRHCTTDRHCAANV----DIPCCSGKCYFYDNYFDLPGALLCARVVDSL-TK-   154
gi|672086605      DGTVLRASHGTKMMTPEALTEAYGKKEWRHCLSDKRSVSG----SDIPCCSGKCYFYDNYFDLPGALLCAKVVDSL-TK-   154
gi|110626010      DGTVLRASHGTKMMTPEALTEAFGKKEWRHCVSDKRCTSDKPGVSDIPCCSGKCYFYDNYFDLPGALLCARVVDSL-TK-   158
gi|524945211      DGTVLRASHGTRMMTPEELVETYGKKDWRHCTTDRHCTSNI----DVPCCSGKCYFYDNYFDLPGALLCARVVDSL-TK-   154
gi|568965457      DGTVLRASHGTKMMTPEALTEAFGKKEWRHCVSDKRCTSDKPGVSDIPCCSGKCYFYDNYFDLPGALLCARVVDSL-TK-   158
gi|672086607      DGTVLRASHGTKMMTPEALTEAYGKKEWRHCLSDKRSVSG----SDIPCCSGKCYFYDNYFDLPGALLCAKVVDSL-TK-   154
gi|675654013      NGTVLRASHGTKMMTPAVLAEAYGKKEWKHFLSD----------TGMACRSGKYYFYDNYFDLPGALLCARVVDYL-TKQ   149
gi|530639256      DGTILRASHGTKSMTPEEILEIYGRREWKHFKTF----------SGMVSRSAKYYCYDNYFDLPGALLCARIVDCL-DK-   148
gi|543344792      DGTVLRASHGTKSMTSEEILEIYGRKEWKHFNTV----------SGMVSRTAKYYLYDNYFDLPGALLCARVVDSL-DQL   149
gi|542164235      DGTVLRASHGTKSMTSEEVLEVYGRKEWKHFNTV----------SGMVSRTAKYYLYDNYFDLPGALLCARVVDSL-DQL   149
gi|363732051      DGTVLRASHGTRSMTSEEILEIYGRREWKHFSTV----------SGMLSRSAKYYLYDNYFDLPGALLCARVVDKLLKM-   149
gi|683891145      DGTVLRASHGTKSMTSEEVLEIYGRKEWKHFNTV----------SGMVSRTAKYYLYDNYFDLPGALLCARVVDSL-DQL   149
gi|543344789      DGTVLRASHGTKSMTSEEILEIYGRKEWKHFNTV----------SGMVSRTAKYYLYDNYFDLPGALLCARVVDSL-DQ-   148
gi|449497791      DGTVLRASHGTKSMTFEEILEIYGRKEWKHFNTV----------SGMVSRTAKYYLYDNYFDLPGALLCARVVDSL-DQ-   148
gi|542164233      DGTVLRASHGTKSMTSEEVLEVYGRKEWKHFNTV----------SGMVSRTAKYYLYDNYFDLPGALLCARVVDSL-DQ-   148
gi|50744564       DGTVLRASHGTRSMTSEEILEIYGRREWKHFSTV----------SGMLSRSAKYYLYDNYFDLPGALLCARVVDSL-DQ-   148
gi|729750329      DGTVLRATHGTKSMTSEEILETYGRREWKHFNTV----------SGMVSRSAKYYLYDNYFDLPGALLCARVVDSL-DQ-   148
gi|768365056      DGTVLRASHGTKSMTSEEILETYGRREWKHFNTV----------SGMVSRSAKYYLYDNYFDLPGALLCARVVDSL-DQ-   148
                  ........90.......100.......110.......120.......130.......140.......150.......160
```

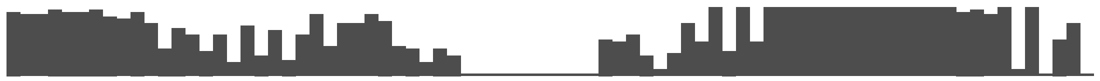

```
                            ::****: *.**:*:: ***.*:.* :***:*..  :*:  **.**:.**:::*.: *: :**********::
lcl|Query_37229     NN-GQKTFDFWKDIVAAIQHNYKMSAFKENCGIYFPEIKRDPGRYLHSCPESVKKWLRQLKNAGKILLLITSSHSDYCRL   228
gi|114608985        NN-GQKTFDFWKDIVAAIQHNYKMSAFKENCGIYFPEIKRDPGRYLHSCPESVKKWLRQLKNAGKILLLITSSHSDYCRL   228
gi|426354317        NN-GQKTFDFWKDIVAAIQHNYKMSAFKENCGIYFPEIKRDPGRYLHSCPESVKKWLRQLKNAGKILLLITSSHSDYCRL   228
gi|795513538        NN-GQKTFDFWKDIVAAIQHNYKMSAFKENCGIYFPEIKRDPGRYLHSCPESVKKWLRQLKNAGKILLLITSSHSDYCRL   228
gi|387763464        NN-GQKTFDFWKDIVAAIQHNYKMSAFKENCGIYFPEIKRDPGRYLHSCPESVKKWLRQLKNAGKILLLITSSHSDYCRL   228
gi|795549508        NN-GQKTFDFWKDIVAAIQHNYKMSAFKENCGIYFPEIKRDPGRYLHSCPESVKKWLRQLKNAGKILLLITSSHSDYCRL   228
gi|795513542        NN-GQKTFDFWKDIVAAIQHNYKMSAFKENCGIYFPEIKRDPGRYLHSCPESVKKWLRQLKNAGKILLLITSSHSDYCRL   227
gi|544424790        NN-GQKTFDFWKDIVAAIQHNYKMSAFKENCGIYFPEIKRDPGRYLHSCPESVKKWLRQLKNAGKILLLITSSHSDYCRL   227
gi|724807254        NS-GQKTFDFWKDIVAAIQHNYKMSAFKENCGIYFPEIKRDPGRYLHSCPESVKKWLRQLKNAGKILLLITSSHSDYCRL   228
gi|795130302        NN-GQKTFDFWKDIVAAIQHNYKMSAFKENCGIYFPEIKRDPGRYLHSCPESVKKWLRQLKNAGKILLLITSSHSDYCRL   228
gi|817291021        NN-GQKTFDFWKDIVAAIQHNYKMSAFKENCGIYFPEIKRDPGRYLHSCPESVKKWLRQLKNAGKILLLITSSHSDYCRL   228
gi|296199057        NN-GQKTFDFWKDIVAAIQHNYKMSAFKENCGIYFPEIKRDPGRYLHSCPESVKKWLRQLKNAGKILLLITSSHSDYCRL   228
gi|724807257        NS-GQKTFDFWKDIVAAIQHNYKMSAFKENCGIYFPEIKRDPGRYLHSCPESVKKWLRQLKNAGKILLLITSSHSDYCRL   227
gi|403295527        NN-GQKTFDFWKDIVAAIQHNYKMSAFKENCGIYFPEIKRDPGRYLHSCPESVKKWLRRLKNAGKILLLITSSHSDYCRL   228
gi|817291023        NN-GQKTFDFWKDIVAAIQHNYKMSAFKENCGIYFPEIKRDPGRYLHSCPESVKKWLRQLKNAGKILLLITSSHSDYCRL   227
gi|675654011        NN-GQKTFDFWKDIVAAIQHNYKMSAFKENCGIYFPEIKRDPGRYLHSCPESVKKWLRQLKNAGKILLLITSSHSDYCRL   227
gi|725594041        NN-GQKTFDFWKDIVAAIQHNYKMSAFKENCGIYFPEIKRDPGRYLHSCPESVKKWLRRLKNAGKILLLITSSHSDYCRL   227
gi|829988711        NN-GQKTFDFWKDIIAGIQHNYKTSAFKENSGIYFPEIKRDPGRYLHSCPESVKKWLRQLKNAGKILLLITSSHSDYCRL   228
gi|395816322        NN-GQKTFDFWKDIVAGIQHNYKMSAFKENCGMYFPEIKRDPGRYLHRCPESVKKWLHQLKNAGKILLLITSSHSDYCRL   228
gi|826310252        NN-GQKTFDFWKDIIAGIQHNYKTSAFKENSGMYFPEIKRDPGRYLHSCPESVKKWLRQLKNAGKILLLITSSHSDYCRL   228
gi|410959884        NN-GQKTFDFWKDIVAGIQHNYKMSAFKENCGIYFPEIKRDPGRYLHTCPESVKKWLRQLKNAGKILLLITSSHSDYCRL   228
gi|585164297        NN-GQKTFDFWKDIVAGIQHNYKMSAFKENCGIYFPEIKRDPGRYLHTCPDSVKKWLQQLKNAGKILLLITSSHSDYCRL   228
gi|586987268        NN-GQKTFDFWKDIVAGIQHNYKMSAFKENCGIYFPEIKRDPGRYLHTCPESVKKWLRQLKNAGKILLLITSSHSDYCRL   227
gi|472356764        NN-FFFSFDFWKDIVAGIQHNYKMSAFKENCGIYFPEIKRDPGRYLHTCPESVKKWLQQLKNAGKILLLITSSHSDYCRL   228
gi|602685616        NS-GQKPFDFWKDIVAGIQHNYKMSAFKENCGIYFPEIKRDPGRYLHTCPESVKKWLRQLKNAGKILMLITSSHSDYCRL   228
gi|667279819        N--GQKTFDFWKDIVAAIQHNYKMSAFNENCGIYFPEIKRDPSKYLHSCPESVKKWLRRLKKAGKILLLITSSHSDYCRF   227
gi|667279822        N--GQKTFDFWKDIVAAIQHNYKMSAFNENCGIYFPEIKRDPSKYLHSCPESVKKWLRRLKKAGKILLLITSSHSDYCRF   226
gi|465979262        NN-GQKPFDFWKDIVAGIQHNYKMSAFKENCGIYFPEIKRDPGRYLHTCPESVKKWLRQLKNAGKILMLITSSHSDYCRL   228
gi|194035204        NS-GQKPFDFWKDIVAGIQHNYKMSAFKENCGIYFPEIKRDPGRYLHTCPESVKKWLRQLKNAGKILMLITSSHSDYCRL   228
gi|759133407        NN-GQKTFDFWKDIVASIQHNYKVSAFKENCGMYFPEIKRDPGRYLYRCPESVKKWLRQLKNAGKILLLITSSHSDYCRL   228
gi|641734335        NN-GQKTFDFWKDIVAGIQHNYKMSAFKENCGIYFPEIKRDPGKYLHSCPESVKKWLRQLKNAGKILVLITSSHSDYCRL   228
gi|511930642        NN-GQKTFDFWKDIVAGIQHNYKMSAFKENCGIYFPEIKRDPGRYLHTCPESVKKWLQQLKNAGKILLLITSSHSDYCRL   228
gi|744561719        HN-GQKPFDFWKDIVAGIQYNYKMSAFKENCGIYFPEIKRDPGRYLRTCPESVKKWLQQLKNAGKILMLITSSHSDYCRL   227
gi|558121862        NN-GQKTFDFWKDIVAGIQHNYKMSAFRENCGIYFPEIKRDPGKYLHSCPKSVKKWLRQLKNARKILVLITSSHSDYCRL   228
gi|478495911        NN-GQKTFDFWKDIAAGIQHNYRMSAFKENCGIYFPEVKRDPGRYLHSCPESVKKWLRRLKNAGKILLLITSSHSDYCRL   228
gi|556739024        NS-GQKPFDFWKDIVAGIQHNYKMSAFKENCGMYFPEIKRDPGKYLHTCPESVKKWLRQLKNAGKILMLITSSHSDYCRL   228
gi|655843044        HNSGQKTFDFWKDIVAGIQHNFKMSAFKENCGLYFPEIKRDPGRYLHSCPESVRKWLRQLKNAGKILLLITSSHSDYCRL   228
gi|507925770        NN-GQKAFDFWKDVIAAIQHNYKMSAFKENCGIYFPEIKRNPGKYVHSCPESVKKWLRQLKSSGKILLLITSSHSDYCRL   228
gi|829980860        NN-GQKAFDFWKDVIAAIQHNYKMSAFKENCGIYFPEIKRNPGKYVHSCPESVKKWLRQLKSSGKILLLITSSHSDYCRL   227
gi|426234499        NN-GQKPFDFWKDIVAGIQHNYKMSAFKENCGIYFPEIKRDPGKYLHTCPESVKKWLRQLKNAGKILMLITSSHSDYCRL   228
gi|157428094        NN-GQKPFDFWKDIVAGIQHNYKMSAFKENCGIYFPEIKRDPGKYLHTCPESVKKWLRQLKNAGKILMLITSSHSDYCRL   228
gi|594063404        NN-GQKPFDFWKDIVAGIQHNYKMSAFKENCGIYFPEIKRDPGKYLHTCPESVKKWLRQLKNAGKILMLITSSHSDYCRL   228
gi|803097535        NN-GQKPFDFWKDIVAGIQHNYKMSAFKENCGIYFPEIKRDPGKYLHTCPESVKKWLRQLKNAGKILMLITSSHSDYCRL   227
gi|670997190        NN-GQKTFDFWKDIVAGIQHNYKMSAFKENCGIYFPEIKRDPGRYLHTCPESVKKWLQQLKNAGKILLLITSSHSDYCRL   203
gi|548479506        NN-SQKPFDFWKDIVAGIQHNYKMSAFKENCGIYFPEIKRDPGKYLHTCPESVKKWLRQLKNAGKILMLITSSHSDYCRL   228
gi|555991352        NN-GQKPFDFWKDIVAGIQHNYKMSAFKENCGIYFPEIKRDPGKYLHTCPESVKKWLRQLKNAGKILMLITSSHSDYCRL   228
gi|528960483        NN-GQKPFDFWKDIVAGIQHNYKMSAFKENCGIYFPEIKRDPGKYLHTCPESVKKWLRQLKNAGKILMLITSSHSDYCRL   227
gi|594063406        NN-GQKPFDFWKDIVAGIQHNYKMSAFKENCGIYFPEIKRDPGKYLHTCPESVKKWLRQLKNAGKILMLITSSHSDYCRL   227
gi|548479508        NN-SQKPFDFWKDIVAGIQHNYKMSAFKENCGIYFPEIKRDPGKYLHTCPESVKKWLRQLKNAGKILMLITSSHSDYCRL   227
gi|664701045        NN-GQKTFDFWKDVVAAIQHNYKVSAFKENCGIYFPEVKRDPGRYLYSCPESVKKWLQQLKNAGKILLLITSSHSDYCRL   198
gi|820999445        NS-GQKTFDFWKDIVAGIQHNFKMSAFKENCGLYFPEIKRNPGKYLHSCPESVRKWLQQLKNAGKILLLITSSHSDYCRL   228
gi|554586740        NN-GQKTFDFWKDIVAGIQHNYKMSAFRENCGIYFPEIKRDPGKYLHSCPESVKKWLRQLKNAGKILVLITSSHSDYCRL   213
gi|584073388        NN-GQKTFDFWKDIVAGIQHNYKMSAFRENCGIYFPEIKRDPGKYLHSCPESVKKWLRQLKNAGKILVLITSSHSDYCRL   213
gi|558127235        NN-GQKTFDFWKDIVAGIQHNYKMSAFRENCGIYFPEIKRDPGKYLHSCPESVKKWLQQLKNAGKILVLITSSHSDYCRL   213
gi|512920735        NS-GQKTFDFWKDIVAGIQHNFKMSAFKENCGLYFPEIKRDPGKYLHSCPESVRNWLQQLKSAGKILLLITSSHSDYCKL   228
gi|617593341        SG--QKTFDFWKDIVAAIQHNYKMSAFKENCGIYFPEIKRDPGKYLNRCPESVKKWLQQLKKAGKVLLLITSSHSDYCRL   205
gi|674085287        QNRGQKTFDFWKDVVAGIQHNFKMSAFKENCGLFFPEIKRNPGKYVYRCPESVRKWLRQLKNAGKIVVLITSSHSDYCKL   228
gi|820979643        NN-GQKTFDFWKDIVAAIQHNYKMSAFKENCGIYFPEIKRDPGRYLHSCPESVKKWLRQLKNAGKILLLITSSHSDYCRL   164
gi|625280199        QNRGQKTFDFWKDVVAGIQHNFKMSAFKENCGFFFPEIKRNLGKYVHRCPESVKRWLRQLKDAGKITMLITSSHSDYCKL   234
gi|532041477        QNRGQKTFDFWKDVVAGIQHNFKMSAFKENCGFFFPEIKRNLGKYVYRCPESVRRWLRQLKDAGKITMLITSSHSDYCKL   234
gi|625280201        -NRGQKTFDFWKDVVAGIQHNFKMSAFKENCGFFFPEIKRNLGKYVHRCPESVKRWLRQLKDAGKITMLITSSHSDYCKL   233
gi|672086605        QNRGQKTFDFWKDVVAGIQHNFKMSAFKENCGFFFPEIKRNLGKYVYRCPESVKKWLQQIKDAGKITMLITSSHSDYCKL   234
gi|110626010        QNRGQKTFDFWKDVVAGIQHNFKMSAFKENCGFFFPEIKRNLGKYVHRCPESVRKWLRQLKDAGKITMLITSSHSDYCKL   238
gi|524945211        QNRGQKTFDFWKDVVAGIQHNFKMSAFKENCGFFFPEIKRNLGKYVHRCPESVKRWLRQLKDAGKITMLITSSHSDYCKL   234
gi|568965457        -NRGQKTFDFWKDVVAGIQHNFKMSAFKENCGFFFPEIKRNLGKYVHRCPESVRKWLRQLKDAGKITMLITSSHSDYCKL   237
gi|672086607        -NRGQKTFDFWKDVVAGIQHNFKMSAFKENCGFFFPEIKRNLGKYVYRCPESVKKWLQQIKDAGKITMLITSSHSDYCKL   233
gi|675654013        NN-GQKTFDFWKDIVAAIQHNYKMSAFKENCGIYFPEIKRDPGRYLHSCPESVKKWLRQLKNAGKILLLITSSHSDYCRL   228
gi|530639256        HD-RQTKYNFWKDVIAAIQHNYKTSAFKENCGTYFPEVKNDPAKYLQSCPESVKKWLRQLKNAGKVLLLITSSHSDYCRL   227
gi|543344792        HN-GQKKYDFWKDMVAAIQHNYKISAFKEDCGMYFPEVKNHPDKYLQRCPESVKKWLKQLKNAGKILLLITSSHSDYCRL   228
gi|542164235        HD-GQKKYDFWKDMVAAIQHNYKISAFKEDCGMYFPEVKNHPDKYLQRCPESVKKWLKQLKNAGKILLLITSSHSDYCRL   228
gi|363732051        HD-GQKKYDFWKDMVAAIQHNYKISAFKEDCGTYFPEVKNHPEKYLQRCPSSVKKWLKQLKSAGKILLLITSSHSDYCRL   228
gi|683891145        HD-GQKKYDFWKDMVAAIQHNYKISAFKEDCGMYFPEVKNHPDKYLQRCPESVKKWLKQLKNAGKILLLITSSHSDYCRL   228
gi|543344789        HN-GQKKYDFWKDMVAAIQHNYKISAFKEDCGMYFPEVKNHPDKYLQRCPESVKKWLKQLKNAGKILLLITSSHSDYCRL   227
gi|449497791        HD-GQKKYDFWKDMVAAIQHNYKISAFKENCGMYFPEVKNHPDKYLQRCPESVKKWLKQLKNAGKILLLITSSHSDYCRL   227
gi|542164233        HD-GQKKYDFWKDMVAAIQHNYKISAFKEDCGMYFPEVKNHPDKYLQRCPESVKKWLKQLKNAGKILLLITSSHSDYCRL   227
gi|50744564         HD-GQKKYDFWKDMVAAIQHNYKISAFKEDCGTYFPEVKNHPEKYLQRCPSSVKKWLKQLKSAGKILLLITSSHSDYCRL   227
gi|729750329        HD-GQKKYDFWKDMVAAIQHNYKISAFKEDCGTYFPEVKNHPDKYLQTCPESVKKWLKQLKSAGKILLLITSSHSDYCRL   227
gi|768365056        HD-GQKKYDFWKDMVAAIQHNYKISAFKEDCGTYFPEVKNHPDRYLQTCPESVKKWLKQLKSAGKILLLITSSHSDYCRL   227
                    .......170.......180.......190.......200.......210.......220.......230.......240
```

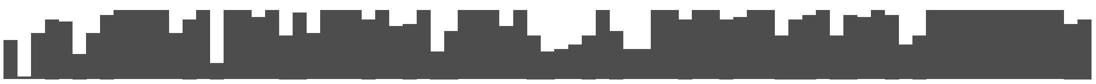

```
                 *  : ** **   **::***********: *.**.*  *:.*:*:: *  ::*********  :* :******.: .*:*
lcl|Query_37229  LCEYILGNDFTDLFDIVITNALKPGFFSHLPSQRPFRTLENDEEQEALPSLDKPGWYSQGNAVHLYELLKKMTGKPEPKV   308
gi|114608985     LCEYILGNDFTDLFDIVITNALKPGFFSHLPSQRPFRTLENDEEQEALPSLDKPGWYSQGNAVHLYELLKKMTGKPEPKV   308
gi|426354317     LCEYILGNDFTDLFDIVITNALKPGFFSHLPSQRPFRTLENDEEQEALPSLDKPGWYSQGNAVHLYELLKKMTGKPEPKV   308
gi|795513538     LCEYILGNDFTDLFDVVITNALKPGFFSHLPSQRPFRTLENDEEQEALPSLDKPGWYSQGNAVHLYELLKKMTGKPEPKV   308
gi|387763464     LCEYILGNDFTDLFDVVITNALKPGFFSHLPSQRPFRTLENDEEQEALPSLDKPGWYSQGNAVHLYELLKKMTGKPEPKV   308
gi|795549508     LCEYILGNDFTDLFDVVITNALKPGFFSHLPSQRPFRTLENDEEQEALPSLDKPGWYSQGNAVHLYELLKKMTGKPEPKV   308
gi|795513542     LCEYILGNDFTDLFDVVITNALKPGFFSHLPSQRPFRTLENDEEQEALPSLDKPGWYSQGNAVHLYELLKKMTGKPEPKV   307
gi|544424790     LCEYILGNDFTDLFDVVITNALKPGFFSHLPSQRPFRTLENDEEQEALPSLDKPGWYSQGNAVHLYELLKKMTGKPEPKV   307
gi|724807254     LCEYILGNDFTDLFDIVITNALKPGFFSHLPSQRPFRTLENDEEQEALPSLDKPGWYSQGNAVHLYELLKKMTGKPEPKV   308
gi|795130302     LCEYILGNDFTDLFDIVITNALKPGFFSHLPSQRPFRTLENDEEQEALPSLDKPGWYSQGNAVHLYELLKKMTGKPEPKV   308
gi|817291021     LCDYILGNDFADLFDIVITNALKPGFFSHLPNQRPFRTLENDEEQEALPSLDKPGWYSQGNAVHLYELLKKMTGKPEPKV   308
gi|296199057     LCEYILGNDFADLFDIVITNALKPGFFSHLPNQRPFRILENDEEQEALPSLDKPGWYSQGNAVHLYELLKKMTGKPEPKV   308
gi|724807257     LCEYILGNDFTDLFDIVITNALKPGFFSHLPSQRPFRTLENDEEQEALPSLDKPGWYSQGNAVHLYELLKKMTGKPEPKV   307
gi|403295527     LCEYILGNDFADLFDIVITNALKPGFFSHLPNQRPFRTLENDEEQEALPSLDKPGWYSQGNAVHLYELLKKMTGKPEPKV   308
gi|817291023     LCDYILGNDFADLFDIVITNALKPGFFSHLPNQRPFRTLENDEEQEALPSLDKPGWYSQGNAVHLYELLKKMTGKPEPKV   307
gi|675654011     LCEYILGNDFADLFDIVITNALKPGFFSHLPNQRPFRILENDEEQEALPSLDKPGWYSQGNAVHLYELLKKMTGKPEPKV   307
gi|725594041     LCEYILGNDFADLFDIVITNALKPGFFSHLPNQRPFRTLENDEEQEALPSLDKPGWYSQGNAVHLYELLKKMTGKPEPKV   307
gi|829988711     LCEYILGNDFADLFDIVITNALKPGFFSHLPSQRPFRTLENDEEQEALPSLEKPGWYSQGNVGHLYELLKKMTGKPEPKV   308
gi|395816322     LCEYILGNDFADLFDVVITNALKPGFFSHLPSQRPFRTLENDEEQEALPSLDKPGWYSQGNAVHLYELLKKMTGKPEPKV   308
gi|826310252     LCEYILGNDFADLFDIVITNALKPGFFSHLPSQRPFRTLENDEEQEALPSLEKPGWYSQGNVGHLYELLKKMTGKPEPKV   308
gi|410959884     LCEYILGNDFTDLFDIVITNALKPGFFSHLPSQRPFWTLENDEEQEALPSLDKPGWYSQGNAVHLYELLKKMTGKSEPKV   308
gi|585164297     LCEYILGNDFTDLFDIVITNALKPGFFSHLPSQRPFWTLENDEEQEALPSLDKPGWYSQGNAVHLYELLKKMTGKNEPKV   308
gi|586987268     LCEYILGNDFTDLFDIVITNALKPGFFSHLPSQRPFWTLENDEEQEALPSLDKPGWYSQGNAVHLYELLKKMTGKSEPKV   307
gi|472356764     LCEYILGNDFTDLFDIVITNALKPGFFSHLPSQRPFWTLENDEEQEALPSLDKPGWYSQGNAVHLYELLKKMTGKNEPKV   308
gi|602685616     LCEYILGNDFEYLFDIVITNALKPGFFSHLPSQRPFRTLENDEEQEALPSLDKPGWYSQGNAVHLYELLKKMTGKPEPKV   308
gi|667279819     LCKYILGNDFADLFDIVITNALKPGFFSHLPSQRPFRTLENDEEQEALPSLDKPGWYSQGNAVHLYELLKKMTGKPEPKV   307
gi|667279822     LCKYILGNDFADLFDIVITNALKPGFFSHLPSQRPFRTLENDEEQEALPSLDKPGWYSQGNAVHLYELLKKMTGKPEPKV   306
gi|465979262     LCEYILGNDFEYLFDIVITNALKPGFFSHLPSQRPFRTLENDQEQEALPSLDKPGWYSQGNAVHLYELLKKMTGKPEPKV   308
gi|194035204     LCRYILGNDFEDLFDIVITNALKPGFFSHLPSQRPFRTLENDEEQEALPSLDKPGWYSQGNAVHLYELLKKMTGQPEPKV   308
gi|759133407     LCKHILGNDFADLFDIVITNALKPGFFSHLPSQRPFRTLENDEEQEPLPSLDKPGWYSQGNAVHLYELLKKMTGKPEPKV   308
gi|641734335     LCNHILGNDFADLFDIVITNALKPGFFSHLPSQRPFRTLENDEEQEALPSLDKPGWYSQGNAVHLYELLKKMTGKPEPKV   308
gi|511930642     LCEYILGNDFTDLFDIVITNALKPGFFSHSPSQRPFWTLENDEEQEALPSLDKPGWYSQGNAVHLYELLKKMTGKNEPKV   308
gi|744561719     LCEYILGNDFEDLFDIVITNALKPGFFSHSPSQRPFRTLENDEEQEALPSLDKPGWYSQGNAVHLYELLKKMTGKPEPKV   307
gi|558121862     LCNHILGNDFADLFDIVITNALKPGFFSHLPSQRPFRTLENDEEQEALPSLEKPGWYSQGNAVHLYDLLKKMTGKPEPQV   308
gi|478495911     LCEHILGNDFADLFDIVITNALKPGFFSQSPSQRPFRTLENDEEQEALPSLDKPGWYSQGNAVHLNELLKKMTGKPEPKV   308
gi|556739024     LCEYILGNDFEDLFDIVITNALKPGFFSHLPSQRPFRTLENDEEREALPSLDKPGWYSQGNAVHLYELLKKMTGKPEPKV   308
gi|655843044     LCEYILGNDFADLFDIVITNALKPGFFSHLPSQRPFWTLKNDEEQEVLPSLDKPGWYSQGNAVQLYELLKKMTGKPEPKV   308
gi|507925770     LCEYILGNDFPDLFDIIITNALKPGFFSHLPSQRPFWTLENDEEQEELPSLDKPGWYSQGNAVHLYELLKKMTGKPEPKV   308
gi|829980860     LCEYILGNDFPDLFDIIITNALKPGFFSHLPSQRPFWTLENDEEQEELPSLDKPGWYSQGNAVHLYELLKKMTGKPEPKV   307
gi|426234499     LCEYILGNDFEDLFDIVITNALKPGFFSHLPSQRPFRTLENDEEREALPSLDKPGWYSQGNAVHLYELLKKMTGKPEPKV   308
gi|157428094     LCQYILGNDFEDLFDIVITNALKPGFFSHLPSQRPFRTLENDEEREALPFLDKPGWYSQGNAVHLYELLKKMTGKPEPKV   308
gi|594063404     LCQYILGNDFEDLFDIVITNALKPGFFSHLPSQRPFRTLENDEEREALPFLDKPGWYSQGNAVHLYELLKKMTGKPEPKV   308
gi|803097535     LCEYILGNDFEDLFDIVITNALKPGFFSHLPSQRPFRTLENDEEREALPSLDKPGWYSQGNAVHLYELLKKMTGKPEPKV   307
gi|670997190     LCEYILGNDFTDLFDIVITNALKPGFFSHLPSQRPFWTLEKDEEQEALPSLDKPGWYSQGNAVHLYELLKKMTGRNEPKV   283
gi|548479506     LCEYILGNDFEDLFDIVITNALKPGFFSHLPSQRPFRTLENDEEREALPSLDKPGWYSQGNAAHLYELLKKMTGKPEPKV   308
gi|555991352     LCQYILGNDFEDLFDIVITNALKPGFFSHLPSQRPFWTLENDEEREALPFLDKPGWYSQGNAVHLYELLKKMTGKPEPKV   308
gi|528960483     LCQYILGNDFEDLFDIVITNALKPGFFSHLPSQRPFRTLENDEEREALPFLDKPGWYSQGNAVHLYELLKKMTGKPEPKV   307
gi|594063406     LCQYILGNDFEDLFDIVITNALKPGFFSHLPSQRPFRTLENDEEREALPFLDKPGWYSQGNAVHLYELLKKMTGKPEPKV   307
gi|548479508     LCEYILGNDFEDLFDIVITNALKPGFFSHLPSQRPFRTLENDEEREALPSLDKPGWYSQGNAAHLYELLKKMTGKPEPKV   307
gi|664701045     LCEHILGNDFADLFDIVITNALKPGFFSHSPSQRPFRTLENDEEQEALPSLDKPGWYSQGNAAHLYELLKKMTGKPEPKV   278
gi|820999445     LCEYTLGDDFADLFDIVITNALKPGFFSHSPSQRPFHTLKNDEEQEALPSLDKPGWYSQGNAVHLYELLKKMTGKPQPKV   308
gi|554586740     LCNHILGNDFADLFDIVITNALKPGFFSHLPSQRPFRTLENDEEQEALPSLEKPGWYSQGNAVHLYDLLKKMTGKPEPQV   293
gi|584073388     LCNHILGNDFADLFDIVITNALKPGFFSHLPSQRPFRTLENDEEQEALPSLEKPGWYSQGNAAHLYDLLKKMTGKPEPQV   293
gi|558127235     LCNHILGNDFADLFDIVITNALKPGFFSHLPSQRPFRTLENDEEQEALPCLEKPGWYSQGNAVHLYDLLKKMTGKPEPQV   293
gi|512920735     LCEYTLGDDFADLFDIVITNALKPGFFSHSPSQRPFYTLKDDEEQESLPSLDKPGWYSQGNSAHLYELLKKMTSKPKPKV   308
gi|617593341     LCGYILGNDFPDIFDIVITNALKPGFFSHLPNQRPFRTLENDEEQEALPSLDKPGWYSQGNAVHLYELLKKMTGKPEPKV   285
gi|674085287     LCNYILGHDFADFFDIVITNALKPGFFSHFPSQRPFHTLENDEEQEELTSLDKPGWYSQGNAAHLYELLKKMTGKPEPKV   308
gi|820979643     LCEYILGNDFTDLFDIVITNALKPGFFSHLPSQRPFRTLENDEEQEVLPSLDKPGWYSQGNAVHLYELLKKMTGKPEPKV   244
gi|625280199     LGTYILGKDFADLFDIVITNALKPGFFSHFPSQRPFHTLENDEEQEELPSLDKPGWYSQGNAAHLYELLKKMTNKPEPKV   314
gi|532041477     LGTYILGKDFADLFDIVITNALKPGFFSHFPSQRPFHTLENDEEQEELPSLDKPGWYSQGNAAHLYELLKKMTGKPEPKV   314
gi|625280201     LGTYILGKDFADLFDIVITNALKPGFFSHFPSQRPFHTLENDEEQEELPSLDKPGWYSQGNAAHLYELLKKMTNKPEPKV   313
gi|672086605     LGSYILGKDFADLFDIVITNALKPGFFSHFPSQRSFYTLENDEEKDELPSLDKPGWYSQGNAVHLYELLKKMTSKLEPKV   314
gi|110626010     LGSYILGEDFADLFDIVITNALKPGFFSHFPSQRPFYTLENDEEKDELPSLDKPGWYSQGNAAHLYELLKKMTSKPEPKV   318
gi|524945211     LGTYILGKDFADLFDIVITNALKPGFFSHFPSQRPFHTLENDEEQEELPSLDKPGWYSQGNAAHLYELLKKMTSKPEPKV   314
gi|568965457     LGSYILGEDFADLFDIVITNALKPGFFSHFPSQRPFYTLENDEEKDELPSLDKPGWYSQGNAAHLYELLKKMTSKPEPKV   317
gi|672086607     LGSYILGKDFADLFDIVITNALKPGFFSHFPSQRSFYTLENDEEKDELPSLDKPGWYSQGNAVHLYELLKKMTSKLEPKV   313
gi|675654013     LCEYILGNDFADLFDIVITNALKPGFFSHLPNQRPFRILENDEEQEALPSLDKPGWYSQGNAVHLYELLKKMTGKPEPKV   308
gi|530639256     LCEHILGNDFAGLFDIVITNALKPGFFSQMPNQRPFRTLENDEEQEALPSLDKPGWYSQGNATHLYELLKKMTGKPEPKV   307
gi|543344792     LCEHVLGSDFEEYFDILITNALKPGFFSHTPNQRPFRTLENDEEQEALLSVDKPGWYSQGNAIQLYDLLKKMTGKLDPKV   308
gi|542164235     LCEHVLGNDFEEYFDILITNALKPGFFSHTPNQRPFRTLENDEEQEALLSLDKPGWYSQGNAIQLYDLLKKMTGKLDPKV   308
gi|363732051     LCEYILGNDFEEYFDVVITNALKPGFFSHTPNQRPFRTLENDEEQEALLSLDKPGWYSQGNAIHLYELLKKMTGKLDPKV   308
gi|683891145     LCEHVLGNDFREYFDILITNALKPGFFSHTPNQRPFRTLENDEEQEALLSLDKPGWYSQGNALQLYDLLKKMTGKLDPKV   308
gi|543344789     LCEHVLGSDFEEYFDILITNALKPGFFSHTPNQRPFRTLENDEEQEALLSVDKPGWYSQGNAIQLYDLLKKMTGKLDPKV   307
gi|449497791     LCEHVLGNDFEEYFDILITNALKPGFFSHTPNQRPFRTLENDEEQEALLSLDKPGWYSQGNAIQLYDLLKKMTGKLDPKV   307
gi|542164233     LCEHVLGNDFEEYFDILITNALKPGFFSHTPNQRPFRTLENDEEQEALLSLDKPGWYSQGNAIQLYDLLKKMTGKLDPKV   307
gi|50744564      LCEYILGNDFEEYFDVVITNALKPGFFSHTPNQRPFRTLENDEEQEALLSLDKPGWYSQGNAIHLYELLKKMTGKLDPKV   307
gi|729750329     LCEHILGNDFEEYFDIVITNALKPGFFSHTPNQRPFRTLENDEEQEALLSLDKPGWYSQGNAIQLYELLKKMTGKLDPKV   307
gi|768365056     LCEHILGNDFEEYFDIVITNALKPGFFSHTPNQRPFRTLENDEEQEALLSLDKPGWYSQGNAIQLYELLKKMTGKLDPKV   307
                 .......250.......260.......270.......280.......290.......300.......310.......320
```

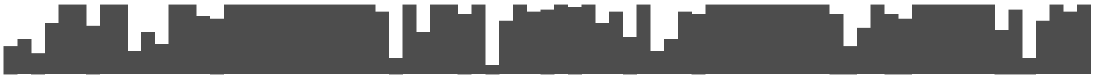

```
                              **********::** ** :****:*****              :** **:**:*          .  * : : *
lcl|Query_37229  VYFGDSMHSDIFPARHYSNWETVLILEELRGDEGTRSQRP-EESEPLEKKGKYEGPKAKPLNTSSKKWGSFFIDSVLGLE  387
gi|114608985     VYFGDSMHSDIFPARHYSNWETVLILEELRGDEGTRSQRP-EESEPLEKKGKYEGPKAKPLNTSSKKWGSFFIDSVLGLE  387
gi|426354317     VYFGDSMHSDIFPARHYSNWETVLILEELRGDEGARSQRP-EESEPLEKKGKYEGPKAKPLNTSSKKWGSFFIDSVLGLE  387
gi|795513538     VYFGDSMHSDIFPARHYSNWETVLILEELRGDEGARSQRP-EESEPLEKKGKYEGPKAKPLNTSSKKWGSFFIDSVWGLE  387
gi|387763464     VYFGDSMHSDIFPARHYSNWETVLILEELRGDEGARSQRP-EESEPLEKKGKYEGPKAKPLNTSSKKWGSFFIDSVWGLE  387
gi|795549508     VYFGDSMHSDIFPARHYSNWETVLILEELRGDEGARSQRP-EESEPLEKKGKYEGPKAKPLNTSSKKWGSFFIDSVWGLE  387
gi|795513542     VYFGDSMHSDIFPARHYSNWETVLILEELRGDEGARSQRP-EESEPLEKKGKYEGPKAKPLNTSSKKWGSFFIDSVWGLE  386
gi|544424790     VYFGDSMHSDIFPARHYSNWETVLILEELRGDEGARSQRP-EESEPLEKKGKYEGPKAKPLNTSSKKWGSFFIDSVWGLE  386
gi|724807254     VYFGDSMHSDIFPARHYSNWETVLILEELRGDEGARSQRP-EESEPLEKKGKYEGPKAKPLNTSSKKWGSFFIDSVWGLE  387
gi|795130302     VYFGDSMHSDIFPARHYSNWETVLILEELRGEEGARSQRP-EESEPLEKKGKYEGPKAKPLNTSSKKWGSFFIDSVWGLE  387
gi|817291021     VYFGDSMHSDIFPARHYSNWETVLILEELRGDEGTRRQRS-EESEPLEKKGKYEGPKAKPLNTPSKKWGSFFIDSVLGLE  387
gi|296199057     VYFGDSMHSDIFPARHYSNWETVLILEELRGDEGTRRQRP-EESEPLEKKGKYEGPKAKPLNTSSKKWGSFFIDSILGLE  387
gi|724807257     VYFGDSMHSDIFPARHYSNWETVLILEELRGDEGARSQRP-EESEPLEKKGKYEGPKAKPLNTSSKKWGSFFIDSVWGLE  386
gi|403295527     VYFGDSMHSDIFPARHYSNWETVLILEELGGDEGTRRQRP-EESEPLEKKGKYEGPKAKLLNTSSKKWGSFFIDSILGLE  387
gi|817291023     VYFGDSMHSDIFPARHYSNWETVLILEELRGDEGTRRQRS-EESEPLEKKGKYEGPKAKPLNTPSKKWGSFFIDSVLGLE  386
gi|675654011     VYFGDSMHSDIFPARHYSNWETVLILEELRGDEGTRRQRP-EESEPLEKKGKYEGPKAKPLNTSSKKWGSFFIDSILGLE  386
gi|725594041     VYFGDSMHSDIFPARHYSNWETVLILEELGGDEGTRRQRP-EESEPLEKKGKYEGPKAKLLNTSSKKWGSFFIDSILGLE  386
gi|829988711     VYFGDSMHSDIFPAHHYSNWETVLILEELKGDEVVRSQRP-EESEPLEKKGKYEGPKVKPLSTSSKKWGSFFIDSVSGLE  387
gi|395816322     VYFGDSMHSDIFPARHYSHWETVLILEELRGDEVVRSQRP-EESEPLEKKGKYEGPKVKPLNISSKKWGSYFTDSVSGLE  387
gi|826310252     VYFGDSMHSDIFPAHHYSNWETVLILEELRGDKVVRSHRP-EESEPLEKKGKYEGPKVKPLSTSSKKWGSFFMDSVSGLE  387
gi|410959884     VYFGDSMHSDIFPARHYSNWETVLILEELRGD---RDGKP-EESEPLEKKGKYEGPKAKPLNTLSKKWGSFFIDSVSGLE  384
gi|585164297     VYFGDSMHSDIFPARHYSNWETVLILEELRGD---RDRKP-EESEPLEKKGKYEGSKAKPLNTLSKKWGSFFIDSVSGLE  384
gi|586987268     VYFGDSMHSDIFPARHYSNWETVLILEELRGD---RDGKP-EESEPLEKKGKYEGPKAKPLNTLSKKWGSFFIDSVSGLE  383
gi|472356764     VYFGDSMHSDIFPARHYSNWETVLILEELRGD---RDWKP-EESEPLEKKGKYEGSKAKPLNTLSKKWGSFFIDSVSGLE  384
gi|602685616     VYFGDSMHSDIFPACHYSNWETVLILEELRGD---RDGKP-EDSEPLEKKGKYEGPKAKPLNRLSKKWGSFFIDSVSGLE  384
gi|667279819     VYFGDSMHSDVFPACHYSNWETVLILEELRGDEG---RKP-EESEPQEKKGKYEGPKAKPLNTLSKKWGSFFIDSVAGLE  383
gi|667279822     VYFGDSMHSDVFPACHYSNWETVLILEELRGDEG---RKP-EESEPQEKKGKYEGPKAKPLNTLSKKWGSFFIDSVAGLE  382
gi|465979262     VYFGDSMHSDIFPACHYSNWETVLILEELRGD---RDGKP-EDSEPLEKKGKYEGLKAKPLNRLSKKWGSFFIDSVSGLE  384
gi|194035204     VYFGDSMHSDIFPASHYSNWETVLILEELRGD---RDGKP-EDAEPLEKKGKYEGPKAKPLNALSKKWGSFFIDSVSGLE  384
gi|759133407     VYFGDSMHSDIYPARHYSNWETVLILEELKGE---RDVKP-EESEPLEKRGKYEGQKTKPLNSSSKKWGSFFIDSVSRLE  384
gi|641734335     VYFGDSMHSDIFPARHYSNWETVLILEELKGD---RDGKP-EESEPREKKGKYEGQKGKPLYSLSKKWGSFFTDSISGLE  384
gi|511930642     VYFGDSMHSDIFPARHYSNWETVLILEELRGD---RDAKP-EESEPLEKKGKYEGQKAKPLNALSKKWGSFFIDSVSGLE  384
gi|744561719     VYFGDSMHSDIFPARHYSNWETVLILEELSGD---REGKP-EESEPLEKKGKYEGPKAKALNTLSKKWGSFFIDSVSGLE  383
gi|558121862     VYFGDSMHSDIFPARHYSNWETVLILEELKGD---RDWKP-EDSEPLEKKGKYEGQKGKPLYSLSKKWGSFFTDSISGLE  384
gi|478495911     VYFGDSMHSDIFPARHYSNWETVLILEELRAD---RDGKP-DESEPLEKKGKYEGPKAKPLNTLSKKWGSFLIDSISGLE  384
gi|556739024     VYFGDSMHSDIFPACHYSNWETVLILEELRGD---KDGKP-EASEPEEKKGKYEGSKAKPLNTSSKKWGSFFIDSVSGLG  384
gi|655843044     VYFGDSMHSDVFPARHYSNWETVLILEELRGQEG---GNP-EESEPLEKKGKFEEPKAKPLNTLSKKWGSFFIDSISGLE  384
gi|507925770     VYFGDSMHSDIFPARHYRNWETVLILEELRED---RDETA-EESEPLEKKGKYEEPKSKPLNTSSKKWGSFFVDSISGLE  384
gi|829980860     VYFGDSMHSDIFPARHYRNWETVLILEELRED---RDETA-EESEPLEKKGKYEEPKSKPLNTSSKKWGSFFVDSISGLE  383
gi|426234499     VYFGDSMHSDIFPACHYSNWETVLILEELRGD---KDGKP-EESEPEEKKGKYEGSKAKPLNTSSKKWGSFFIDSVSGLG  384
gi|157428094     VYFGDSMHSDIFPACHYSNWETVLILEELRGD---KDGKP-EESEPEEKKGKYEGSKAKPLNTSSKKWGSFFIDSVSGLE  384
gi|594063404     VYFGDSMHSDIFPACHYSNWETVLILEELRGD---KDGKP-EESEPEEKKGKYEGSKAKPLNTSSKKWGSFFIDSVSGLE  384
gi|803097535     VYFGDSMHSDIFPACHYSNWETVLILEELRGD---KDGKP-EESEPEEKKGKYEGSKAKPLNTSSKKWGSFFIDSVSGLG  383
gi|670997190     VYFGDSMHSDIFPARHYSNWETVLILEELRGD---RDGKP-EESEPLEKKGKYEGPKAKPLNTLSKKWGSFFIDSVSGLE  359
gi|548479506     VYFGDSMHSDIFPACHYSNWETVLILEELRGD---KDGKP-EESEPEEKKGKYEGSKAKPLNTSSKKWGSFFIDSASGLG  384
gi|555991352     VYFGDSMHSDIFPACHYSNWETVLILEELRGD---KDGKP-EESEPEEKKGKYEGSKAKPLNTSSKKWGSFFIDSVSGLE  384
gi|528960483     VYFGDSMHSDIFPACHYSNWETVLILEELRGD---KDGKP-EESEPEEKKGKYEGSKAKPLNTSSKKWGSFFIDSVSGLE  383
gi|594063406     VYFGDSMHSDIFPACHYSNWETVLILEELRGD---KDGKP-EESEPEEKKGKYEGSKAKPLNTSSKKWGSFFIDSVSGLE  383
gi|548479508     VYFGDSMHSDIFPACHYSNWETVLILEELRGD---KDGKP-EESEPEEKKGKYEGSKAKPLNTSSKKWGSFFIDSASGLG  383
gi|664701045     VYFGDSMHSDIFPARHYSNWETVLILEELRGD---RDGKP-EESEPLEKKGKYEGPKAKPLYTLSKTWGSFFIDSVSGLE  354
gi|820999445     VYFGDSMHSDIFPASHYSKWETVLILEELRGEEG----EK-HEAEPLEKRGKYEGPKAKPLNTLSKKWGSFFIDSVSGQE  383
gi|554586740     VYFGDSMHSDIFPARHYSNWETVLILEELKGD---RDWKP-EDSEPLEKKGKYEGQKGKPLYSLSKKWGSFFTDSISGLE  369
gi|584073388     VYFGDSMHSDIFPARHYSNWETVLILEELKGD---RDWKP-EESEPLEKKGKYEGQKGKPLYSLSKKWGSFFTDSISGLE  369
gi|558127235     VYFGDSMHSDIFPARHYSNWETVLILEELKGD---RDWKP-EDSEPLEKKGKYEGQKGKPLYSLSKKWGSFFTDSISGLE  369
gi|512920735     VYFGDSMHSDIFPASHYSHWETVLILEELRGEEG----EKPEEAEPLEKKGKYEEPKAKPLNTLSKKWGSFFIDSVSERE  384
gi|617593341     VYFGDSMHSDIFPARHYRNWETVLILEELRGD---RDWKP-EESEPVEKRGKYEDPKAKPLNTLSKKWGSFFIDSVSGLE  361
gi|674085287     VYFGDSMHSDVFPARHYSNWETVLILEELRGEED---EKP-EEAEPPEKRGKYEEPKVKPLNTLSNKWGSYFIDSVSRQG  384
gi|820979643     VYFGDSMHSDIFPARHYSNWETVLILEELRGDEGARSQRP-EGSEPLEKKGKYEGPKAKPLNTSSKKWGSFFIDSVLGLE  323
gi|625280199     VYFGDSMHSDIFPAHHYSNWETVLILEELRGQEV----KA-EEAEPLEKRGKYEPPKVKPLNSLSNKWGSYFIDSVSRRG  389
gi|532041477     VYFGDSMHSDIFPAHHYRNWETVLILEELRGQDM---EKA-EEAEPLEKRGKYEAPKVKPLYSLSNKWGSYFIDSVSRRG  390
gi|625280201     VYFGDSMHSDIFPAHHYSNWETVLILEELRGQEV----KA-EEAEPLEKRGKYEPPKVKPLNSLSNKWGSYFIDSVSRRG  388
gi|672086605     VYFGDSMHSDIFPAHHYTNWETVLILEELQGQEM---EKP-EEAEPLEKRGKYEGPKVKPLNCLSNKWGSYFIDSVSRRG  390
gi|110626010     VYFGDSMHSDIFPAHHYTNWETVLILEELQGPEM---EKP-EEAEPLEKRGKYEAPMVKPLNTLSNKWGSYFIDSVSGRG  394
gi|524945211     VYFGDSMHSDIFPAHHYSNWETVLILEELRGQEV---EKA-EKAEPLEKRGKYEEPKVKSLNSLSNKWGSYFIDSVSRRG  390
gi|568965457     VYFGDSMHSDIFPAHHYTNWETVLILEELQGPEM---EKP-EEAEPLEKRGKYEAPMVKPLNTLSNKWGSYFIDSVSGRG  393
gi|672086607     VYFGDSMHSDIFPAHHYTNWETVLILEELQGQEM---EKP-EEAEPLEKRGKYEGPKVKPLNCLSNKWGSYFIDSVSRRG  389
gi|675654013     VYFGDSMHSDIFPARHYSNWETVLILEELRGDEGTRRQRP-EESEPLEKKGKYE----------NYLWTTNLQDSLQAIP  377
gi|530639256     VYFGDSMHSDIFPARHYSNWETVFILEELRGDEV-MIPVE-TESEPLEKKGKYEGHHPKAPYSVSKQWGSFFVDSISGLE  385
gi|543344792     VYFGDSMHSDIFPARHYSNWETVFILEELLGDKG-TVPAK-AESEPLEKKGKYEGDQLKVPYFVSKQWGSFFMDRLPGLE  386
gi|542164235     VYFGDSMHSDIFPARHYSNWETVFILEELLGDKG-IVPAK-AESEPLEKKGKYEGDQLKVPYFVSKQWGSFFMDRLPGLE  386
gi|363732051     VYFGDSMHSDIFPARHYSNWETVFILEELLGDKV-MVPTE-TESEPLEKKGKYEGYQPKIPYSVSEQWGSFFVDRLPGLE  386
gi|683891145     VYFGDSMHSDIFPARHYSNWETVFILEELLGDEG-IVPAK-AESEPLEKKGKYEGDQLKVPYFVSKQWGSFFRDRLPGLE  386
gi|543344789     VYFGDSMHSDIFPARHYSNWETVFILEELLGDKG-TVPAK-AESEPLEKKGKYEGDQLKVPYFVSKQWGSFFMDRLPGLE  385
gi|449497791     VYFGDSMHSDIFPAHHYSNWETVFILEELLGDKG-IEPAK-AESEPLEKKGKYEGDQLKVPYFVSKQWGSFFVDRLPGLE  385
gi|542164233     VYFGDSMHSDIFPARHYSNWETVFILEELLGDKG-IVPAK-AESEPLEKKGKYEGDQLKVPYFVSKQWGSFFMDRLPGLE  385
gi|50744564      VYFGDSMHSDIFPARHYSNWETVFILEELLGDKV-MVPTE-TESEPLEKKGKYEGYQPKIPYSVSEQWGSFFVDRLPGLE  385
gi|729750329     VYFGDSMHSDIFPARHYSNWETVFILEELLGDKV-TLPAE-TESEPLEKKGKYEGDQLKAPYFVSKQWGSFFVDRLPGLE  385
gi|768365056     VYFGDSMHSDIFPARHYSNWETVFILEELLGDKV-TLPAE-TESEPLEKKGKYEGDQLKAPYFVSKQWGSFFMDRLPGLE  385
                 .......330.......340.......350.......360.......370.......380.......390.......400
```

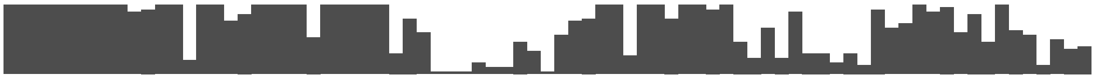

```
                                .  :  :  .*:  *           ::.: :
lcl|Query_37229     NTEDSLVYTWSCKRISTYSTIAIPSIEAIAELPLDYKFTRFSSSNSKTAGYYPNPPLVLSS---DETLISK--   455
gi|114608985        NTEDSLVYTWSCKRISTYSTIAIPSIEAIAELPLDYKFTRFSSSNSKTAGYYPNPPLVLSS---DETLISK--   455
gi|426354317        NTEDSLVYTWSCKRISTYSTIAIPSIEAIAELPLDYKFTRFSSSNSKTAGYYPNPPLVLSS---DETLISK--   455
gi|795513538        NTEDSLVYTWSCKRISTYSTIAIPSIEAIAELPLDYKFTRFSSSNSKTAGYYPNPPLVLTS---DETLISK--   455
gi|387763464        NTEDSLVYTWSCKRISTYSTIAIPSIEAIAELPLDYKFTRFSSSNSKTAGYYPNPPLVLTS---DETLISK--   455
gi|795549508        NTEDSLVYTWSCKRISTYSTIAIPSIEAIAELPLDYKFTRFSSSNSKTAGYYPNPPLVLTS---DETLISK--   455
gi|795513542        NTEDSLVYTWSCKRISTYSTIAIPSIEAIAELPLDYKFTRFSSSNSKTAGYYPNPPLVLTS---DETLISK--   454
gi|544424790        NTEDSLVYTWSCKRISTYSTIAIPSIEAIAELPLDYKFTRFSSSNSKTAGYYPNPPLVLTS---DETLISK--   454
gi|724807254        NTEDSFVYTWSCKRISTYSTIAIPSIEAIAELPLDYKFTRFSSSNSKTAGYYPNPPLVLTS---DETLISK--   455
gi|795130302        NTEDSLVYTWSCKRISTYSTIAIPSIEAIAELPLDYKFTRFSSSNSKTAGYYPNPPLVLTS---DETLIPK--   455
gi|817291021        NTEDSLVYTWSCKRISTYSTIAIPSIEAIAELPLDYKFTRFSSSNSKTAGYYPNPPLVLSS---DDTLITK--   455
gi|296199057        NTEDSLVYTWCCKRISTYSTIAIPSIEAIAELPLDYKFTRFSSSNSKTAGYYPNPPLALSS---DDILITK--   455
gi|724807257        NTEDSFVYTWSCKRISTYSTIAIPSIEAIAELPLDYKFTRFSSSNSKTAGYYPNPPLVLTS---DETLISK--   454
gi|403295527        NTEDSLVYTWSCKRISTYSTIAIPSIEAIAELPLDYKFTRFSSSNSKTAGYYPNPPLVLSS---DDTLITK--   455
gi|817291023        NTEDSLVYTWSCKRISTYSTIAIPSIEAIAELPLDYKFTRFSSSNSKTAGYYPNPPLVLSS---DDTLITK--   454
gi|675654011        NTEDSLVYTWCCKRISTYSTIAIPSIEAIAELPLDYKFTRFSSSNSKTAGYYPNPPLALSS---DDILITK--   454
gi|725594041        NTEDSLVYTWSCKRISTYSTIAIPSIEAIAELPLDYKFTRFSSSNSKTAGYYPNPPLVLSS---DDTLITK--   454
gi|829988711        NTEDSLVYTWSSKRISTYSTIAIPSIEAIAELPLDYKFTRFSSSNSKTAGYYPNPPLVLSN---DETLTTK--   455
gi|395816322        NTEDSLVFTWSFKRVGTYSTIAIPSIEAIAELPLDYKFTRFSSSNSKTAGYYPKPPLVLSN---DEMLKTK--   455
gi|826310252        NTEDSLVYTWSCKRISTYSTIAIPSIEAIAELPLDYKFTRFSSSNSKTAGYYPNPPLVL----------IK--   448
gi|410959884        NTEDSLFYTWSCKRISTYSTIAIPSIEAIAELPLDYKFSRFSSSNSKTAGYYPNPPSILSN---NETLTAK--   452
gi|585164297        NTEDSLVYTWSCKRISTYSTIAIPSIEAIAELPLDYKFSRFSSSNSKTAGYYPNPPLILSN---NETLTTK--   452
gi|586987268        NTEDSLFYTWSCKRISTYSTIAIPSIEAIAELPLDYKFSRFSSSNSKTAGYYPNPPSILSN---NETLTAK--   451
gi|472356764        NTEDSLVYTWSCKRISTYSTIAIPSIEAIAELPLDYKFSRFSSSNSKTAGYYPNPPLILTN---NETLTSK--   452
gi|602685616        NTEDSLVYTWSCKRISAYSTIAIPSIEAIAELPLDYKFTSFSSNNSKTAGYYPNPPLVLSN---DGKLTTK--   452
gi|667279819        NTEDSLVCTWTCKRISTYSTIAIPSIEAIAELPLDHKFTRFSSNNSKTSGYYPNPPLVLSN---NETLITK--   451
gi|667279822        NTEDSLVCTWTCKRISTYSTIAIPSIEAIAELPLDHKFTRFSSNNSKTSGYYPNPPLVLSN---NETLITK--   450
gi|465979262        NTEDSLIYTWSCKRISTYSTIAIPSIEAIAELPLDYKFTSFSSNNSKTAGYYPNPPLVLSN---DGKLTTK--   452
gi|194035204        NTKDSLVYTWSCKRISAYSTIAIPSIEAIAELPLDYKFTRFSSNNSKTAGYYPNPPLVLLN---DGMLTAK--   452
gi|759133407        NTEDSLVYTWSCKRISTYSTIAIPSIEAIAELPLDYKFTRFSSNNSKMAGYYPNPPLVLSN---VETRTTK--   452
gi|641734335        NTEDSLVSTWSCKRISTYSTIAIPSIEAIAELPLDYKFTRFSSNSSKTAGYYPNPPLALSS---FETLTNK--   452
gi|511930642        NTEDSLFYTWSCKRISTYSTIAIPSIEAIAELPLDYRFSRFSSSSSKTAGYYPNPPLILSN---NETLTTK--   452
gi|744561719        NTEDSLVYTWSCKRISTYSTIVIPSIEAIAELPLDYKFTRFSSNNSKTAGYYPNPPLVLSS---DGMLTTK--   451
gi|558121862        NTEDSLVSTWSCKRISTYSTIAIPSIEAIAELPLDYKFTRFSSNNSKTAGYYPNPPLALSS---FETLTNK--   452
gi|478495911        NTEDSLVYTWTCKRISTYSTIAIPSIETIAELPLDYKFTRFSSNSSKTAGYYPNPPLVLSN---NETRTTK--   452
gi|556739024        NREDSLVYTWSCKRISAYSTIAIPSIEAIAELPLDYKFTRFSSNNSKTAGYYPNPPLVLSN---AGKLTAK--   452
gi|655843044        NTGDSLVYTWSSKRINTYSTIAIPSIEAIAELPLDYKFTRFSSSNSKTAGYYP---LVH-------------   440
gi|507925770        NKEDSLVYTWSCKRISTYSTIAIPSLETIAELSLDYKFTRFSSNNSKMAGYYPNPPLVLSK----ETSKTK--   451
gi|829980860        NKEDSLVYTWSCKRISTYSTIAIPSLETIAELSLDYKFTRFSSNNSKMAGYYPNPPLVLSK----ETSKTK--   450
gi|426234499        NREDSLVYTWSCKRISAYSTIAIPSIEAIAELPLDYKFTRFSSNNSKTAGYYPNPPLVLSN---AGKLTAK--   452
gi|157428094        NREDSLVYTWSCKRISAYSTIAIPSIEAIAELPLDYKFTRFSSNNSKTAGYYPNPPLVLSN---AGKLTAK--   452
gi|594063404        NREDSLVYTWSCKRISAYSTIAIPSIEAIAELPLDYKFTRFSSNNSKTAGYYPNPPLVLSN---AGKLTAK--   452
gi|803097535        NREDSLVYTWSCKRISAYSTIAIPSIEAIAELPLDYKFTRFSSNNSKTAGYYPNPPLVLSN---AGKLTAK--   451
gi|670997190        NTEDSLVYTWSCKRISTYSTIAIPSLEAIAELPLDYKFSRFCSSNSKTAGYYPNPPLILSN---NETLTTK--   427
gi|548479506        NREDSLVYTWSCKRISAYSTIAIPSIEAIAELPLDYKFTRFSSNNSKTAGYYPNPPLVLSN---AGKLTAK--   452
gi|555991352        NREDSLVYTWSCKRISAYSTIAIPSIEAIAELPLDYKFTRFSSNNSKTAGYYPNPPLVLSN---AGKLTAK--   452
gi|528960483        NREDSLVYTWSCKRISAYSTIAIPSIEAIAELPLDYKFTRFSSNNSKTAGYYPNPPLVLSN---AGKLTAK--   451
gi|594063406        NREDSLVYTWSCKRISAYSTIAIPSIEAIAELPLDYKFTRFSSNNSKTAGYYPNPPLVLSN---AGKLTAK--   451
gi|548479508        NREDSLVYTWSCKRISAYSTIAIPSIEAIAELPLDYKFTRFSSNNSKTAGYYPNPPLVLSN---AGKLTAK--   451
gi|664701045        NTEDSLVYTWSCKRISTYSTIAIPSIEAIAELPLDYKFTRFSSNSSKTAGYYPYPPLVLSN---NEKLTTK--   422
gi|820999445        NTEDSSVYTLSSNKISTYSTIAIPSIEAISELPLDYKFTRFSSSSSKTAGYYP---FVHYT---NGLI--K--   446
gi|554586740        NTEDSLVSTWSCKRISTYSTIAIPSIEAIAELPLDYKFTRFSSNNSKTAGYYPNPPVALSS---FETLTNK--   437
gi|584073388        NTEDSLVSTWSCKRISTYSTIAIPSIEAIAELPLDYKFTRFSSNNSKTAGYYPNPPLALSS---FETLANK--   437
gi|558127235        NTEDSLVSTWSCKRISTYSTIAIPSIEAIAELPLDYKFTRFSSNNSKTAGYYPNPPLALSS---FETLTNK--   437
gi|512920735        NTEDSLVYTWSSNKISTYSTIAIPSLEAIAELPLDYKFTRFSSNSSKTAGYYP---LVHYT---NGLI--K--   447
gi|617593341        NTEDSLIHTWSCKRVSTYSTIAIPSIEAIAELPLDHKFTRFSANNSKTAGYYPHPPLVLLN---DAKLKTK--   429
gi|674085287        NAEDSLVYTWSSKRISTYSTIAIPSLEAIAELPLDYKFTRFSSNNSKTAGYYPLVHYGLGS-----------   445
gi|820979643        NTEDSLVYTWSCKRISTYSTIAIPSIEAIAELPLDYKFTRFSSSNSKTAGYYPNPPLVLSS---DETLISK--   391
gi|625280199        NAEDSLVYTWSSRRISTYSTIAIPSLEAIAELPLDYKFTRFSTNSSKTAGYYPLVHYTLYSQDTDSKIVFTEK   462
gi|532041477        NAEDSLVYTWSSRRISTYSTVAIPSIETIAELPLDYKFTRFSTNNSKTAGYYPPVHYTLGSQDTDSKIILTEK   463
gi|625280201        NAEDSLVYTWSSRRISTYSTIAIPSLEAIAELPLDYKFTRFSTNSSKTAGYYPLVHYTLYSQDTDSKIVFTEK   461
gi|672086605        NAEDSLVYTWSSKRISTYSTIAIPNIESIAELPLDYKFTRFSANNSKTAGYYPLVHYTLASQNTDSKIIST--   461
gi|110626010        RAEDSVVYTWSSKRISTYSTIAIPNIESIAELPLDYKFTRFSTNNSKMAGYYPLVHHTLGSQDTDSKIISTEK   467
gi|524945211        NAEDSLVYTWSSRRISTYSTIAIPSLEVIAELPLDYKFTRFSTNSSKTAGYYPLVHYSLYSQDTDSKIIFTEK   463
gi|568965457        RAEDSVVYTWSSKRISTYSTIAIPNIESIAELPLDYKFTRFSTNNSKMAGYYPLVHHTLGSQDTDSKIISTEK   466
gi|672086607        NAEDSLVYTWSSKRISTYSTIAIPNIESIAELPLDYKFTRFSANNSKTAGYYPLVHYTLASQNTDSKIIST--   460
gi|675654013        ------------KQLATIQTLHWP-----------YQVTTY--------------------------------   395
gi|530639256        SAEETLTNTWSCKCISAYSTIAIPRLEAIADLPLDYRFTRFSSNSLETAGYYPKPPRALLS---NGEPVTTK-   454
gi|543344792        NAEETLVHTWSCKCISTYSTIAIPSLEAIADLPLDYRFTRFSSNSSATAGYYPSPPRALLP---NEDSVTMK-   455
gi|542164235        NAEETLVHTWSCKCISTYSTIAIPSLEAIADLPLDYRFTRFSSNSSATAGYYPSPPRALLP---NEDSVMTK-   455
gi|363732051        NAEETLVRTWSCKCISTYSTIAIPSLEAIADLPLDYRFTRFSSNSSATAGYYPSPPRALLP---SEESVTMK-   455
gi|683891145        NAEETLVHTWSCKCISTYSTIAIPSLEAIADLPLDYRFTRFSSNSSATAGYYPSPPRALLP---NEDSVMMK-   455
gi|543344789        NAEETLVHTWSCKCISTYSTIAIPSLEAIADLPLDYRFTRFSSNSSATAGYYPSPPRALLP---NEDSVTMK-   454
gi|449497791        NAEETLVHTWSCKCISTYSTIAIPSLEAIADLPLDYRFTRFSSNSSATAGYYPSPPRALMP---NEDSVMMK-   454
gi|542164233        NAEETLVHTWSCKCISTYSTIAIPSLEAIADLPLDYRFTRFSSNSSATAGYYPSPPRALLP---NEDSVMTK-   454
gi|50744564         NAEETLVRTWSCKCISTYSTIAIPSLEAIADLPLDYRFTRFSSNSSATAGYYPSPPRALLP---SEESVTMK-   454
gi|729750329        NAEETLVRTWSCKCISTYSTIAIPSLEAIADLPLDYRFTRFSSNSSATAGYYPSPPRALLP---NEESVTMK-   454
gi|768365056        NAEETLVRTWSCKCISTYSTIAIPSLEAIADLPLDYRFTRFSSNSSATAGYYPSPPRALLP---NEESVTMK-   454
                    .......410.......420.......430.......440.......450.......460.......470...
```

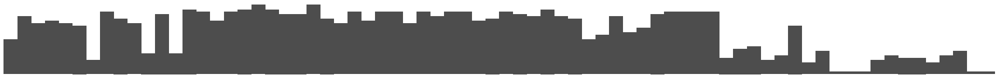

Supplement: Supplementary Figure 4 [file tp2016196x11.pdf]
