# Supplementary material for: Exome chip analyses in adult attention deficit hyperactivity disorder
Source: Transl Psychiatry. 2016 Oct 18;6(10):e923–. doi: 10.1038/tp.2016.196 (PMC5315553; doi:10.1038/tp.2016.196)

Supplementary Figure 5. Plots of the four loci reaching study-wide significance in gene-based analyses of rare SNVs (MAF<1%).

Chromosomal details of four study-wide significant loci.

A) 6q22.1 locus

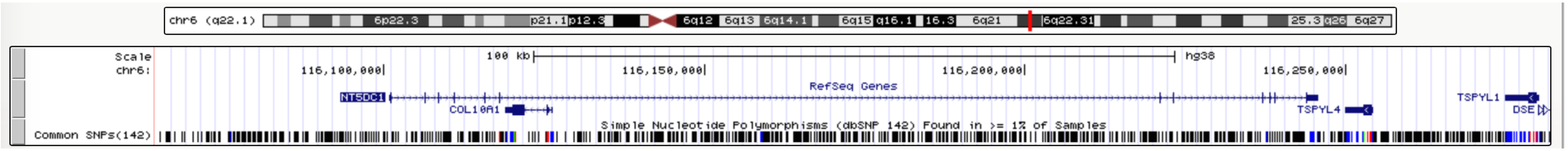

B) PSD locus

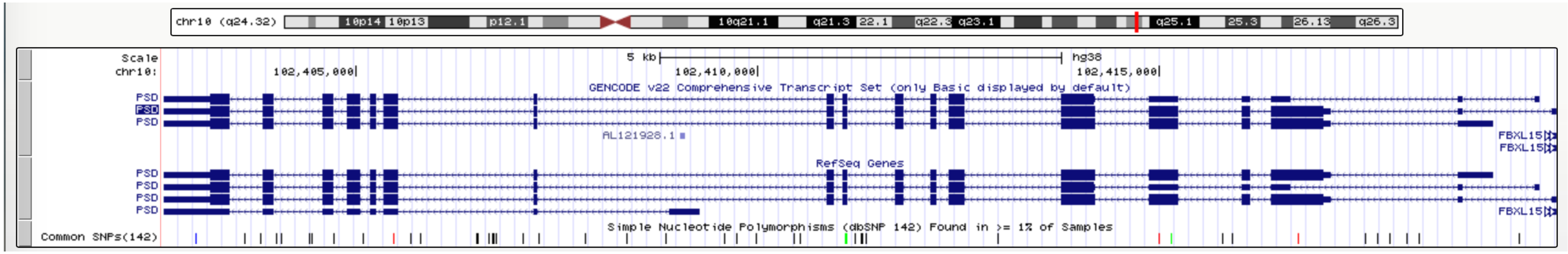

C) SEC23IP locus

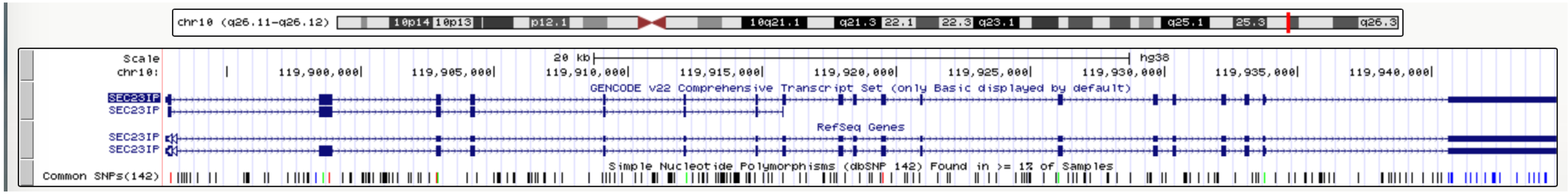

D) ZCCHC4

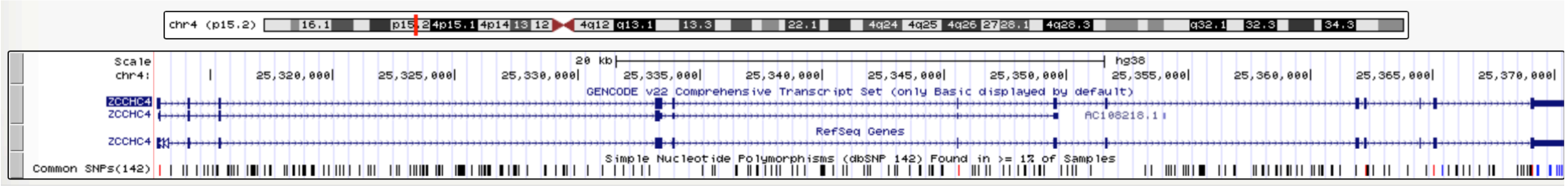

Supplement: Supplementary Figure 5 [file tp2016196x12.pdf]
