# Supplementary material for: Exome chip analyses in adult attention deficit hyperactivity disorder
Source: Transl Psychiatry. 2016 Oct 18;6(10):e923–. doi: 10.1038/tp.2016.196 (PMC5315553; doi:10.1038/tp.2016.196)
Supplement: Supplementary Figure 6 [file tp2016196x13.pdf]

Supplementary Figure 6. Differential expression of the study-wide significant loci in the human brain across the lifetime.

Developmental age is shown on the x axes, in days. Brain regions are abbreviated as follows: NCX, neocortex; STR, striatum; HIP, hippocampus; MD, mediodorsal nucleus of the thalamus; AMY, amygdala; CBC, cerebellar cortex.

A) NT5DC1

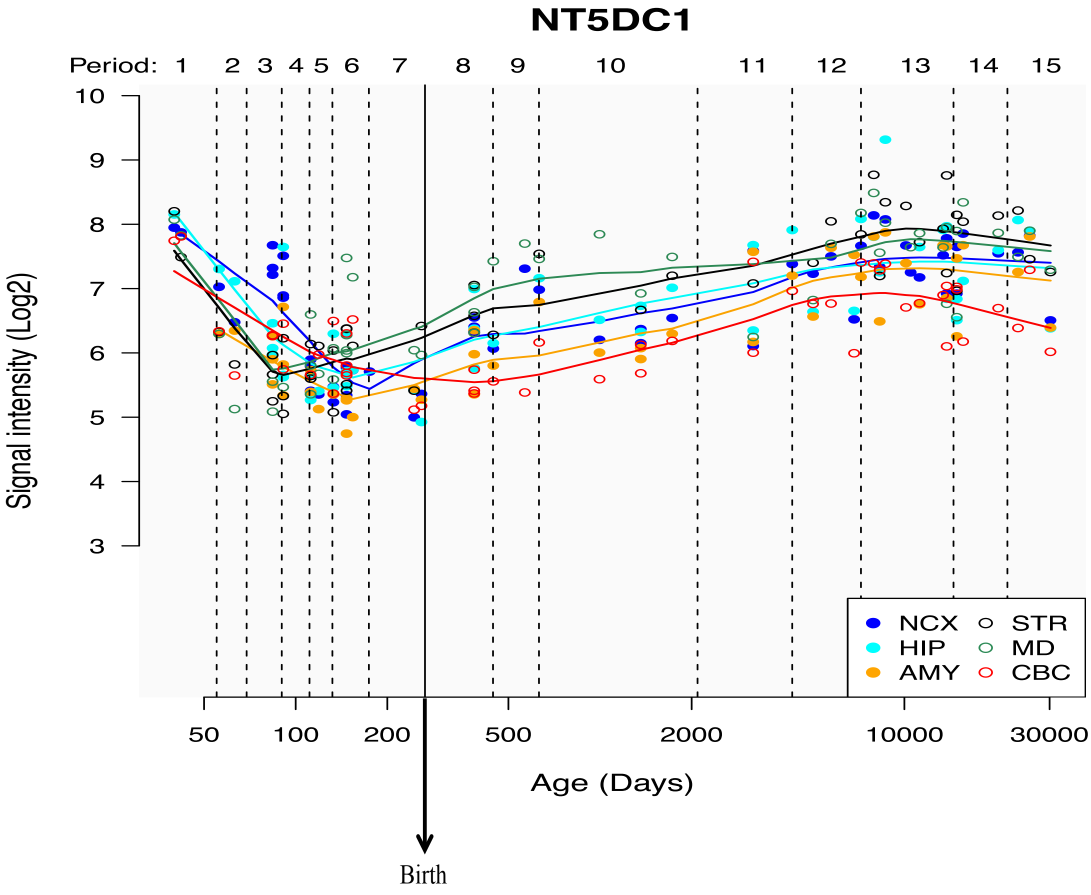

B) SEC23IP

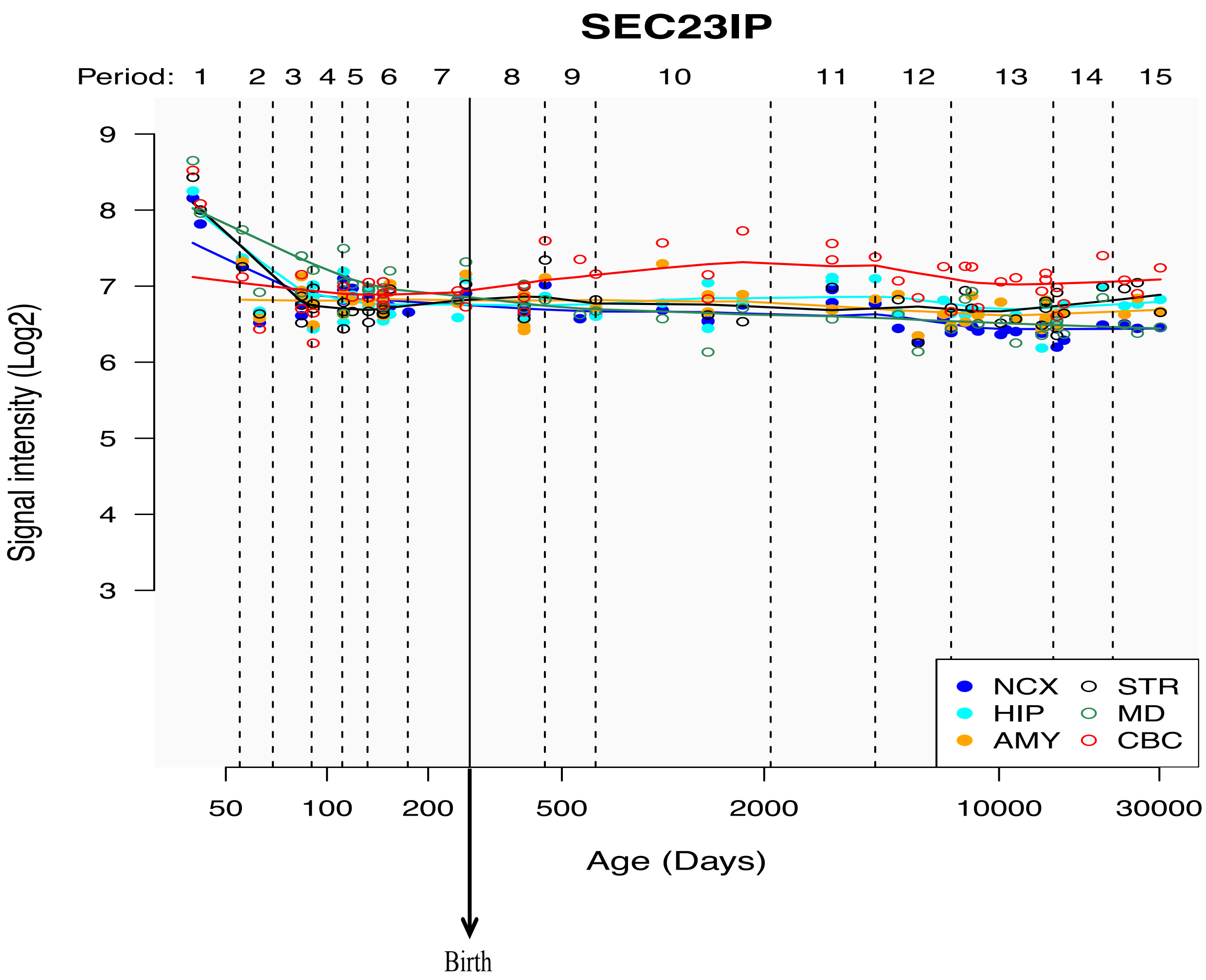
SEC23IP
Period: 1 2 3 4 5 6 7 8 9 10 11 12 13 14 15
Signal intensity (Log2)
9
8
7
6
5
4
3
50 100 200 500 2000 10000 30000
Age (Days)
NCX
HIP
AMY
STR
MD
CBC
Birth

C) PSD

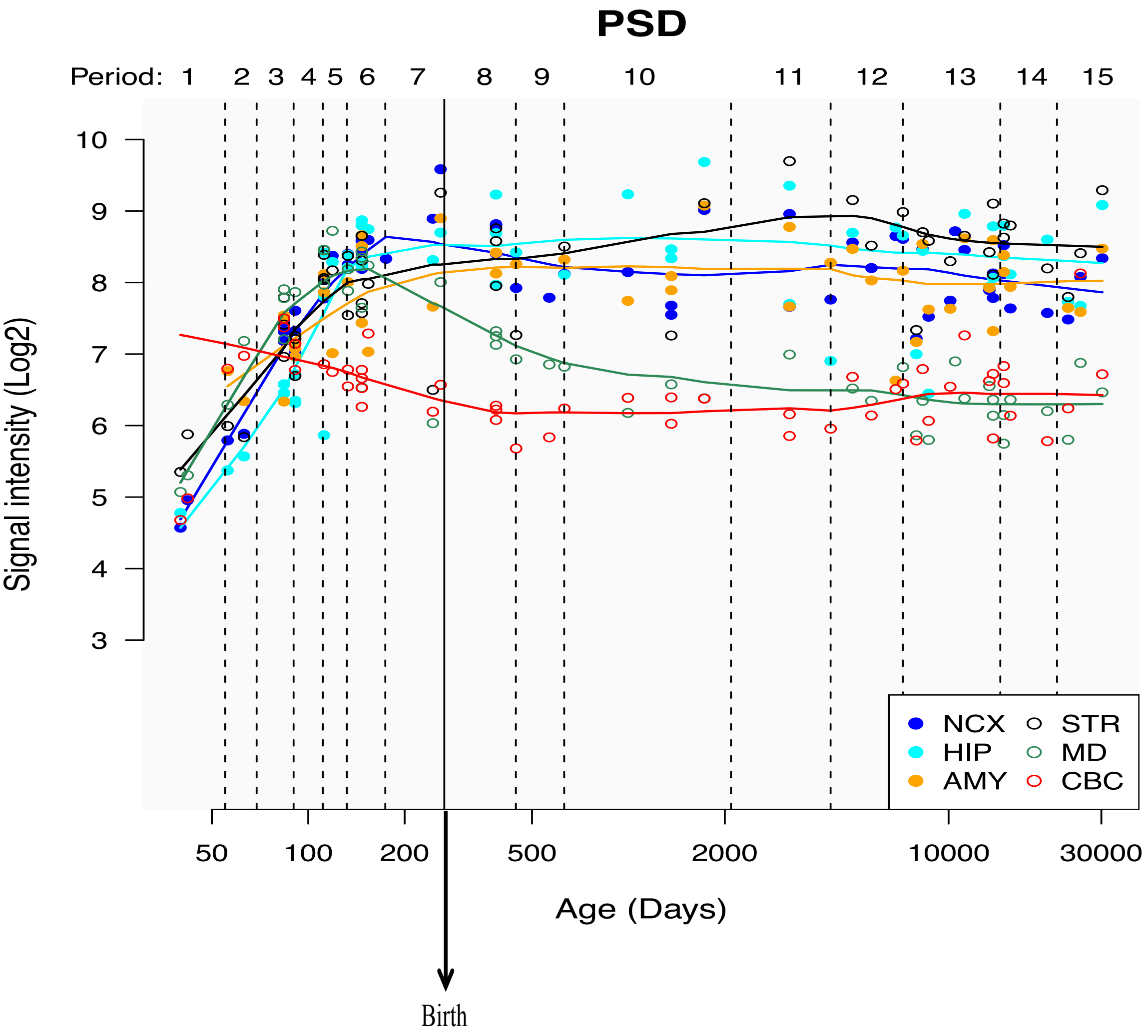
PSD
Period: 1 2 3 4 5 6 7 8 9 10 11 12 13 14 15
10
9
8
7
6
5
4
3
Signal intensity (Log2)
50 100 200 500 2000 10000 30000
Age (Days)
NCX
HIP
AMY
STR
MD
CBC
Birth

D) ZCCHC4

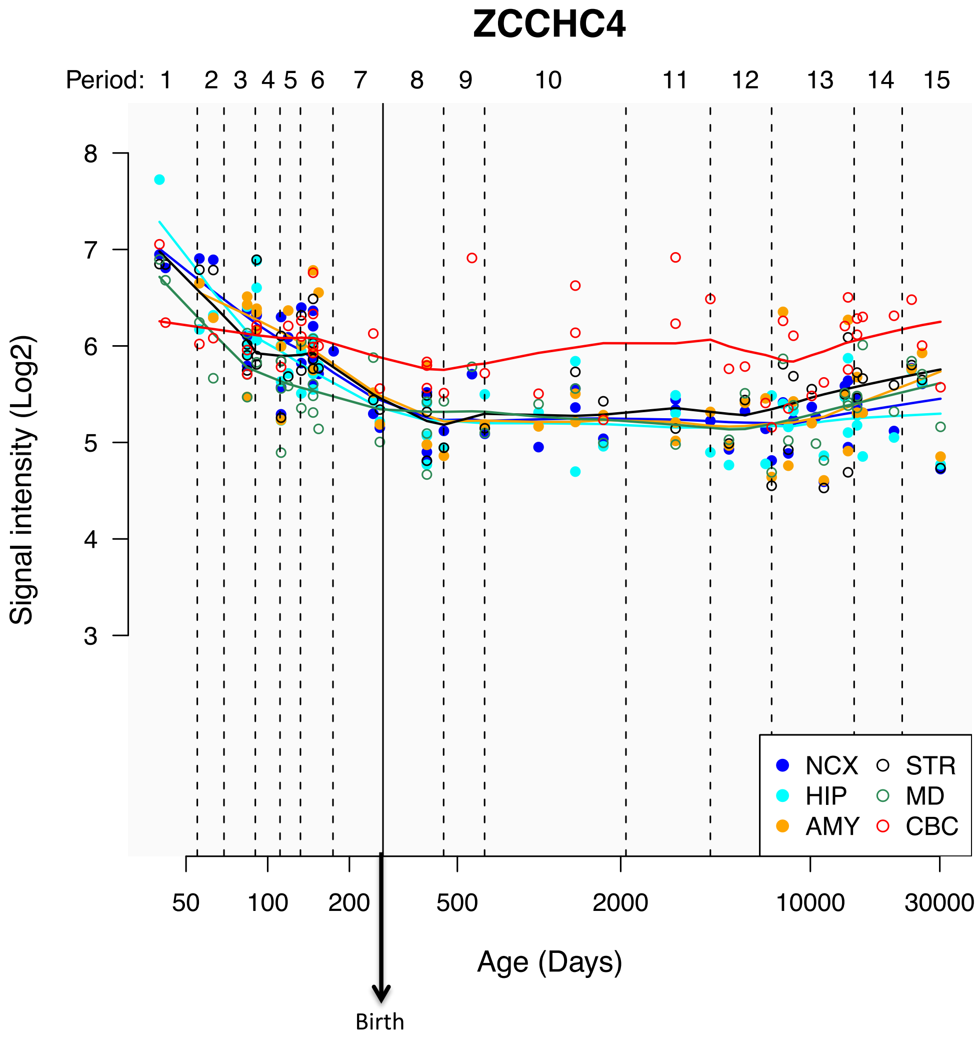

ZCCHC4
Period: 1 2 3 4 5 6 7 8 9 10 11 12 13 14 15
Signal intensity (Log2)
8
7
6
5
4
3
NCX
HIP
AMY
STR
MD
CBC
50
100
200
500
2000
10000
30000
Age (Days)
Birth
