# Supplementary material for: Exome chip analyses in adult attention deficit hyperactivity disorder
Source: Transl Psychiatry. 2016 Oct 18;6(10):e923–. doi: 10.1038/tp.2016.196 (PMC5315553; doi:10.1038/tp.2016.196)

**Supplementary Appendix 1: information on the examined samples**

IMpACT Germany

For discovery and replication stage, patients with a diagnosis of adult ADHD were recruited by experienced psychiatrists at the University of Würzburg (Würzburg, Germany). Unrelated in– and outpatients of self-reported central-European descent completed a semi-structured clinical interview according to DSM-IV. Inclusion criteria were onset before the age of 7 years, life-long persistence, current diagnosis and age of recruitment between 18 and 65 years. The study was approved by the Ethic Committee of the University of Würzburg (Würzburg, Germany). For a more detailed sample description, please confer previous publications (1, 2).

The Heinz Nixdorf Recall (Risk Factors, Evaluation of Coronary Calcium and Lifestyle, HNR) Study is an ongoing population-based, prospective cohort study of the comparative value of modern risk stratification methods with myocardial infarction and cardiac death being primary endpoints (3). The study base is the general German population, randomly selected from statutory lists of residence, living in the three adjacent cities of Bochum, Essen and Mülheim in Western Rhine area of Germany.

IMpACT Norway

ADHD patients were recruited through a Norwegian national medical registry as well as by psychologists and psychiatrists working at out-patient clinics. ADHD diagnosis was defined according to DSM-IV criteria. Controls were randomly recruited through the Norwegian Medical Birth registry. All patients were formally diagnosed with ADHD before inclusion into the project. Recruitment of participants at UiB is described in details elsewhere (4). The study was approved by the regional committee for medical and health research ethics, western Norway.

Controls were recruited through the population based Nord-Trøndelag Health Study (HUNT) in Norway ([www.ntnu.edu/hunt](http://www.ntnu.edu/hunt)) (5). Young-HUNT was approved by the Norwegian Data Inspectorate, the Regional and National Committees for Medical and Health Research Ethics and the Norwegian Directorate of Health. Recruited healthy individuals without cardiovascular disease where genotyped at the Genomic Core Facility, Norwegian University of Science and Technology, Norway, using the HumanExome-12v1_A BeadChip (Illumina) and the Infinium HD ultra protocol as previously described (6).

IMpACT Spain

Participants were recruited at the Department of Psychiatry from the Hospital Universitari Vall d'Hebron (HUVH, Barcelona, Spain) as described elsewhere (7). Patients were adults of Caucasian origin and met Diagnostic and Statistical Manual for Mental Disorders-IV (DSM-IV) criteria for ADHD. The diagnosis of ADHD was evaluated with the Structured Clinical Interview for DSM-IV Axis I and II Disorders (SCID-I and SCID-II) and the Conner’s Adult ADHD Diagnostic Interview for DSM-IV (CAADID Parts I and II). The study was approved by the ethics committee of the institution. DNA was extracted from either peripheral blood or saliva samples.

IMpACT The Netherlands

Patients had been referred for assessment of ADHD to the outpatient clinic of GGZ Delfland in Delft, to Parnassia, psycho-medical centre in The Hague, or to the department of Psychiatry at the Radboud University Nijmegen Medical Centre in Nijmegen, the Netherlands. Patients have been described before (8). This study was approved by the regional ethics committee (Centrale Commissie Mensgebonden Onderzoek: CMO Regio Arnhem—Nijmegen; Protocol number III.04.0403).

Subjects were included if a clinical diagnosis of adult ADHD with childhood onset was established. Prior to inclusion, all patients had undergone a standard clinical assessment consisting of a psychiatric evaluation by experienced psychiatrists. For all included patients a semi-structured diagnostic interview for ADHD and comorbid disorders was also available. For current ADHD symptoms during the last 6 months, a Dutch version of the DSM-IV ADHD Rating Scale, (ADHD-RS) based on the 18 DSM-IV items for ADHD, was used (9). Full diagnostic criteria have been described in more detail elsewhere (10).

Controls for the discovery analyses were derived from the Nijmegen Biomedical Study (NBS, www.nijmegenbiomedischestudie.nl), a population-based survey conducted by the Departments of Epidemiology & Biostatistics and of Clinical Chemistry of the Radboud University Medical Center (11). Approval to conduct the study was obtained from the Institutional Review Board. Controls were unselected for ADHD symptoms.

For replication of the top-findings for common variants, we used samples from the longitudinal NeuroIMAGE sample (12). The NeuroIMAGE study was approved by the regional ethics committee (Centrale Commissie Mensgebonden Onderzoek: CMO Regio Arnhem Nijmegen; 2008/163; ABR: NL23894.091.08) and the medical ethical committee of the VU University Medical Center. These participants were recruited at VU University Amsterdam, Amsterdam, and Radboud University Medical Center, Nijmegen. All participants were evaluated with semi-structured diagnostic interviews for assessing ADHD, oppositional defiance disorder (ODD) and conduct disorder (CD). For further details on diagnostic assessment, see von Rhein et al. (12). Inclusion criteria were age between 8 and 30 years, European Caucasian descent, intelligence quotient (IQ) greater than or equal to 70, and no diagnosis of autism, epilepsy, general learning difficulties, brain disorders, and known genetic disorders. Only unrelated patients with a diagnosis of ADHD at age ≥18 years were included.

IMpACT Brazil

Patients with adult ADHD were ascertained in the ADHD Outpatient Program at the Hospital de Clínicas de Porto Alegre (HCPA), from 2003 and 2013. The study was approved by the Ethics Committee of the Hospital de Clínicas de Porto Alegre. The inclusion criteria were as follows: (a) native Brazilians of European descent (13), (b) aged 18 years or older and (c) fulfilment of the diagnostic criteria for ADHD according to the Diagnostic and Statistical Manual of Mental Disorders (DSM-IV) (APA, 1994), both currently and during childhood (assessed retrospectively). The exclusion criteria were: (a) evidence of clinically significant neurologic diseases that might affect cognition (e.g., delirium, dementia, epilepsy, head trauma and multiple sclerosis), (b) current or past history of psychosis and (c) intelligence quotient (IQ)<70. The semi-structured interview K-SADS-E (Schedule for Affective Disorders and Schizophrenia for School-Age Children-Epidemiologic version), adapted to adults, was used to evaluate ADHD and oppositional defiant disorder (ODD) (14, 15). The Structured Clinical Interview for DSM-IV (SCID-IV-R) was used for the Axis I psychiatric disorders and Mini-International Psychiatric Interview (MINI) for the diagnosis of Antisocial Personality Disorder. Controls were evaluated in a blood donation center at the same hospital as the ADHD patients and matched for several demographic variables. The inclusion criteria included being both a native Brazilian of European descent and 18 years of age or older. We used positive screening on the Adult ADHD Self-Report Scale screener—ASRS-v1.1 (16) as exclusion criteria for controls. Psychiatric disorders other than ADHD we evaluated with the Structured Clinical Interview for DSM-IV screening module—SCID-I/P for the Axis I psychiatric disorders (17). A subset of the control group also underwent a more comprehensive protocol including all instruments applied to the aADHD sample.

IMpACT UK

Patients were attending a National Adult ADHD Outpatient Clinic at the South London and Maudsley NHS Trust, London, UK. Self-report and informant based versions of the Barkley Adult ADHD Rating Scale (BAARS-IV) were used to measure ADHD symptoms. Consisting of 18 DSM-IV items related to inattention and hyperactivity–impulsivity, respondents indicated how frequently they experienced behaviours on a scale of 0 to 3 (never or rarely, sometimes, often, very often) during the past 6 months. Total scores were calculated for each symptom dimension. Informant ratings were provided by a family member or close friend. Diagnosis was based on psychiatric interview and the Conners Adult ADHD Diagnostic Interview for DSM-IV (CAADID) completed by a psychiatrist and a community psychiatric nurse. The CAADID is a structured interview divided into Part I (Patient History Questionnaire) and Part II (Diagnostic Criteria Interview), which are administered separately. Each of the 18 items is scored “yes”, if the behavioral symptom is present *often* within the past 6 months and outcomes are total current ADHD symptom score, and separate totals for inattentive and hyperactive-impulsive symptom domains. Participants attending assessment appointments were consented by a member of the SLaM BRC Bioresource for Mental Health team following the receipt of an information sheet and a detailed explanation of the BRC Bioresource initiative. Ethical approval was granted by the National Research Ethics Committee, London (12/LO/07990). Wellcome Trust Case Control Consortium (WTCCC) control allele frequencies were available from the 1958 Birth Cohort (<http://diagram-consortium.org/uk-exome-chip/>) of 5963 controls (ethical approval 05/ Q0106/74). All samples were processed with the same standard operating procedure and quality control (18).

REFERENCES

1. Reif A, Jacob CP, Rujescu D, Herterich S, Lang S, Gutknecht L, et al. Influence of functional variant of neuronal nitric oxide synthase on impulsive behaviors in humans. Arch Gen Psychiatry. 2009;66(1):41-50.

2. Franke B, Vasquez AA, Johansson S, Hoogman M, Romanos J, Boreatti-Hummer A, et al. Multicenter analysis of the SLC6A3/DAT1 VNTR haplotype in persistent ADHD suggests differential involvement of the gene in childhood and persistent ADHD. Neuropsychopharmacology. 2010;35(3):656-64.

3. Schmermund A, Mohlenkamp S, Stang A, Gronemeyer D, Seibel R, Hirche H, et al. Assessment of clinically silent atherosclerotic disease and established and novel risk factors for predicting myocardial infarction and cardiac death in healthy middle-aged subjects: rationale and design of the Heinz Nixdorf RECALL Study. Risk Factors, Evaluation of Coronary Calcium and Lifestyle. Am Heart J. 2002;144(2):212-8.

4. Halmoy A, Fasmer OB, Gillberg C, Haavik J. Occupational outcome in adult ADHD: impact of symptom profile, comorbid psychiatric problems, and treatment: a cross-sectional study of 414 clinically diagnosed adult ADHD patients. J Atten Disord. 2009;13(2):175-87.

5. Holmen TL, Bratberg G, Krokstad S, Langhammer A, Hveem K, Midthjell K, et al. Cohort profile of the Young-HUNT Study, Norway: a population-based study of adolescents. Int J Epidemiol. 2014;43(2):536-44.

6. Holmen OL, Zhang H, Fan Y, Hovelson DH, Schmidt EM, Zhou W, et al. Systematic evaluation of coding variation identifies a candidate causal variant in TM6SF2 influencing total cholesterol and myocardial infarction risk. Nat Genet. 2014;46(4):345-51.

7. Sanchez-Mora C, Ramos-Quiroga JA, Bosch R, Corrales M, Garcia-Martinez I, Nogueira M, et al. Case-control genome-wide association study of persistent attention-deficit hyperactivity disorder identifies FBXO33 as a novel susceptibility gene for the disorder. Neuropsychopharmacology. 2015;40(4):915-26.

8. Onnink AM, Zwiers MP, Hoogman M, Mostert JC, Dammers J, Kan CC, et al. Deviant white matter structure in adults with attention-deficit/hyperactivity disorder points to aberrant myelination and affects neuropsychological performance. Prog Neuropsychopharmacol Biol Psychiatry. 2015;63:14-22.

9. Kooij JJ, Buitelaar JK, van den Oord EJ, Furer JW, Rijnders CA, Hodiamont PP. Internal and external validity of attention-deficit hyperactivity disorder in a population-based sample of adults. Psychol Med. 2005;35(6):817-27.

10. Franke B, Hoogman M, Arias Vasquez A, Heister JG, Savelkoul PJ, Naber M, et al. Association of the dopamine transporter (SLC6A3/DAT1) gene 9-6 haplotype with adult ADHD. Am J Med Genet B Neuropsychiatr Genet. 2008;147B(8):1576-9.

11. Hoogendoorn EH, Hermus AR, de Vegt F, Ross HA, Verbeek AL, Kiemeney LA, et al. Thyroid function and prevalence of anti-thyroperoxidase antibodies in a population with borderline sufficient iodine intake: influences of age and sex. Clin Chem. 2006;52(1):104-11.

12. von Rhein D, Mennes M, van Ewijk H, Groenman AP, Zwiers MP, Oosterlaan J, et al. The NeuroIMAGE study: a prospective phenotypic, cognitive, genetic and MRI study in children with attention-deficit/hyperactivity disorder. Design and descriptives. Eur Child Adolesc Psychiatry. 2015;24(3):265-81.

13. Polina ER, Rovaris DL, de Azeredo LA, Mota NR, Vitola ES, Silva KL, et al. ADHD diagnosis may influence the association between polymorphisms in nicotinic acetylcholine receptor genes and tobacco smoking. Neuromolecular Med. 2014;16(2):389-97.

14. Mercadante M, Asbahr F, Rosário M, Ayres A. K-SADS, entrevista semi-estruturada para diagnóstico em psiquiatria da infância, versão epidemiológica. FMUSP: São Paulo, SP. 1995.

15. Grevet E, Bau C, Salgado C, Fischer A, Victor M, Garcia C, et al. Interrater reliability for diagnosis in adults of attention deficit hyperactivity disorder and oppositional defiant disorder using K-SADS-E. Arquivos de Neuro-Psiquiatria 2005;63:307-10.

16. Kessler R, Adler L, Barkley R, Biederman J, Conners C, Faraone SV, et al. Patterns and predictors of attention-deficit/hyperactivity disorder persistence into adulthood: results from the national comorbidity survey replication. Biological Psychiatry. 2005;57:1442-51.

17. First M, Spitzer R, Gibbon M, Williams J. Structured Clinical Interview for DSM-IV Axis I Disorders, Patient Edition (SCID-I/P, Version 2.0, 8/98 revision). . Biometrics Research Department, New York State Psychiatric Institute: New York, NY. 1998.

18. Wellcome Trust Case Control C. Genome-wide association study of 14,000 cases of seven common diseases and 3,000 shared controls. Nature. 2007;447(7145):661-78.

Representation of IMpACT samples relative to HapMap populations


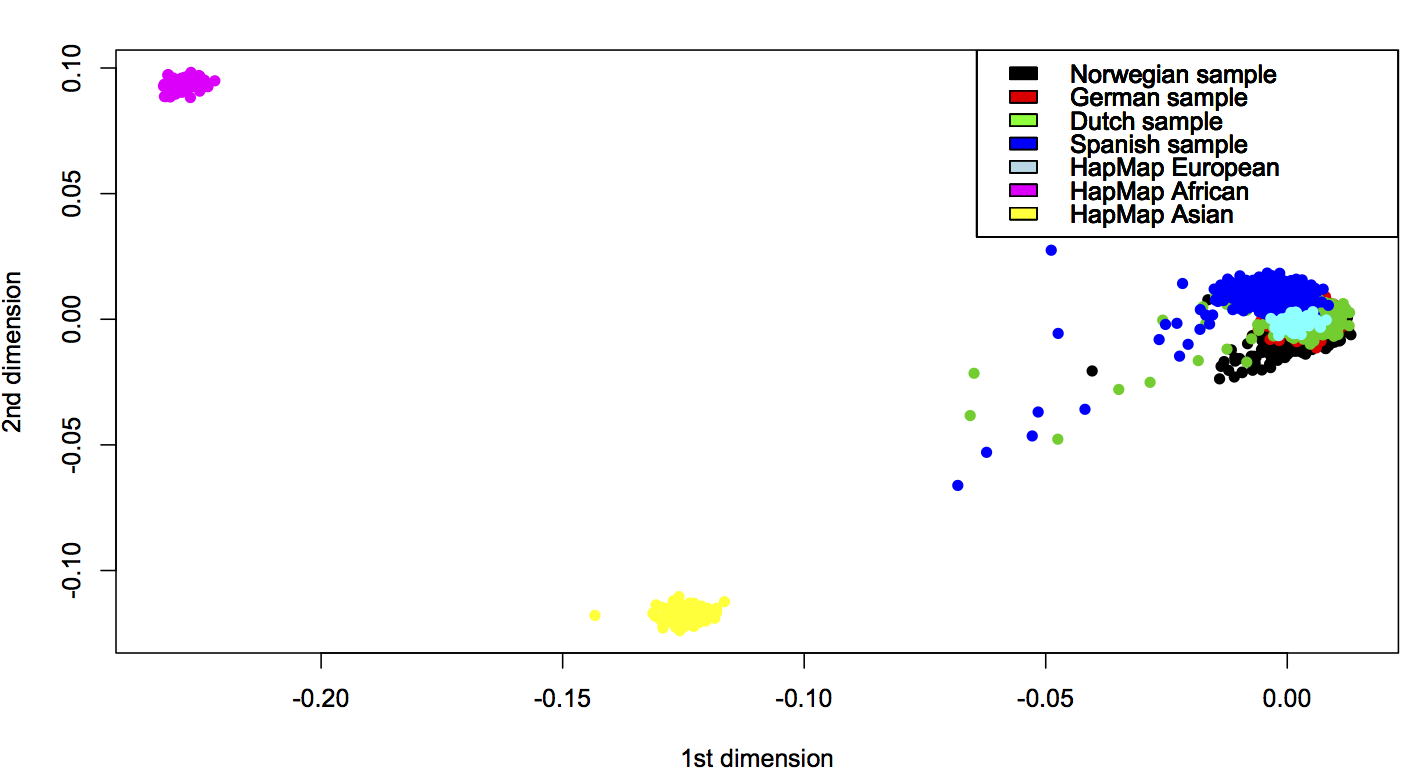

Supplement: Supplementary Appendix 1 [file tp2016196x14.docx]
